# NEOSCOPE: A randomised phase II study of induction chemotherapy followed by oxaliplatin/capecitabine or carboplatin/paclitaxel based pre-operative chemoradiation for resectable oesophageal adenocarcinoma

**DOI:** 10.1016/j.ejca.2016.11.031

**Published:** 2017-03

**Authors:** Somnath Mukherjee, Christopher Nicholas Hurt, Sarah Gwynne, David Sebag-Montefiore, Ganesh Radhakrishna, Simon Gollins, Maria Hawkins, Heike I. Grabsch, Gareth Jones, Stephen Falk, Ricky Sharma, Andrew Bateman, Rajarshi Roy, Ruby Ray, Jo Canham, Gareth Griffiths, Tim Maughan, Tom Crosby

**Affiliations:** a CRUK MRC Oxford Institute for Radiation Oncology Gray Laboratories, Oxford University, Oxford, OX3 7DQ, UK; b Centre for Trials Research, Cardiff University, 6th Floor, Neuadd Meirionnydd, Heath Park, Cardiff, CF14 4YS, UK; c South West Wales Cancer Centre, Swansea, SA2 8QA, UK; d St James's Institute of Oncology, University of Leeds, Cancer Research UK Leeds Centre, St James's University Hospital, Leeds, LS9 7TF, UK; e North Wales Cancer Treatment Centre, Rhyl, LL18 5UJ, UK; f GROW School for Oncology and Developmental Biology, Department of Pathology, Maastricht University Medical Centre, Maastricht, Netherlands; g Section of Pathology and Tumour Biology, Leeds Institute of Cancer and Pathology, University of Leeds, UK; h Velindre Cancer Centre, Velindre Hospital, Velindre Road, Cardiff, CF14 2TL, UK; i Bristol Haematology and Oncology Centre, Bristol, BS2 8ED, UK; j Faculty of Medicine, University of Southampton, Southampton General Hospital, Tremona Road, Southampton, SO16 6YD, UK; k Diana Princess of Wales Hospital, Scartho Road, Grimsby, South Humberside, DN33 2BA, UK; l Southampton Clinical Trials Unit, University of Southampton, Southampton General Hospital, Tremona Road, Southampton, SO16 6YD, UK

**Keywords:** Oesophageal, Chemotherapy, Radiotherapy, Surgery, Neo-adjuvant, Randomised phase II

## Abstract

**Background:**

Oxaliplatin-capecitabine (OxCap) and carboplatin-paclitaxel (CarPac) based neo-adjuvant chemoradiotherapy (nCRT) have shown promising activity in localised, resectable oesophageal cancer.

**Patients and methods:**

A non-blinded, randomised (1:1 via a centralised computer system), ‘pick a winner’ phase II trial. Patients with resectable oesophageal adenocarcinoma ≥ cT3 and/or ≥ cN1 were randomised to OxCapRT (oxaliplatin 85 mg/m^2^ day 1, 15, 29; capecitabine 625 mg/m^2^ bd on days of radiotherapy) or CarPacRT (carboplatin AUC2; paclitaxel 50 mg/m^2^ day 1, 8, 15, 22, 29). Radiotherapy dose was 45 Gy/25 fractions/5 weeks. Both arms received induction OxCap chemotherapy (2 × 3 week cycles of oxaliplatin 130 mg/m^2^ day 1, capecitabine 625 mg/m^2^ bd days 1–21). Surgery was performed 6–8 weeks after nCRT. Primary end-point was pathological complete response (pCR). Secondary end-points included toxicity, surgical morbidity/mortality, resection rate and overall survival.

**Statistics:**

Based on pCR ≤ 15% not warranting future investigation, but pCR ≥ 35% would, 76 patients (38/arm) gave 90% power (one-sided alpha 10%), implying that arm(s) having ≥10 pCR out of first 38 patients could be considered for phase III trials. ClinicalTrials.gov: NCT01843829. Funder: Cancer Research UK (C44694/A14614).

**Results:**

Eighty five patients were randomised between October 2013 and February 2015 from 17 UK centres. Three of 85 (3.5%) died during induction chemotherapy. Seventy-seven patients (OxCapRT = 36; CarPacRT = 41) underwent surgery. The 30-d post-operative mortality was 2/77 (2.6%). Grade III/IV toxicity was comparable between arms, although neutropenia was higher in the CarPacRT arm (21.4% versus 2.6%, p = 0.01). Twelve of 41 (29.3%) (10 of first 38 patients) and 4/36 (11.1%) achieved pCR in the CarPacRT and OxcapRT arms, respectively. Corresponding R0 resection rates were 33/41 (80.5%) and 26/36 (72.2%), respectively.

**Conclusion:**

Both regimens were well tolerated. Only CarPacRT passed the predefined pCR criteria for further investigation.

## Highlights

• Oxaliplatin-capecitabine (OxCap) and carboplatin-paclitaxel (CarPac) based neo-adjuvant chemoradiotherapy (nCRT) have shown promising activity in localised, resectable oesophageal cancer.
• A randomised phase II ‘pick a winner’ trial was conducted to assess which regimen to take forward to a phase III trial.
• Both regimens were well tolerated. Only CarPacRT passed the predefined pathological complete response (pCR) criteria for further investigation.

## 1 Introduction

Treatment by surgery alone confers poor outcome in patients with resectable oesophageal cancer. Neo-adjuvant chemoradiotherapy (nCRT) preceding surgery improves disease-specific survival. Efforts to improve these outcomes have focussed on the addition of adjuvant chemotherapy and/or radiotherapy to surgical treatment. However, most of the randomised studies evaluating nCRT were performed over two decades ago, were heterogenous in design, often under-powered, largely tested platinum/fluropyrimidine-based regimens, and reported a high incidence of treatment-related toxicity and post-operative mortality [1]. Recently, the CROSS trial, which showed that nCRT was associated with a doubling of median overall survival (OS) to 49.4 months compared to surgery alone, has established a new standard of care [2]. In that study, the incidence of grade III–IV haematological and non-haematological toxicity (7% and 13%, respectively) in the nCRT arm was one of the lowest reported in the literature and post-operative mortality (4%) was identical to the surgery-only arm.

In the United Kingdom (UK), due to concerns regarding increased post-operative morbidity, clinicians favoured the use of neo-adjuvant or peri-operative chemotherapy alone in patients with locally advanced, resectable, gastro-oesophageal cancer (MAGIC [3], OE05 [4], ST03 [5], NOGCA [6]). However, advances in radiotherapy techniques, demonstration of enhanced radiotherapy quality assurance through the SCOPE trial [7], and centralisation of gastro-oesophageal surgery encouraged the Upper GI Clinical Studies Group of the National Cancer Research Institute and Cancer Research UK to support and fund the first multicenter study of pre-operative CRT in the UK.

Oxaliplatin has been shown to be comparable in efficacy to cisplatin in advanced gastro-oesophageal cancer and can be conveniently delivered as a 2-h infusion [8]. Single arm phase I–II studies have demonstrated feasibility of oxaliplatin-based nCRT [9], [10], [11], [12], [13], [14], [15]. One randomised phase II study comparing oxaliplatin-5FU-based CRT with cisplatin 5FU-based CRT in patients with inoperable but localised oesophageal cancer has demonstrated no significant difference in survival or toxicity but the ease of administration of oxaliplatin over cisplatin makes it a more attractive option [16]. Thus we have chosen to investigate an oxaliplatin rather than cisplatin-based platinum-fluoropyrimidine regimen.

NEOSCOPE was a randomised phase II trial that evaluated the efficacy and toxicity of OxCapRT and CarPacRT in the pre-operative treatment of patients with locally advanced resectable oesophageal cancer and assessed the feasibility of safely introducing nCRT into clinical practice in the UK. We felt it was necessary to evaluate the two regimens as there was a growing international shift to the use of CarPacRT based on historic comparison with the traditional platinum-fluoropyrimidine regimens rather than through a randomised trial. The aim was to ‘pick a winner’ that could be taken forward to a future Phase III trial where neo-adjuvant CRT would be compared with pre-operative chemotherapy.

## 2 Methods

In this multicenter, randomised, open-label, ‘pick a winner’, phase II trial, we recruited patients who fulfilled the following key eligibility criteria: resectable adenocarcinoma (ACA) of the oesophagus including Siewert type 1 or 2 tumour of the gastro-oesophageal junction (GEJ) (maximum extension beyond GEJ of 3 cm), with cT stage ≥ 3 and/or cN stage ≥ 1, World Health Organisation performance status 0–1, maximum disease (T + N) length 8 cm, adequate respiratory, cardiac, haematological, renal and hepatic function and ≥18 years old. Staging investigations included contrast-enhanced computed tomography (CT) scan of thorax and abdomen, endoscopic ultrasound (EUS), positron-emission tomography (PET)/CT scan, and laparoscopy (for lower third and GEJ tumours). The study protocol was published previously [17] and is included as Supplementary Material.

All patients provided written informed consent to a medical doctor and research nurse who then telephoned the Wales Cancer Trials Unit (WCTU) to randomly assign (1:1) the patients to OxCapRT or CarPacRT by stratified minimisation with a random element (80:20) via a centralised computer system. Randomisation was stratified by recruiting hospital, cT stage (T1/T2 versus T3/T4), and cN stage (N0 versus N+ve).

Induction chemotherapy (ICT) in both arms consisted of two 3-weekly cycles of oxaliplatin (130 mg/m^2^ intravenously on day 1) and capecitabine (625 mg/m^2^ orally twice daily from day 1 to day 21). During the CRT phase, patients randomly assigned to the OxCapRT arm received oxaliplatin (85 mg/m^2^ on intravenously on days 1, 15, 29) and capecitabine (625 mg/m^2^ bd orally on days of radiotherapy). Patients assigned to the CarPacRT arm received carboplatin AUC2 and paclitaxel 50 mg/m^2^ with both drugs administered intravenously on days 1, 8, 15, 22, 29 of radiotherapy. Capecitabine tablets could be dissolved for patients with swallowing difficulties.

The radiotherapy was planned using intravenous contrast CT simulation with minimum 3-mm CT slices. 45 Gy in 25 daily fractions, prescribed according to recommendations of the International Commission on Radiation Units and Measurements (ICRU-50), was delivered Monday to Friday as a 3D, conformally planned single-phase treatment, usually with four radiotherapy fields. Gross tumour volume (GTV) was defined using diagnostic CT scan, endoscopy, EUS and PET scan (when available). The clinical target volume (CTV) was calculated by growing the GTV by 2 cm manually along the oesophagus superiorly–inferiorly and 1 cm radially, editing out lungs and bronchus, heart, liver, aorta and vertebrae. The planning target volume was created by growing CTV 1 cm superiorly–inferiorly and 0·5 cm radially. 4D CT simulation was encouraged for lower oesophagus/GEJ tumours (included as Supplementary Material: NEOSCOPE Radiotherapy Treatment Planning and Delivery document). Cone-Beam CT verification was used on the first 3 fractions of radiotherapy treatment and weekly thereafter. The Radiotherapy Trials Quality Assurance (RTQA) process has been previously described and included a pre-accrual component, and on-trial real-time or timely retrospective review [17], [18].

Toxicity was assessed as per US National Cancer Institute's Common Terminology Criteria for Adverse Events (CTCAE version 4.03). Capecitabine compliance was assessed by tablet count at each visit. Restaging CT/PET-CT was undertaken 4–6 weeks after CRT and surgery was performed at 6–8 weeks after completion of CRT. Type of surgery was not mandated. The resection specimens were evaluated by the local pathologists as per detailed trial-specific guidelines (see appendix 4 of the trial protocol). Post-surgical assessments for toxicity, post-operative morbidity and review of disease status were performed at 30 d, 6 months and 12 months following surgery. Investigations and follow up beyond 12 months were done as per institutional standard. The choice of treatment at relapse was left to the discretion of the treating clinician.

The primary end-point was pathological complete response (pCR) as reported by the local pathologists according to the trial-specific guidelines referred to above. pCR was defined as complete absence of tumour in the whole resected specimen (ypT0N0). Cases with residual primary tumour were graded using the Mandard tumour regression grading system [19]. Secondary end-points were feasibility of recruitment, toxicity (CTCAE version 4.03), peri-operative morbidity/mortality, circumferential resection margin positivity rate, and overall survival. A resection margin was defined as being positive when tumour cells were present directly at the resection margin or within 1 mm of the resection margin.

The study was not powered for a direct comparison between arms. The sample size calculations were based on the maximum of two binomial random variables and followed the methodology described by Dunnett [20] with input from the MRC North West Hub for Trials Methodology Research (see Appendix A). A pCR of 15% was not considered large enough to warrant further investigation, whilst a pCR of 35% was considered worthwhile. The null hypothesis was that pCR1 = pCR2 = 0.15 where pCR1 and pCR2 are the response rates for the two treatments. A sample size of 76 (38 patients/arm) gave a one-sided type I error of 10% and a power of 90% of achieving significance if patients on one treatment had a response rate of 35% whilst those on the other had response rate of 15%. A priori rules were specified to decide whether or not one or both trial arm(s) warranted future investigation in phase III trials (Supplementary Table 1) [17]. The study sought to recruit 85 patients to allow for a potential 10% drop-out rate before resection.

Data were analysed according to a pre-specified analysis plan using the Stata SE 14 statistical package at the time the primary end-point had been collected in all patients (further follow up is ongoing). All analyses were by intention to treat except the toxicity analyses which were conducted only in those patients who had some treatment during the related treatment phase and the surgical complications analysis only in those who had surgery. Proportions were compared using chi-square tests. Clopper–Pearson exact binomial method was used to calculate confidence intervals for the primary end-point.

The trial protocol was approved by the UK Medicines and Healthcare products Regulatory Agency and a Multi-Centre Research Ethics Committee, sponsored by Velindre NHS Trust and coordinated by the WCTU at Cardiff University.

## 3 Results

**Fig. 1:**
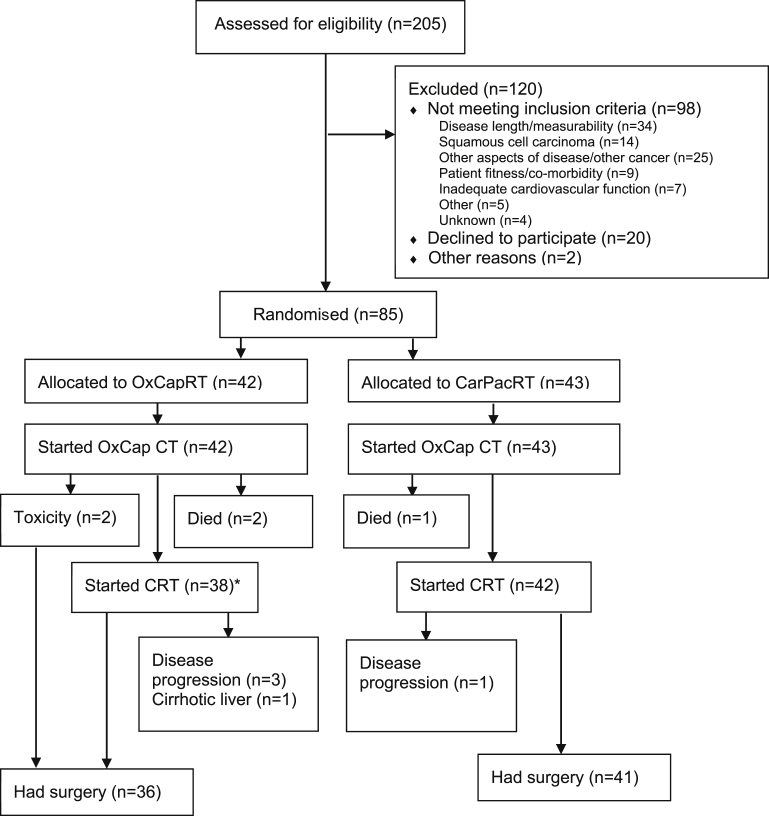
CONSORT flow diagram of trial participants. *Three of the patients allocated to OxCapRT were actually given CarPacRT due to toxicities during induction chemotherapy.

Eighty-five patients were randomised from 17 UK centres between 10 October 2013 and 12 February 2015 (Fig. 1). At the time of analysis, all patients had completed 30-d post-operative assessment or died or withdrawn from the study. Patient and tumour baseline characteristics were balanced between the groups (Table 1).

Toxicities during induction chemotherapy and nCRT are shown in Table 2. There were 3 deaths (all serious adverse reactions) during induction OxCap chemotherapy (3/85, 3.5%): two (1 multiple organ failure following chemotherapy induced diarrhoea and one secondary to diarrhoea/acute ischaemic leg) in patients randomised to the OxCapRT arm, and one (superior mesenteric artery thrombus and small bowel ischaemia) in patients randomised to the CarPacRT arm. There was no difference in the rate of any grade III–IV toxicities between the arms during induction chemotherapy. During CRT, the rate of any grade III–IV toxicity (52.4% [22/42] versus 42.1% [16/38], χ^2^ = 0.8447, *p* = 0.358) and rate of haematological grade III–IV toxicity (28.6% [12/42] versus 15.8% [6/38], χ^2^ = 1.8692, *p* = 0.172) were higher in the CarPacRT arm, although neither reached statistical significance. There was significantly higher incidence of grade III–IV neutropenia in the CarPacRT arm (21.4% [9/42] versus 2.6% [1/38], *post hoc* χ^2^ = 6.4447, *p* = 0.011), though febrile neutropenia was uncommon.

**Fig. 2:**
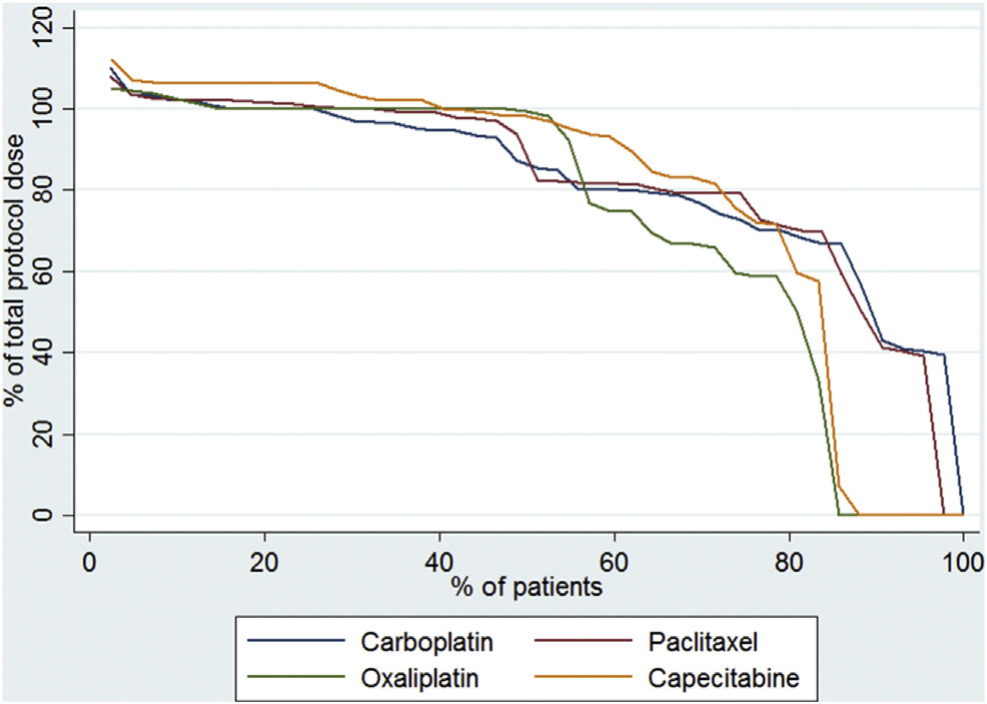
Percent of total chemotherapy dose during CRT (n = 85).

During induction chemotherapy, the median percentage of protocol dose of oxaliplatin:capecitabine was 100 interquartile range (IQR: 98–101):97 (IQR: 92–104) and 100 (IQR: 96–100):100 (IQR: 93–104) in the OxCapRT and CarPacRT arms, respectively. During CRT (Fig. 2), the median percentage of protocol dose of oxaliplatin:capecitabine was 99 (IQR: 59–100):98 (IQR: 72–106) and the median percentage of protocol dose of carboplatin:paclitaxel was 85 (IQR:70–100):83 (IQR: 73–101). One patient in each arm did not receive cycle two of induction chemotherapy due to toxicities (1 chest pain, 1 multiple toxicities). There was only one treatment delay during induction chemotherapy due to a haematological toxicity in the OxCapRT arm. Four patients (2 deaths, 2 toxicities) in the OxCapRT and one (death) in the CarPacRT arm did not start CRT. Additionally, three patients initially randomised to the OxCapRT arm were withdrawn from trial treatment due to toxicities during induction chemotherapy (they were actually given CarPacRT instead but were not counted in that arm for this analysis). These imbalances are the reason for the higher proportion of patients receiving no chemotherapy drugs in the OxCapRT arm during CRT.

All centres passed pre-trial quality assurance (QA) prior to entering patients into the trial. Eighty-three radiotherapy (RT) contours and plans were reviewed (this included three patients who ultimately did not undergo CRT), 39 (47%) underwent prospective review prior to start of RT and 44 (53%) underwent timely retrospective review wherein feedback was provided by the 3rd fraction of RT. Eight cases required re-submission due to unacceptable contours or planning, six of whom were either the first or second patient recruited from the participating centre.

Compliance to radiotherapy was similar across arms with 38 (90.5%) and 40 (93.0%) patients receiving the full protocol dose in the OxCapRT and CarPacRT arms, respectively (χ^2^ = 0.1824, *p* = 0.669). Two patients in the CarPacRT did not complete radiotherapy, one due to patient choice (43.2 Gy/24#) and one due to gastrointestinal haemorrhage (30.6 Gy/17#). Time from start of randomisation to start of CRT: median of 47 d (IQR: 45–52) in the OxCapRT arm and 47 d (IQR: 45–49) in the CarPacRT arm.

Thirty-six (85.7%) and 41 (95.3%) patients had surgery in the OxCapRT and CarPacRT arms, respectively (Fig. 1, Table 3). There was one death within 30 d post-surgery in each trial arm and 30-d post-operative complication rates were very similar: 19 (54.3%) and 21 (51.2%) patients in the OxCapRT and CarPacRT arms, respectively (χ^2^ = 0.0712, *p* = 0.790).

The results of the local pathological resection specimen assessment are shown in Table 4. Four of 42 patients (8.5%, 80% confidence intervals [CIs]: 4.0–18.0; 4/36 [11.1%] of those who had surgery) in the OxCapRT arm had pCR, thus the phase II target of 10 out of 38 patients was not reached. In the CarPacRT arm, 10 out of the first 38 and 12 of 43 (27.9%, 80% CIs: 19.0–38.5; 12/41 [29.3%] of those who had surgery) had a pCR, thus passing the phase II target. The rate of R0 resections also favoured the CarPacRT arm (33/41 [80.5%] versus 26/36 [72.2%]) although the study was not powered for direct comparisons.

The survival data are still immature and will be presented after longer term follow up.

## 4 Discussion

This phase II trial demonstrated activity warranting further study using the CarPacRT regimen only. Both CRT regimens were well tolerated and post-operative morbidity was comparable between arms (around 50%). However, neutropenia during CRT was significantly higher in the carboplatin-paclitaxel arm (21.4% versus 2.6%, *p* = 0.01), although this did not translate into increased risk of mortality. Post-operative mortality was low in both arms (around 2.5%). This study also demonstrated, through the use of a detailed protocol and robust quality assurance programme, the safety and feasibility of introducing nCRT into clinical practice in the United Kingdom.

The rationale for ICT before CRT was to deliver additional systemic therapy in a disease where systemic relapse is common, and where only half the patients manage chemotherapy following surgical or radiotherapy treatment [3], [5], [21]. However, since the inception of the NEOSCOPE trial, two randomised phase II trials testing the role of ICT have failed to demonstrate an increase in OS in the ICT arm [22], [23]. In the study by Yoon *et al.*, ICT also led to increased incidence of grade III–IV thrombocytopaenia and reduction of dose intensity, similar to the increase in haematological toxicity seen in the CarPacRT arm of NEOSCOPE. Conversely, the rates of pCR were similar in this study compared with the CROSS trial where ICT was not used [2].

To the best of the authors' knowledge, this is the first multi-centre study to routinely utilise image-guided radiotherapy with detailed 4D CT planning instructions and cone-beam verification supported by high quality peer reviewed real-time RTQA in the context of neo-adjuvant chemoradiation for oesophageal ACA [24]. This study supports the feasibility of implementing complex image-guided RT and will be taken forward in the context of future oesophageal cancer radiotherapy studies (e.g. SCOPE-2).

Given the doseresponse effect of radiotherapy in nCRT [25], we used 45 Gy instead of the 41.4 Gy which was used in the CROSS trial. The low mortality and morbidity rate in this trial is reassuring—future trials (accompanied by high quality RTQA programmes) may consider 45 Gy to be a safe dose to deliver and may even consider further dose escalation or use of hypofractionated radiotherapy.

We undertook this trial in patients with ACA only as this is the predominant (or increasingly prevalent) cancer subtype in Western populations. ACA may have a different biology as well as different chemo- and radio-sensitivity compared to squamous cell cancer (SCC), and CROSS trial had shown different pCR rates between ACA and SCC. We believe that future trials of nCRT should be performed separately for ACA and SCC.

Other than true lack of efficacy or due to small numbers, we are unable to explain the reason for the low pCR rate in the OxCapRT arm. Previous phase I/II studies have quoted a pCR rate of 27.3–38% in resected patients [4], [5], [24] and the translational aspect of NEOSCOPE will aim to characterise genetic/molecular markers that defined the OxCapRT-responsive group.

This study has its limitations. Although this study was randomised, a higher proportion of patients allocated to the OxCapRT arm did not undergo surgery (6 versus 1); additionally, three patients originally allocated to the OxCapRT arm crossed over to the CarPacRT arm due to toxicity during ICT (analysed as intention-to-treat). Whereas such instances are inevitable in clinical trials, the shift of patients may have had an impact on the pCR rate in the trial arms, given the small number of patients in the trial. Additionally, the trial was not powered to detect differences in pCR between arms. A further limitation of this study is that the data published here relied on local pathological assessment. However, the trial protocol included detailed guidance on the specimen work up and reporting and we plan to conduct central pathological assessment once the sample collection is complete.

In summary, NEOSCOPE supports further investigation of CarPacRT for oesophageal nCRT, but OxCapRT failed to pass the pre-specified pCR threshold. Neo-adjuvant OxCapRT and CarPacRT can be both delivered with radiotherapy to a dose of 45 Gy with acceptable toxicity and low incidence of post-operative 30-d mortality, although induction chemotherapy may not be necessary. High quality, image-guided CRT prior to surgery can be used safely in the UK.

## Funding

This work was supported by Cancer Research UK (grant number: C44694/A14614).

## Conflict of interest statement

None declared.

## Figures and Tables

**Table 1:** Baseline characteristics.

		OxCapRT (*n* = 42)		CarPacRT (*n* = 43)	
		*n*	%	*n*	%
Sex	Male	36	85.7	33	76.7
	Female	6	14.3	10	23.3
Age	Median (IQR, range)	65 (58–72, 46–77)		64 (61–68, 29–76)	
cT stage	T2	6	14.3	3	7.0
	T3	36	85.7	37	86.0
	T4a	0	0.0	3	7.0
cN stage	N0	12	28.6	16	37.2
	N1	21	50.0	20	46.5
	N2	8	19.0	6	14.0
	N3	1	2.4	1	2.3
Site of predominant tumour	Middle third (24 ≤ 32 cm)	6	14.3	2	4.7
	Lower third (32–40 cm)	32	76.2	39	90.7
	Missing	4	9.5	2	4.7
Time from staging scan to randomisation (d)	Median (IQR, range)	27 (19–39, 8–56)		28 (23–34, 2–51)	
Maximum total disease length from EUS, PET and CT	Median (IQR, range)	5.85 (4.7–6,2–8)		5.7 (5–7, 2–8.3)	
WHO performance status	0	37	88.1	35	81.4
	1	5	11.9	8	18.6
Time from randomisation to start of treatment (d)	Median (IQR, range)	4 (2–6, 0–18)		4 (3–6, 0–14)	

**Table 2:** Grade III/IV CTCAE toxicities during treatment.

System organ class	Adverse event	Induction chemotherapy		CRT			
		Both arms (*n* = 85)		OxCapRT (*n* = 38)		CarPacRT (*n* = 42)	
		*n*	%	*n*	%	*n*	%
Any toxicity		27	31.8	16	42.1	22	52.4
Any haematological toxicity		2	2.4	6	15.8	12	28.6
Blood and lymphatic system disorders	Anaemia	1	1.2	1	2.6	0	0.0
	Febrile neutropenia	0	0	0	0.0	1	2.4
Cardiac	Chest pain	2	4.8	0	0.0	0	0.0
Gastrointestinal	Any in this class	15	17.6	5	13.2	8	19.0
	Abdominal pain	2	2.4	1	2.6	0	0.0
	Colonic spasm	0	0.0	1	2.6	0	0.0
	Constipation	0	0.0	1	2.6	0	0.0
	Diarrhoea	7	8.2	0	0.0	1	2.4
	Dry mouth	1	1.2	1	2.6	0	0.0
	Dysphagia	6	7.1	2	5.3	2	4.8
	GI haemorrhage	0	0.0	0	0.0	1	2.4
	Mucositis	1	1.2	0	0.0	0	0.0
	Nausea/vomiting	6	7.1	0	0.0	0	0.0
	Oesophageal pain	0	0.0	0	0.0	1	2.4
	Oesophagitis	1	1.2	2	5.3	2	4.8
General disorders	Fatigue	9	10.6	4	10.5	6	14.3
Injury	Fall	0	0.0	1	2.6	0	0.0
Investigations	Lymphocyte decrease	0	0.0	3	7.9	3	7.1
	Platelet decrease	1	1.2	1	2.6	0	0.0
	Neutrophil decrease	0	0.0	1	2.6	9	21.4
	White blood cell decrease	0	0.0	2	5.3	2	4.8
Metabolism	Anorexia	2	2.4	2	5.3	0	0.0
	Other	4	4.7	0	0.0	0	0.0
Nervous system	Peripheral neuropathy	5	5.9	0	0.0	0	0.0
	Pharyngolaryngeal dysaesthesia	1	1.2	0	0.0	0	0.0
Respiratory	Dyspnoea	1	1.2	0	0.0	1	2.4
Vascular	Hypertension	1	1.2	1	2.6	0	0.0
	Hypotension	0	0.0	0	0.0	1	2.4
	Peripheral ischaemia	1	1.2	0	0.0	0	0.0
	Thromboembolic events	1	1.2	1	2.6	1	2.4

CTCAE, Common Terminology Criteria for Adverse Events.

**Table 3:** Surgery.

		OxCapRT		CarPacRT	
		*n*	%	*n*	%
Patients randomised		42		43	
Patients not having surgery, n (%)		6	14.3	2	4.7
Disease progression		3	7.1	1	2.3
Comorbidity		1	2.4	0	0.0
Died before surgery		2	4.8	1	2.3
Patients having surgery		36	85.7	41	95.3
Days between finishing pre-surgical treatment and surgery, median (n, IQR, range)		52 (36, 47–64,37–92)		56 (41, 49–73,41–147)	
Number of days in hospital post-surgery as an in-patient (d), median (n, IQR, range)		11.5 (36, 9.5–16, 0–74)		12 (40, 10–19,0–67)	
30-d post-operative mortality		1^a^	2.8	1^b^	2.4
30-d post-operative complications^c^	Any complication	19	54.3	21	51.2
	Respiratory complications	14	40.0	15	36.6
	Cardiac complications	9	25.7	4	9.8
	Wound infection	3	8.6	5	12.2
	Chylothorax requiring treatment	1	2.9	2	4.9
	Haemorrhage requiring transfusion or intervention	2	5.7	0	0.0
	Other complications	9	25.7	9	22.0
Anastomotic leak	None	32	88.9	35	85.4
	Radiological/endoscopic	0	0.0	3	7.3
	Missing data	4	11.1	3	7.3

a Multiple organ failure following cardiac arrest. Hospital acquired pneumonia following surgery. Adenocarcinoma of oesophagus.

b Anastomotic leak.

c Uses a denominator of 35 in the OXCAP-CRT arm—one patient has missing complications data.

**Table 4:** Local pathologist findings.

		OxCapRT (*n* = 36)		CarPacRT (*n* = 41)	
		*n*	%	*n*	%
Mandard tumour regression grading	No residual tumour	4	11.1	12	29.3
	Very few residual cancer cells	13	36.1	16	39.0
	Predominant fibrosis with few tumour cells	13	36.1	10	24.4
	Dominant tumour mass with fibrosis and/or vasculopathy	4	11.1	3	7.3
	No histological response	0	0.0	0	0.0
	Not gradeable	1^a^	2.8	0	0.0
	Missing	1	2.8	0	0.0
Circumferential resection margin (CRM) status	Tumour at CRM	2	5.6	3	7.3
	Tumour within 1 mm of CRM	8	22.2	5	12.2
	No tumour within 1 mm	26	72.2	33	80.5
ypT	0	5	13.9	12	29.3
	1a	4	11.1	2	4.9
	1b	8	22.2	7	17.1
	2	2	5.6	4	9.8
	3	17	47.2	16	39.0
ypN	0	23	63.9	31	75.6
	1	6	16.7	9	22.0
	2	6	16.7	0	0.0
	3	1	2.8	1	2.4
Resection margin	0 (No residual disease)	26	72.2	33	80.5
	1 (Microscopic residual disease)	10	27.8	8	19.5

a ypT0, ypN1.

## Footnotes

Appendix A Supplementary data related to this article can be found at http://dx.doi.org/10.1016/j.ejca.2016.11.031.
