# Supplementary material for: NEOSCOPE: A randomised phase II study of induction chemotherapy followed by oxaliplatin/capecitabine or carboplatin/paclitaxel based pre-operative chemoradiation for resectable oesophageal adenocarcinoma
Source: Eur J Cancer. 2017 Mar;74:38–46. doi: 10.1016/j.ejca.2016.11.031 (PMC5341738; doi:10.1016/j.ejca.2016.11.031)
Supplement: Supplementary file 1 [file mmc1.pdf]

NeoSCOPE

NeoSCOPE: Neo-adjuvant Study of Chemoradiotherapy in OesoPhagEal Cancer

A randomised Phase II study of two pre-operative chemoradiotherapy regimens (oxaliplatin and capecitabine followed by radiotherapy with either oxaliplatin and capecitabine or paclitaxel and carboplatin) for resectable oesophageal cancer.

Clinical Trial Protocol

Version: 3.0

Date: 01th May 2015

EudraCT No: 2012-000640-10

Funder: Cancer Research UK

CR-UK reference number: C44694/A14614

Name of Sponsor: Velindre NHS Trust

Authorised by:			
Name:	Tom Crosby	Role:	Chief Investigator
Signature:	[signature]	Date:	20/05/2015
Name:	Chris Hurt	Role:	Scientific Lead
Signature:	[signature]	Date:	20/5/15
Name:	Richard Adams	Role:	Interim Director WCTU
Signature:	[signature]	Date:	20/05/2015

WCTU Wales Cancer Trials Unit Uned Ymchwil Canser Cymru

CANCER RESEARCH UK

NCRI

GIG CYMRU NHS WALES

Ymddiriedolaeth GIG Felindre Velindre NHS Trust

Radiotherapy Trials QA

Developed on behalf of the NCRI Upper GI Clinical Studies Group

CONFIDENTIAL

This material is the property of the Wales Cancer Trials Unit. Do not disclose or use except as authorised.

General Information

This protocol describes the NeoSCOPE clinical trial, and provides information about the procedures for entering participants into the trial. The protocol should not be used as a guide, or as an aide-memoire for the treatment of other patients. Every care has been taken in drafting this protocol; however, corrections or amendments may be necessary. These will be circulated to the known Investigators in the trial, but sites entering patients for the first time are advised to contact the Wales Cancer Trials Unit (WCTU) in Cardiff to confirm that they have the most up-to-date version of the protocol in their possession. Problems relating to the trial should be referred, in the first instance, to the WCTU.

Compliance

This trial will adhere to the conditions and principles of Good Clinical Practice which apply to all clinical trials as outlined in the Medicines for Human Use (Clinical Trials) Regulations 2004 (SI 1031), as amended, EU Directive 2001/20/EC, EU Directive 2005/28/EC. It will be conducted in compliance with the protocol, the Declaration of Helsinki (South Africa, 1996), the Research Governance Framework for Health and Social Care (Welsh Assembly Government 2009 and Department of Health 2nd July 2005), the Data Protection Act 1998, and other regulatory requirements as appropriate.

Funding

The NeoSCOPE trial is being funded by the Clinical Trials Advisory and Awards Committee (CTACC), on behalf of Cancer Research UK.

This trial is supported by Cancer Research UK core funding at the WCTU.

WCTU Randomisation line:
029 2064 5500
(Open Monday – Friday, 9am – 5pm)

N.B. This telephone number is strictly for randomisation and should not be used for general queries.

Serious Adverse Event (SAE) Fax Number:

029 2064 4488

CONFIDENTIAL
This material is the property of the Wales Cancer Trials Unit. Do not disclose or use except as authorised.

Trial Coordination

NeoSCOPE trial is being coordinated by the WCTU, a National Cancer Research Institute (NCRI) accredited, and United Kingdom Clinical Research Collaboration (UKCRC) registered trials unit.

This protocol has been developed by the NeoSCOPE Trial Management Group (TMG) on behalf of the NCRI Upper GI Clinical Studies Group.

Wales Cancer Trials Unit
School of Medicine
Cardiff University
6th Floor
Neuadd Meirionnydd
Heath Park
Cardiff CF14 4YS

Tel: +44 (0) 29 2068 7500
Fax: +44 (0) 29 2068 7501

Email: neoscope@cardiff.ac.uk
Website: www.wctu.org.uk

NeoSCOPE trial staff

For all queries please contact the NeoSCOPE Trial Manager. Any clinical queries will be directed through the Trial Manager to either the Chief Investigator or one of the clinical Co-Investigators.

**Trial Manager:**	**Dr Ruby Ray**	**Tel: +44 (0) 29 2068 7477**<br>**Email:** Al-MokhtarR@cardiff.ac.uk
Associate Director:	Dr Fergus Macbeth	To contact, email Trial Manager
Interim Director:	Dr Richard Adams	Email: Richard.Adams@wales.nhs.uk
Scientific Lead/Senior Statistician:	Chris Hurt	Email: hurtcn@cardiff.ac.uk
Safety Desk		Tel: +44 (0) 29 2068 7469<br>Email: WCTU-safety@cardiff.ac.uk

CONFIDENTIAL
This material is the property of the Wales Cancer Trials Unit. Do not disclose or use except as authorised.

Chief Investigator

Dr Tom Crosby
Consultant Oncologist
Velindre Hospital
Cardiff CF14 2TL

Co-Investigators

Mr Chris Hurt
Scientific Lead/Senior Statistician
Wales Cancer Trials Unit
6th Floor
Neuadd Meirionnydd
Heath Park
Cardiff CF14 4YS

Dr Richard Adams
Interim Director
Wales Cancer Trials Unit
6th Floor
Neuadd Meirionnydd
Heath Park
Cardiff CF14 4YS

Dr Somnath Mukherjee
Consultant Clinical Oncologist
Gray Institute for Radiation Oncology & Biology
University of Oxford
Old Road Campus Research Building
Off Roosevelt Drive
Churchill Hospital
Oxford OX3 7DQ

Dr Simon Gollins
Consultant Clinical Oncologist
North Wales Cancer Treatment Centre
Glan Clwyd Hospital, Bodelwyddan
Rhyl LL18 5UJ

Dr Heike Grabsch
Consultant Histopathologist
Pathology, Anatomy & Tumour Biology
Leeds Institute of Cancer and Pathology Wellcome Trust
Brenner Building
St James's University Hospital
Beckett Street
Leeds LS9 7TF

Dr Andrew Bateman
Consultant Clinical Oncologist
Cancer Sciences
University of Southampton
Tremona Road
Southampton
Hampshire SO16 6YD

Dr Ganesh Radhakrishna
Consultant Clinical Oncologist
St James's Institute of Oncology
Beckett Street
Leeds LS9 7TF

Dr Ricky Sharma
Consultant Clinical Oncologist
Gray Institute for Radiation Oncology & Biology
University of Oxford
Old Road Campus Research Building
Off Roosevelt Drive
Churchill Hospital
Oxford OX3 7DQ

Mr Wyn Lewis
Consultant Surgeon
Cardiff and Vale NHS Trust
University Hospital of Wales
Heath Park
Cardiff CF14 4XW

Dr Sarah Gwynne
Consultant Clinical Oncologist
Singleton Hospital
Sketty Lane
Sketty
Swansea SA2 8QA

Nursing Advisor

Ms Wendy Wade
NISCHR CRC South East Wales Research Network
2nd Floor
12 Cathedral Road
Cardiff CF11 9LJ

Physics Advisers

Mr Rhydian Maggs and Mr Gareth Jones
Radiotherapy Physicists
Cardiff NCRI RTTQA Group
Velindre Hospital
Cardiff, CF14 2TL

Pharmacy Advisor

Ms Bethan Tranter
Pharmacy Department
Velindre Hospital
Cardiff CF14 2TL

Clinical Advisors

Professor David Sebag-Montefiore
Consultant Clinical Oncologist
St James's Institute of Oncology
Beckett Street
Leeds LS9 7TF

Dr Ashley Roberts
Consultant Radiologist
Velindre Hospital
Cardiff CF14 2TL

Professor Tim Maughan
Professor of Clinical Oncology
Gray Institute for Radiation Oncology and Biology
University of Oxford
Old Road Campus Research Building
Roosevelt Drive
Oxford OX3 7DQ

Dr Maria Hawkins
Consultant Clinical Oncologist
The Royal Marsden
Downs Road
Sutton
Surrey SM2 5PT

Consumer representatives

Name
Sue Hadlow
c/o WCTU

Name
Jim Fitzgibbon
c/o WCTU

Table of contents

Abbreviations and glossary....8
1 Trial schema....12
2 Trial synopsis....13
2.1 Lay summary....15
3 Background, rationale and objectives....16
3.1 Background....16
3.2 Rationale....16
3.3 Objectives....18
4 Study design....19
4.1 Risk assessment....19
5 Participating site selection....19
6 Participant eligibility....21
6.1 Screening procedures....21
6.2 Inclusion criteria....22
6.3 Exclusion criteria....23
6.4 Informed consent....23
7 Randomisation....24
8 Trial treatments....25
8.1 Treatment dose and scheduling....25
8.2 Recommended drug administration schedule....26
8.3 Neo-adjuvant chemotherapy toxicity and dose modification....29
8.4 Chemoradiotherapy toxicity and dose modification....31
8.5 Calculating and recalculating BSA/doses....33
8.6 Dose capping....33
8.7 Dose-banding....33
8.8 Compliance....33
8.9 Concurrent Radiotherapy....33
8.10 Other medicinal treatments (i.e Non-Investigational Medicinal Products (NIMPS))....33
8.11 Drug interactions/potential drug interactions....34
8.12 Drug supply, accountability, labelling and disposal....34
9 Radiotherapy....35
9.1 Introduction to radiotherapy....35
9.2 Target volume definition (TVD)....35
9.2.1 Middle 1/3 tumours....35
9.2.2 Lower 1/3 tumours....36
9.2.3 Lower 1/3 and GOJ tumour 4D CT target definition protocol.....36
9.3 Organs at risk....36
9.4 Treatment Plan Optimisation....37
9.5 Dose limitations of Volumes of Interest (VOI) and of Organs at Risk (OAR)....37
9.6 Treatment Verification....37
9.7 The management of unscheduled gaps in radiotherapy treatment....38
9.8 Radiotherapy quality assurance....38
9.8.1 Pre-trial quality assurance....38
9.9 On-trial quality assurance....38
10 Surgery....39
10.1 The abdominal phase....39
10.2 Thoracic Phase....40
11 Trial assessments....40
11.1 Screening assessments....40
11.2 Baseline assessments....41
11.3 Assessments on treatment....41
11.4 Follow up assessments – 6 months and 12 months post surgery....42
11.5 Completion of CRFs....42
11.6 Schedule of trial assessments....43

This material is the property of the Wales Cancer Trials Unit. Do not disclose or use except as authorised.

12 Safety reporting and pharmacovigilance ... 44
12.1 Causality Assessments ... 45
12.2 Expectedness Assessments ... 45
12.3 SAE reporting ... 47
12.3.1 Participating Site Responsibilities ... 47
12.3.2 The WCTU responsibilities ... 47
12.4 SUSAR reporting ... 48
12.5 Safety Reports ... 48
12.6 Pregnancy reporting whilst participating in the NeoSCOPE trial ... 48
12.7 Flowchart for Serious Adverse Event reporting ... 50
13 Trial management ... 51
13.1 Trial committees and trial management ... 51
13.2 Monitoring ... 51
13.2.1 Site monitoring ... 51
13.2.2 Central monitoring and data queries ... 51
13.3 Participant Withdrawal ... 52
13.4 Lost to follow-up ... 52
13.5 Protocol/GCP non-compliance ... 53
13.6 The End of the Trial ... 53
13.7 Archiving ... 53
14 Statistical considerations ... 53
14.1 Randomisation ... 53
14.2 Outcome measures ... 53
14.3 Sample size calculation ... 53
14.4 Statistical analyses ... 54
14.5 Sub-group analyses ... 54
15 T-NeoSCOPE: Sample collection for future translational research ... 54
15.1 Processing tumour samples ... 55
15.2 Processing blood samples ... 55
16 Publication policy ... 56
17 Ethical and regulatory considerations, and Informed Consent ... 57
17.1 Ethical approval ... 57
17.2 Clinical Trial Authorisation (CTA) ... 57
17.3 Regulatory Considerations ... 57
17.4 Research Governance approval ... 57
17.5 Sponsorship ... 57
17.6 Indemnity ... 58
17.7 Data protection ... 58
17.8 Finance ... 58
18 References ... 59
19 Appendices ... 62
APPENDIX 1: A guide to performing causality assessments ... 62
APPENDIX 2: WHO Performance status ... 62
APPENDIX 3: Renal function - Cockroft & Gault formula ... 63
APPENDIX 4: Briefing for the NeoSCOPE trial pathologist ... 64
APPENDIX 5: Screening Investigations ... 74
APPENDIX 6: TNM classification for oesophageal cancer – comparison between the 6th and 7th edition ... 75

CONFIDENTIAL
This material is the property of the Wales Cancer Trials Unit. Do not disclose or use except as authorised.

Abbreviations and glossary

**5DFRC**	5'-deoxy-5-fluorocytidine
**5FU**	5-Fluorouracil
**ABPI**	Association of the British Pharmaceutical Industry
**AE**	Adverse event
**ALT**	Alanine Aminotransferase
**ALP**	Alkaline Phosphatase
**AR**	Adverse reaction
**ASR**	Annual Safety Report
**AST**	Aspartate Aminotransferase
**BSA**	Body Surface Area
**CAP**	Capecitabine
**CI**	Chief Investigator; trial investigator
**CPAS**	Chemotherapy and Pharmacy Advisory Service
**CRF**	Case Report Form
**CRM**	Circumferential Resection Margin
**CRUK**	Cancer Research UK
**CRT**	Chemoradiotherapy
**CT**	Computerised Tomography
**CTA**	Clinical Trial Authorisation
**CTCAE**	Common Terminology Criteria for Adverse Events
**CTIMP**	Clinical Trial of an Investigational Medicinal Product.<br>A clinical trial that is within the scope of the UK Medicines for Human Use (Clinical Trials) Regulations 2004.
**CTV**	Clinical target volume
**DVH**	Dose Volume Histogram
**ECG**	Electrocardiography
**EDTA**	Ethylenediaminetetraacetic acid
**ELNI**	Elective Lymph Node Irradiation
**EudraCT**	European Union Drug Regulatory Agency Clinical Trial
**EUS**	Endoscopic Ultrasound
**GCP**	Good Clinical Practice
**G-CSF**	Granulocyte-Colony Stimulating Factor
**GOJ**	Gastro-oesophageal Junction
**GP**	General Practitioner
**GTN**	Glyceryl trinitrate
**GTV**	Gross tumour volume
**H&E**	Hematoxylin and eosin

This material is the property of the Wales Cancer Trials Unit. Do not disclose or use except as authorised.

**IB**	Investigator's Brochure
**ICH**	International Conference on Harmonisation
**ICH-GCP**	International Conference on Harmonisation – Good Clinical Practice
**ICRU**	International Commission on Radiation Units and Measurements
**IDMC**	Independent Data Monitoring Committee
**IGRT**	Imaging Guided Radiation Therapy
**IHC**	Immunohistochemical
**IMP**	Investigational Medicinal Product<br><br>A pharmaceutical form of an active substance or placebo being tested or used in a clinical trial, including products already with a marketing authorisation, but used or assembled (formulated or packaged) in a way different to the authorised form, or when used for an unauthorised indication, or when used to gain more information about the authorised form.
**INR**	International Normalised Ratio
**ISR**	Investigator Safety Report
**ISF**	Investigator Site File
**ITV**	Internal Target Volume
**IV**	Intravenous Infusion
**LN**	Lymph Node
**LR**	Left-Right
**LFT**	Liver Function Tests
**LVEF**	Left Ventricular Ejection Fraction (LVEF)
**MDT**	Multidisciplinary Team
**MHRA**	Medicines and Healthcare products Regulatory Agency
**MREC**	Multi-centre Research Ethics Committee
**MUGA**	Multi Gated Acquisition
**NCRI**	National Cancer Research Institute
**NCRN**	National Cancer Research Network
**NeoSCOPE**	Neo-adjuvant Study of Chemoradiotherapy in OesoPhageal Cancer
**NER**	Nucleotide Excision Repair
**NHS**	National Health Service
**NHS IC**	National Health Service Information Centre
**NIHR CSP**	National Institute for Health Research Co-ordinated System for Gaining NHS Permission. This system defines and carries out checks that only need to be done once, and those that are required for each NHS location/organisation.
**NIMP**	Non-Investigational Medicinal Product<br><br>Medicinal products that are not the object of investigation (i.e. other than the tested product, placebo or active comparator) supplied to the patients participating in the trial and used in accordance with the protocol. E.g. background treatment, rescue medication.

**NSAIDs**	Non-Steroidal Anti-Inflammatory Drugs
**OAR**	Organs at risk
**OS**	Overall Survival
**Ox**	Oxaliplatin
**PAF**	Plan Assessment Form
**Patient**	A patient under care who may be eligible for the trial but has not yet consented to participate in any trial related activities.
**Participant**	An individual who has given written informed consent and is participating in trial related activities
**pCR**	Pathological Complete Response
**PET**	Positron emission tomography
**PI**	Principal Investigator; site Investigator
**PIS**	Participant Information Sheet
**PRV**	Planning organ at Risk Volume
**PTV**	Planning Target Volume
**QA**	Quality Assurance
**R0**	No Residual Disease
**R1**	Microscopic Residual Disease
**R&D**	Research and Development
**RCR**	College of Radiologists
**REC**	Research Ethics Committee
**RoL**	Region of Interest
**RPDG**	Radiotherapy Planning and Delivery Guideline
**RSI**	Reference Safety Information
**RT**	Radiotherapy
**RTTQA**	Radiotherapy Trials Quality Assurance
**SAE**	Serious Adverse Event
**SAR**	Serious Adverse Reaction
**SCRN**	Scottish Cancer Research Network
**SI**	Superior-Inferior
**SOP**	Standard Operating Procedure
**Sponsor**	The primary organisation that oversees and is responsible for the clinical trial
**SPC**	Summary of Product Characteristics
**SSA**	Site-Specific Assessment
**SUSAR**	Suspected Unexpected Serious Adverse Reaction
**SUV**	Standardized Uptake Value
**TMF**	Trial Master File
**TMG**	Trial Management Group

CONFIDENTIAL
This material is the property of the Wales Cancer Trials Unit. Do not disclose or use except as authorised.

**TPS**	Treatment Planning System
**TSC**	Trial Steering Committee
**TSF**	Trial Site File
**TVD**	Target Volume Definition
**UKCRC**	United Kingdom Clinical Research Collaboration
**VOI**	Volumes of interest
**WCB**	Wales Cancer Bank
**WCTU**	Wales Cancer Trials Unit

CONFIDENTIAL
This material is the property of the Wales Cancer Trials Unit. Do not disclose or use except as authorised.

1 Trial schema

- Histologically confirmed operable oesophageal cancer
- T3/ T4 with any N stage OR N1 with any T stage (TNM6). This will be equivalent to T3/T4a with any N stage OR N1-3 with any T stage (TNM7). Maximum disease (T+N) length 8 cm staged with EUS and CT/PET
- WHO performance status 0-1.

↓

RANDOMISATION

↓

Arm A

2 cycles OxCap:
Oxaliplatin 130mg/m^2 Day1
Capecitabine 625mg/m^2 bd Day 1- 21
then CRT:
Oxaliplatin 85mg/m^2 Days 1, 15, 29;
Capecitabine 625mg/m^2 bd only on days when receiving RT
XRT: 45 Gy in 25 fractions*

Arm B

2 cycles OxCap:
Oxaliplatin 130mg/m^2 Day 1
Capecitabine 625mg/m^2 bd Day 1- 21
then CRT:
Paclitaxel 50mg/m^2 Days 1,8,15,22,29;
Carboplatin AUC 2 Days 1,8,15,22,29
XRT: 45 Gy in 25 fractions*

↓

A CT scan of thorax, abdomen and pelvis will be performed at 4 weeks post CRT as per standard practice to exclude disease progression.
then:
Surgery (6-8 weeks post CRT)
then:
Clinical follow up at 6 week post surgery to assess in-hospital surgical morbidity/mortality.
Clinical follow up at 6 and 12 months post surgery to assess late toxicity.

ENDPOINTS
Primary:
Efficacy: Pathological complete response rate (pCR) to be assessed in patients undergoing resection following neo-adjuvant treatment, as measured using standardised histological interpretation.
Secondary:
Feasibility: of recruiting to a pre-operative CRT trial in the UK as determined by recruitment within 18 months.
Toxicity: SAEs collected in real time, 30 day surgical morbidity/mortality, toxicities (CTCAE version 4) during treatment and late treatment toxicity at 6 and 12 months.
Efficacy: CRM positivity at resection; median, 3 and 5 year overall survival.
* A detailed RT protocol and RTQA programme accompanies this protocol.

CONFIDENTIAL
This material is the property of the Wales Cancer Trials Unit. Do not disclose or use except as authorised.

2 Trial synopsis

Study title:	A randomised Phase II study of two pre-operative chemoradiotherapy regimens (oxaliplatin and capecitabine followed by radiotherapy with either oxaliplatin and capecitabine or paclitaxel and carboplatin) for resectable oesophageal cancer				
Study acronym:	NeoSCOPE				
Short title:	Neo-adjuvant Study of Chemoradiotherapy in OesoPhageal Cancer				
EudraCT No:	2012-000640-10				
Funder:	CR-UK	Funder's No:	C44694/A14614		
Chief Investigator:	Tom Crosby				
Sponsor:	Velindre NHS Trust				
Study period:	2 years	Phase:	II	Number of arms:	2
Number of participants:	85				
Investigational Medicinal Products(s) (IMP)	Oxaliplatin, capecitabine, paclitaxel, carboplatin				

Objective

To test the safety, efficacy, and feasibility of recruiting to a randomised multi-centre trial of pre-operative CRT in the UK and identify a safe and effective regimen that can be taken forward to a future Phase III trial where neo-adjuvant CRT will be compared with neo-adjuvant chemotherapy in patients with locally advanced resectable oesophageal cancer at high risk of R1 resection.

Main inclusion criteria:

- Histologically confirmed oesophageal cancer [adenocarcinoma] considered resectable with curative intent
- T3/ T4 with any N stage OR N1 with any T stage (TNM6) staged with endoscopic ultrasound (EUS) and CT/PET. This will be equivalent to T3/T4a with any N stage OR N1-3 with any T stage (TNM7).
- Maximum disease (T+N) length 8 cm measured by CT/PET/EUS
- WHO performance status 0-1
- Adequate haematological, renal, respiratory, cardiac and hepatic function
- The patient has provided written informed consent.

See Section 6.2 for full inclusion criteria.

Main exclusion criteria:

- Uncontrolled angina pectoris, myocardial infarction within 6 months, heart failure, clinically significant uncontrolled cardiac arrhythmias, or any patient with a clinically significant abnormal ECG.
- Patients with any previous treatment for oesophageal carcinoma.
- Siewert type 3 oesophago-gastric tumours.
- T4 tumours invading contiguous structures other than diaphragm, crura or mediastinal pleura.
- Patients with disease in any of the following areas on the CT scan, EUS or other staging investigation:
    - Evidence of other distant metastases.
    - Para-aortic lymphadenopathy >1cm diameter on CT or >6mm diameter on EUS.
    - Invasion of tracheo-bronchial tree, aorta, pericardium or lung.
- Lymphadenopathy encasing the coeliac axis (as described above, patients with single nodes lying anterior to the origin of the splenic artery and anterior to the origin of the coeliac axis are

CONFIDENTIAL
This material is the property of the Wales Cancer Trials Unit. Do not disclose or use except as authorised.

not excluded).

See Section 6.3 for full exclusion criteria.

Treatments:

All patients will receive 2 x 21 day cycles OxCap:

- Oxaliplatin 130mg/m^2 Day 1
- Capecitabine 625mg/m^2 bd Day 1—21

then CRT, either **Arm A:**

- **Oxaliplatin 85mg/m^2 Days 1,15,29;**
- **Capecitabine 625mg/m^2 bd only on days when receiving RT**
- XRT: 45 Gy in 25 fractions (weekends off)

or **Arm B:**

- **Paclitaxel 50mg/m^2 Days 1,8,15,22,29;**
- **Carboplatin AUC 2 Days 1,8,15,22,29**
- XRT: 45 Gy in 25 fractions (weekends off)

then Surgery (week 6-8 post CRT)

NB Local centres may choose to give weekly chemotherapy treatment starting Day 1 or 2 of radiotherapy e.g. to avoid Bank Holidays but should be given on the same day each week.

Trial assessments:

Screening assessments:

- Endoscopic assessment with biopsy
- Spiral/multi-slice CT
- Endoscopic ultrasound (EUS)
- FEV1 using a spirometer
- Cardiac ejection fraction using echocardiography or MUGA
- ECG
- Blood or pregnancy test where appropriate

Prior to each cycle during neoadjuvant chemotherapy and weekly during chemoradiotherapy:

- Clinical examination
- Haematology and biochemistry
- Assessment of NCI CTCAE according to version 4.03

End of chemoradiotherapy:

- Clinical examination
- Assessment of NCI CTCAE according to version 4.03

Pre surgery:

- Spiral/multi-slice CT with oral contrast or water. Maximum slice width 5mm. Intravenous infusion (IV) contrast/venous phase. CT must include abdomen and chest. Neck and pelvis fields are optional.
- Clinical examination
- Assessment of NCI CTCAE according to version 4.03

30 days post surgery:

- Clinical examination
- Postoperative complications
- Postoperative histology – report, slides and photographs to be sent for central review – see Appendix 4

Follow up assessments – 6 months and 12 months post surgery:

- Clinical examination
- Assessment of NCI CTCAE according to version 4.03

CONFIDENTIAL
This material is the property of the Wales Cancer Trials Unit. Do not disclose or use except as authorised.

For those patients who consent to the translational sample collection substudy:<br>• 2x10ml blood at baseline<br>• Paraffin blocks from the diagnostic biopsy
**Endpoints:**<br>***Primary:***<br>Efficacy: Pathological complete response rate (pCR) to be assessed in patients undergoing resection following neo-adjuvant treatment, as measured using a standardised histological interpretation and central pathology review.<br>***Secondary:***<br>Feasibility: of recruiting to a pre-operative CRT trial in the UK as determined by recruitment within 18 months.<br>Toxicity: SAEs collected in real time, 30 day surgical morbidity/mortality, toxicities (CTCAE version 4.03) during treatment and late treatment toxicity at 6 and 12 months.<br>Efficacy: CRM (circumferential resection margin) positivity at resection; median, 3 and 5 year survival

2.1 Lay summary

About 7500 patients are diagnosed with oesophageal cancer each year in the UK of which less than a quarter have resectable disease at diagnosis. There is a general lack of consistency in the standard of care for patients across UK hospitals. Patients are either treated with a) chemotherapy followed by surgical removal of the tumour, or b chemoradiotherapy followed by removal of the tumour by surgery, as part of their standard of care. Recent research supports the latter treatment, as chemoradiotherapy maybe more effective at shrinking the tumour and preventing the disease from spreading than taking chemotherapy alone. However, there is no definitive way of identifying which treatment is best without a clinical trial.

Evidence suggests that the effect of the chemoradiotherapy currently used as standard practice may be improved and the side effects reduced by using a different chemoradiotherapy combination. In this trial, eligible patients will receive 2 cycles of the same chemotherapy before being randomised to receive two different chemoradiotherapy regimens (carboplatin and paclitaxel verses oxaliplatin and capecitabine) both of which have shown promising results in previous studies. Patients will then have their tumour removed. The best chemoradiotherapy regimen will then be taken forward to a Phase III trial in which chemoradiotherapy will be compared with chemotherapy alone.

The efficacy of the regimens will be measured by counting the number of patients who i) remain free from cancer, ii) have local or distant spread of their cancer, iii) are successfully recruited and iv) experience toxicities. A specific set of toxicity criteria will be used to monitor any treatment induced side-effects and provide justification for any necessary dose modifications or withdrawal of treatment.

CONFIDENTIAL
This material is the property of the Wales Cancer Trials Unit. Do not disclose or use except as authorised.

3 Background, rationale and objectives

3.1 Background

In the UK, about 7500 patients are diagnosed with oesophageal cancer each year of which fewer than a quarter have resectable disease at diagnosis [CancerStats 2011]. The cornerstone of curative treatment in the UK remains surgery. Approximately 10-20% of all cases in the UK are treated by a surgical approach [Palser 2010]. The most common UK treatment strategy in the past 10 years has been neo-adjuvant chemotherapy in the form of 2 cycles of chemotherapy, comprising cisplatin and 5-fluorouracil [5FU], prior to surgery. This is based upon the results of the MRC OE02 trial which demonstrated a 6% survival benefit for this approach over surgery alone after 5 years. However, the overall survival is still poor, just 23% of patients surviving 5 years from treatment [Allum 2009]. The MRC OE05 trial which compared 4 cycles of neo-adjuvant ECX [epirubicin, cisplatin, capecitabine] chemotherapy compared to two cycles of cisplatin/5FU closed to recruitment in 2011.

The majority of patients with oesophageal cancer present with symptoms of locally advanced disease and most of those found to be suitable for surgery will have stage III disease (at least T3 with lymph node metastases). The oesophagus lacks a serosal surface and tumours frequently threaten the circumferential resection margin (CRM) of the surgical resection specimen. Disease present at or within 1mm of the circumferential resection margin (CRM) (R1 resection) occurs in more than 50% of stage III cases treated by surgery alone [Khan 2010, Dexter 2001] and is a poor prognostic factor. In the OE02 study, the 3-year and the median survival for patients with R0 and R1 resection were reported as 42.4% *vs.* 18% and 2.1 years *vs.* 1.1 years, respectively [Allum 2009]. Neo-adjuvant chemoradiotherapy [CRT] has become a standard management strategy in rectal cancer for patients who have a threatened CRM on pre-operative staging.

The use of neo-adjuvant CRT in oesophageal cancer has been tested in a number of studies which have been heterogeneous in design, size and treatment regimen tested. Nevertheless, a meta-analysis of randomised trials has shown that this approach increases R0 resection rates, reduces loco-regional recurrence and improves survival compared with surgery alone [Urschel 2003]. There has been only one randomised phase III trial comparing pre-operative chemotherapy with pre-operative CRT. This study by Stahl *et al* aimed to recruit 354 patients to detect a 10% improvement in 3-year overall survival [OS] in favour of CRT [from 25% to 35%] but had to close early as only 126 patients could be recruited in 5 years. Nonetheless, it showed a non-significant trend towards improved 3-year survival in favour of CRT [47.4% v 27.7%, p=0.07] [Stahl 2009].

Through better selection of patients, improved peri-operative care and centralisation of Upper GI surgical services, there has been a significant reduction in post-operative mortality. In the MRC OE02 trial, the post-operative mortality was 10% in patients receiving either neo-adjuvant chemotherapy or surgery alone [MRC Oesophageal Cancer Working Group 2002]. In the 3rd Annual Report of the UK National Oesophago-Gastric Cancer Audit (26th March 2012), the post-operative mortality was 3.8% and many large surgical centres now have rates of in-hospital mortality of < 3% [Palser 2010]. Neo-adjuvant CRT has been associated with higher post-operative mortality. In the Urschel meta-analysis, there was a non-significant increase in peri-operative mortality [1.72 (0.96, 3.07; p=0.07)] and increase in all-treatment mortality which was of borderline significance [1.63 (0.99, 2.68; p=0.053)] [Urschel 2003]. In the Stahl trial of neo-adjuvant CRT *vs.* neo-adjuvant CT, there was a trend towards increased post-operative mortality [5 of 49 (10.2%) *vs.* 2 of 52 (3.8%, p=0.26] [Stahl 2009]. Although only recently reported, this study was designed in the 1990s and opened to recruitment in November 2000. Three-dimensional conformal radiotherapy was recommended but not mandated as part of this trial. This, together with the lack of pre/on-trial RT quality assurance, may have contributed to increased post-operative morbidity.

3.2 Rationale

More recently, and not included in the meta-analysis above, a randomised phase III study reported in ASCO 2010 comparing surgery (S) alone to neo-adjuvant CRT (CRT-S), has shown a near doubling of OS in favour of the CRT-S arm [OS 49 *vs.* 26 months, HR 0.67], a pathological complete response [pCR] rate of 32%, and no increase in surgical

This material is the property of the Wales Cancer Trials Unit. Do not disclose or use except as authorised.

mortality [3.8% (S) *vs.* 3.4% (CRT-S)] [Van Hagen 2012]. In this trial, 368 patients with operable oesophageal or gastro-oesophageal junction tumours were randomised to surgery alone or to a neo-adjuvant CRT regimen of weekly carboplatin [AUC 2] and paclitaxel [50mg/m2] concurrent with radiotherapy [41.4 Gy in 23 fractions]. Of the 180 patients assigned to the CRT-S arm, 163 completed protocol treatment and the study reported a low incidence of Grade 3/4 CRT toxicity [haematological 6.8%; non-haematological 16%]. The R0 resection rates in the S and CRT-S arms were 69% and 92%, respectively [p=0.002]. The results of this study, performed in patients with a similar stage and tumour morphology to those in the UK, would suggest that where neo-adjuvant CRT is delivered safely, this may lead to a significant improvement in outcome. This approach, both in terms of treatment strategy and regimen, warrants further evaluation.

Oxaliplatin has now been shown to be at least equivalent to cisplatin in advanced upper GI cancers, can be given as a convenient 2 hour infusion and has a more favourable toxicity profile compared to cisplatin [REAL 2 study]. Oxaliplatin based CRT has been tested in Phase II trials, both in the neo-adjuvant and definitive treatment of oesophageal cancer [Javle 2009, Khushalani 2002, Chiarion-Sileni 2009, Wahba 2011, O'Connor 2007, Thukral 2010, Conroy 2010]. In summary, studies of oxaliplatin–based CRT show promising activity with acceptable toxicity and justify further testing in this study proposal.

As with other cancers, it is hoped that understanding the mechanisms of chemoradiotherapy resistance may enable us to individualise our approach to gastro-oesophageal cancer therapy in the near future. The nucleotide excision repair pathway [NER] repairs platinum-DNA adducts and tumours with high levels of NER endonucleases (ERCC1 and XPF) have been shown to be associated with platinum-resistance in gastro-oesophageal cancer [Tanaka 2009]. In a recently concluded study in Oxford evaluating oxaliplatin-5FU neo-adjuvant chemotherapy in 50 patients with resectable oesophageal carcinoma, tumours expressing low levels of XPF in the pre-treatment biopsy were found to have a significantly higher chance of responding to platinum chemotherapy compared to those with high XPF expression [64% *vs.* 33%] [Sharma personal comm.]. More recently, a phase II study evaluating the role of oxaliplatin-FU based neo-adjuvant CRT with prospective exploratory analysis of intra-tumoural mRNA expression and polymorphism in genes involved in drug metabolism has been reported [Leichmann, JCO Dec 2011]. A total of 98 patients were entered into the study, 93 of whom were evaluable for response, toxicity and survival. With a post-operative mortality of 2.2% and a pCR rate of 29%, this study has demonstrated that neo-adjuvant oxaliplatin-fluoropyrimidine based CRT is both tolerable and has promising efficacy. At a median follow up of 39.2 months, the median and 3-year OS were 28.3 months and 45.1%, respectively. Pre-treatment ERCC1 gene expression was found to be inversely related to OS [HR 2.72, p=0.015] and PFS [HR 0.77, p=0.007]. The potential to predict platinum resistance together with the encouraging results with taxane based CRT supports the testing of more than one CRT regimen, containing both, platinum agents and a taxane.

In recent years, a great deal of work has been done to raise the standards of conformal radiotherapy for oesophageal cancer in the UK. Through a detailed protocol and quality assurance procedures, including test cases and on trial review of plans, the SCOPE 1 trial (CI: Tom Crosby, a randomised trial of definitive CRT with cisplatin and capecitabine with/without Cetuximab) recruited > 250 patients from 44 radiotherapy centres and demonstrated that the UK can perform high quality, multi-centre RT based studies safely (Hurt 2011). This trial has allowed UK investigators to gain significant experience in delivering quality assured CRT and provides an ideal platform on which to build a neo-adjuvant study, and to investigate precise methods of tumour localisation such as CT/PET and image-guided radiotherapy [IGRT], all of which should allow safer and more accurate delivery of RT, reduction of radiation damage to organs at risk and ultimately reduce post-operative morbidity and mortality.

In summary, the current UK standard practice has been largely influenced by the results of the MRC OE02 trial and until recently, clinicians had been actively recruiting to the MRC OE05 trial. Concerns about increased post-operative mortality/morbidity from neo-adjuvant CRT as well as participation in the MRC OE05 trial had discouraged routine use of neo-adjuvant CRT. However, the MRC OE05 trial has now closed to recruitment, the outcome with neo-adjuvant chemotherapy alone remains poor and the UK has now seen the development of a high quality upper GI RT Quality Assurance programme through the SCOPE 1 trial. This, along with improvement in peri-operative care, is expected to lead to better outcome than reported in previous neo-adjuvant CRT trials. The CROSS trial demonstrated that neo-adjuvant CRT can be given with acceptable morbidity, and when done so, is associated with a significant survival advantage [Van Hagen 2012].

Based on the above, the provocative results from the Stahl trial and non comparative meta-analyses, there is a growing clinical consensus that the two strategies of neo-adjuvant chemotherapy and CRT should be compared head-to-head in a prospective randomised controlled trial, particularly focussing on those patients who are at high risk of R1 surgical resection. To do this, it is important that we establish the efficacy, safety and feasibility of neo-adjuvant CRT in the UK.

3.3 Objectives

NeoSCOPE is a randomised phase II study which will test the safety (with regard to post-operative morbidity/mortality), efficacy (determined by pathological complete response in the resected specimen, pCR) and feasibility of recruiting to a randomised multi-centre trial of neo-adjuvant CRT in the UK.

The randomised phase II design allows us to test two differing radiosensitiser schedules [carboplatin/paclitaxel and oxaliplatin/capecitabine] with non-overlapping toxicities. The study is aimed to identify a safe and effective regimen that can be taken forward to a future Phase III trial where neo-adjuvant CRT will be compared with neo-adjuvant chemotherapy in patients with locally advanced oesophageal cancer at high risk of R1 disease at surgery.

Subsidiary questions: As well as demonstrating that this treatment can be delivered safely and selecting the best regimen to be taken forward, this study provides an ideal opportunity to standardise and improve the separate components of pre-operative CRT prior to use in a Phase III trial setting.

1. Does PET/CT improve the accuracy and reproducibility of target volume delineation?

The aim of the substudy is to explore the impact of PET on the accuracy and reproducibility of target volume definition (TVD) in oesophageal cancer, linking in with a 3 year project on the optimisation of PET based TVD in head and neck cancer (POSITIVE study – PI: E Spezi, Velindre Cancer Centre). A retrospective central review of the planned gross tumour volume (GTV) will be undertaken, comparing these to a solely PET defined GTV (based on the diagnostic PET image). There is currently no consensus on the optimal method of contouring GTV on PET for oesophageal cancer [Macmanus 2009, Muijs 2010]. PET/CT and planning CT scans will be centrally retrieved and fused with image fusion technology featuring deformable image registration algorithms. Established volume defining algorithms such as thresholding, continuous standardized uptake value (SUV) boundaries, region growing and second differentials will be investigated alongside novel volume defining algorithms developed as part of the POSITIVE study. A comparison of autocontouring with manual delineation based on these algorithms will examine the impact on interobserver variation.

2. Does Image Guided Radiotherapy (IGRT) improve the therapeutic index in oesophageal cancer?

It is recognized that respiration has an impact on primary oesophageal tumour and lymph node movement, and needs to be incorporated into modern radiation therapy planning protocols [Martinger 2009]. Studies have consistently shown that it is the lower third oesophageal tumours and subdiaphragmatic lymph nodes that have the largest amplitude of movement [Yamashita 2011]. The feasibility of using 4D CT scans to evaluate the movement of these tumours and refine tumour target volume definition in lower third oesophageal cancers will be assessed. This study will also use 3D volumetric treatment verification using linac based X ray volumetric Cone beam CT imaging to investigate and guide further improvement in treatment efficacy [Hawkins 2010] for oesophageal cancer.

Additionally, samples for future translational research will be collected. See section 15.

CONFIDENTIAL
This material is the property of the Wales Cancer Trials Unit. Do not disclose or use except as authorised.

4 Study design

NeoSCOPE is designed as a single stage, non-blinded, randomised, "pick a winner" Phase II study of two novel neo-adjuvant chemoradiotherapy regimens for resectable oesophageal cancer. A randomised "pick a winner" design has been selected as we are intending to select one of the two experimental regimens to take forward to a Phase III where it will be compared against standard care.

4.1 Risk assessment

A Trial Risk Assessment has been completed by the WCTU to identify the potential hazards associated with the trial and to assess the likelihood of those hazards occurring and resulting in harm. This risk assessment has been completed in accordance with the MRC/DH/MHRA Joint project guidance document 'Risk-adapted approaches to the management of Clinical Trials of Investigational Medicinal Products' and includes:

- The risk to participant safety in relation to the IMP
- All other risks related to the design and methods of the trial (including risks to participant safety and rights as well as reliability of results)

The potential risks have been balanced against the level of risk that a trial participant would be exposed to outside of the trial. This trial has been categorised as a TYPE B where the level of risk is somewhat higher than the risk of standard medical care. This category was used to determine the level of monitoring in section 13.2. A copy of the trial risk assessment may be requested from the WCTU Trial Manager.

5 Participating site selection

This study will be carried out at participating sites within the UK. All sites who are interested in participating in the trial will be required to complete a registration form to confirm that they have adequate resources and experience to conduct the trial.

The following documentation must be completed and received by the WCTU in order for a site to begin recruitment:

1. Confirmation of local R&D approval
2. Favourable opinion of host care organisation/Principal Investigator (PI) from Main Ethics committee
3. Signed partnership agreement between the host care organisation and Sponsor
4. Current Curriculum Vitae and GCP training certificate of the PI
5. A copy of the most recent approved version of the Participant Information Sheet(s) (PIS), Consent Form(s), and Withdrawal Form(s) on host care organisation headed paper
6. A copy of the most recent approved GP letter on host care organisation headed paper
7. A copy of the most recent Pregnancy Information Sheet(s) and Consent Form(s) on host care organisation headed paper
8. Completed Delegation Log (signature list and delegation of responsibilities)
9. Full contact details for all host care organisation personnel, indicating preferred contact
10. A set of laboratory normal ranges and laboratory certification/accreditation from the host care organisation laboratory being used for analyses
11. Appropriate arrangements between the participating site and any site conducting trial treatments outside of the participating Trust (if applicable). This would include the completion of schedule 4 of the Sponsor-site agreement
12. Pass of quality assurance (QA) test for radiotherapy planning and treatment delivery (see radiotherapy quality assurance (RTQA) in section 9.6)

CONFIDENTIAL
This material is the property of the Wales Cancer Trials Unit. Do not disclose or use except as authorised.

13. PI to confirm that an appropriately specialised multidisciplinary team (MDT) is in place:
    a. Participating surgeons should have experience with two phase oesophagectomy and two-field lymphadenectomy (it is recommended that un-proctored surgeons should have performed a minimum of 12 such operations prior to commencement of the trial)
    b. The clinical trial centre has histopathologists who are experienced in the reporting of oesophageal cancer and who agree to adhere to the NeoSCOPE pathology protocol for the resection specimen (Appendix 3). Centres agree to support the NeoSCOPE central pathology review and the T-NeoSCOPE study (tissue sample collection)
14. Confirmation from the PI that he/she has discussed the study requirements regarding the resection specimen with the surgeon and with the pathology department and that both confirm that they will comply/adhere to the protocol.

Once all the documentation has been received at the WCTU, confirmation of site approval will be sent by the WCTU to the site PI.

All documentation must be stored in the Investigator Site File (ISF) at the site and in the Trial Site File (TSF) at the WCTU. The WCTU must be notified of any changes to the trial personnel and their responsibilities during the running of the trial and the respective trial files must contain this up-to-date information.

Site initiation will be by attendance at a NeoSCOPE launch meeting or by teleconference if attendance of key personnel is unfeasible.

Occasionally during the trial, amendments may be made to the trial documentation listed above. WCTU will issue the site with the latest version of the documents as soon as they become available. It is the responsibility of the site to ensure that they obtain local R&D approval for the new documents, and that all relevant staff, including pharmacy staff, are working to the current versions once R&D approval has been obtained.

CONFIDENTIAL
This material is the property of the Wales Cancer Trials Unit. Do not disclose or use except as authorised.

6 Participant eligibility

Any queries about whether a patient is eligible to enter the trial should be discussed with the WCTU before randomisation. Any issues will then be raised with the Chief Investigator (CI) or one of the clinical Co-Investigators in the CI's absence.

Patients are eligible for the trial if all the inclusion criteria (Section 6.2) are met and none of the exclusion criteria (Section 6.3) apply.

The PI must confirm the eligibility of a patient in the patient's medical notes prior to randomisation.

6.1 Screening procedures

Before any trial specific procedures are undertaken, the patient's written informed consent must be obtained. The patient should be given a minimum of 24 hours after initial invitation to participate before being asked to sign the consent form. Any procedures that have already been done before the patient was considered for the trial can be used to assess eligibility and do not need to be repeated as long as they were conducted in the timelines stipulated below. Patients must have histologically confirmed carcinoma of the oesophagus. This will have been determined by endoscopic assessment with biopsy.

Tumour Staging Assessments

Each of the tumour staging investigations outlined below should be aimed at being performed within 4 weeks prior to randomisation. However if this is not possible, the last staging investigation, which may include CT, EUS, PET/CT, or laparoscopy, should normally be performed within 4 weeks prior to randomisation. If the last staging investigation is > 4 weeks please contact the CI via the NeoSCOPE Trial Manager.

1. Spiral/multi-slice CT with oral contrast or water. Maximum slice width 5mm. IV contrast/venous phase. CT must include abdomen and chest. Neck and pelvis fields are optional.
2. EUS performed using a radial scanner. For obstructing tumours, the use of an oesophago-probe is preferable, but dilatation to facilitate complete scanning is permissible. EUS should include recording of proximal and distal extent of primary tumour, location of lymphadenopathy and reference point for localisation on CT planning (Appendix 5).
3. Laparoscopy where clinically indicated at discretion of treating physician.
4. Bone scans are optional and should be performed according to local practice.
5. PET scans are recommended. **If a patient has consented to the NeoSCOPE study and has been randomised into the trial, please send a CD-ROM of the staging CT/PET scan to:**
   NeoSCOPE Trial Manager
   Wales Cancer Trials Unit
   6th Floor
   Neuadd Meirionnydd
   Heath Park
   Cardiff CF14 4YS
   The CD-ROM should be labelled with the patient's trial number, date of birth and initials but should be otherwise anonymised.

More detailed information on how investigations such as the CT scan and endoscopic ultrasound should be performed are given in Appendix 5. These are recommendations and should not replace local guidelines.

Please note that EUS should be used to stage local disease (tumour and nodes) and that these results should overrule results from CT. CT should be used to stage distant spread. CT/PET, where performed, should complement these 2 investigations.

CONFIDENTIAL
This material is the property of the Wales Cancer Trials Unit. Do not disclose or use except as authorised.

Cardiac, Respiratory, Hepatic, Renal and Haematological Function Assessments

Normally *within 4 weeks* of randomisation (see inclusion/exclusion criteria below for more detail)

- FEV1 using a spirometer
- Cardiac ejection fraction using echocardiography or MUGA
- ECG

Normally *within 1 week* (see inclusion/exclusion criteria below for more detail):

- Blood test for hepatic, renal and haematological function
- Pregnancy test in females of child bearing age

6.2 Inclusion criteria

Patients meeting any of the following criteria may be included in the trial:

1. Histologically confirmed operable oesophageal cancer [adenocarcinoma]

2. T3/ T4 with any N stage OR N1 with any T stage (TNM6). This will be equivalent to T3/T4a with any N stage OR N1-3 with any T stage (TNM7). T4a tumours should;
   a. involve only the diaphragm or crura, or
   b. invade only the mediastinal pleura, or
   c. breach the gastric serosa (TNM 7).

   Tumours with nodal disease (N1-3) affecting the origin of the left gastric and splenic artery with the coeliac axis (formerly staged as M1a in TNM 6) can be included. A comparative summary between the TNM 6th and the more current TNM 7th edition is provided in Appendix 6.

3. Maximum disease (T+N) length 8 cm staged with EUS and CT/PET with maximum extent of primary disease below the gastro-oesophageal junction being 3cm.

4. WHO performance status 0-1 (Appendix 2) and patient fit to be treated with combined modality therapy (chemotherapy and radiotherapy prior to surgery).

5. Adequate respiratory and cardiac function: FEV1 >1.5 litres and cardiac ejection fraction ≥50% on echocardiography or MUGA. These assessments should normally be performed within 4 weeks prior to randomisation. CPEX testing is allowable but must not replace the above investigations. Patients who have had their assessments done over 4 weeks prior to randomisation or have had borderline results may still be eligible provided that they have approval from the CI through the NeoSCOPE trial team.

6. Adequate haematological, renal, and hepatic function :
   a. liver function tests ≤1.5 x ULN
   b. white blood cell count ≥ 3 x 10^9/l; platelets ≥ 100 x 10^9/l.
   c. glomerular filtration rate (GFR) >50ml/minute calculated or measured (see Appendix 3).

   The above assessments should normally be performed within 1 week prior to randomisation. Patients who have had their assessments done over 1 week prior to randomisation or have had borderline results may still be eligible provided that they have approval from the CI through the trial team.

7. The patient has provided written informed consent.

8. The patient is at least 18 years old.

CONFIDENTIAL
This material is the property of the Wales Cancer Trials Unit. Do not disclose or use except as authorised.

6.3 Exclusion criteria

If any of the following criteria apply, patients cannot be included in the trial:

1. Oesophageal cancer with histology other than adenocarcinoma
2. Uncontrolled angina pectoris, myocardial infarction within 6 months, heart failure, clinically significant uncontrolled cardiac arrhythmias, or any patient with a clinically significant abnormal ECG.
3. Patients with any previous treatment for oesophageal carcinoma.
4. Siewert type 3 oesophago-gastric tumours.
5. Lower limit of the endoscopically visible primary tumour should not involve stomach for more than 3cm distal to the gastro-oesophageal junction.
6. T4 tumours invading contiguous structures other than diaphragm, crura or mediastinal pleura.
7. Patients with disease in any of the following areas on the CT scan, EUS or other staging investigation:
   a. Evidence of metastases in liver, lung, bone or other distant metastases.
   b. Abdominal para aortic lymphadenopathy >1cm diameter on CT or >6mm diameter on EUS.
   c. Invasion of tracheo-bronchial tree, aorta, pericardium or lung.
8. Lymphadenopathy encasing the coeliac axis (as described above, patients with single nodes lying anterior to the origin of the splenic artery and anterior to the origin of the coeliac axis are not excluded).
9. Any patient with a single significant medical condition which is thought likely to compromise his or her ability to tolerate any of the above therapies.
10. Specific contra-indications to surgery, chemotherapeutic agents (including known allergies to chemotherapy) or radiotherapy.
11. Patients with another previous or current malignant disease which in the judgement of the treating investigator is likely to interfere with treatment or the assessment of response.
12. Pregnant or lactating women and fertile women who will not be using contraception during the trial.

6.4 Informed consent

The patient's written informed consent must be obtained using the NeoSCOPE trial Consent Form, which follows the Patient Information Sheet (PIS). The patient should be given a minimum of 24 hours after the initial invitation to participate before being asked to sign the Consent Form by a trained member of staff on the delegation log. Please note, only when written informed consent has been obtained from the patients and they have been randomised into the trial can they be considered a trial participant.

Patients will also be asked to consent to NHS Information Centre Flagging so that the date and cause of death can be collected without longer term follow-up. This will be optional and additional to the standard informed consent.

The patient's consent to participate in the trial should be obtained after a full explanation has been given of the treatment options, including the conventional and generally accepted methods of treatment. All patients must be informed of the aims of the study, the possible adverse events, the procedures and possible hazards to which they may be exposed. They will be informed of the strict confidentiality of their patient data, but that their medical records may be reviewed for trial purposes by authorised individuals other than their treating physician.

Patient's consent will be sought to notify their general practitioner (GP) of their involvement in the trial. Patients should be given a minimum of 24 hours after being given the trial PIS to consider and discuss participation in the trial with

CONFIDENTIAL
This material is the property of the Wales Cancer Trials Unit. Do not disclose or use except as authorised.

friends and family. A contact number for someone at the site should be given to the patient should they wish to discuss any aspect of the trial. Following this, the randomising investigator should determine that the patient is fully informed of the trial and their participation, in accordance with the principles of GCP. Patients should always be asked to sign a consent form. One copy should be given to the participant but the original copy should be kept in the investigator site file and a further copy should be kept with the participant's hospital notes.

Participant consent is requested to collect NHS Numbers to utilise NHS data for future research, through Cancer Research UK and the National Cancer Intelligence Network (NCIN).

The right of the participant to refuse to participate in the trial without giving reasons must be respected. After the patient has entered the trial, the investigator must remain free to give alternative treatment to that specified in the protocol, at any stage, if he/she feels it to be in the best interest of the participant. However, the reason for doing so should be recorded and the participant will remain within the trial for the purpose of follow up and data analysis according to the treatment option to which he/she has been allocated. Similarly, the participant must remain free to withdraw at any time from the protocol treatment without giving reasons and without prejudicing his/her further treatment.

This is a randomised controlled trial, therefore neither the participants nor their physicians will be able to choose the participant's treatment. Treatment will be allocated randomly using a computer-based algorithm. This is to ensure that the groups of participants receiving each of the different treatments are similar.

All patients, including eligible and ineligible patients, should be recorded on the NeoSCOPE screening logs which should be faxed to WCTU on a quarterly basis.

7 Randomisation

Participant randomisation will be performed centrally by the WCTU. Randomisation can only be performed once the participant has signed the consent form. The randomisation form should be completed and the WCTU contacted on the following telephone number:

WCTU Randomisation line:
029 2064 5500
(Open Monday – Friday, 9am – 5pm)

N.B. This telephone number is strictly for randomisation and should not be used for general queries.

Participants will be randomised to a trial arm using the method of minimisation with a random element.

At randomisation, the participant will be given a unique participant trial number and the treatment allocation. These details should be recorded on the participant enrolment form and the top copy returned to the WCTU within 4 weeks.

After randomisation, the WCTU will fax confirmation to the Research Nurse and Pharmacist at the participating site. The participant's GP will be informed by the Site of the participant's enrolment, if the participant gives consent to do so.

It may be possible for participants to be recruited into other clinical trials, but this should be discussed with the CI via the WCTU before this is considered.

CONFIDENTIAL
This material is the property of the Wales Cancer Trials Unit. Do not disclose or use except as authorised.

8 Trial treatments

The following study drugs are classed as IMPs and are not licensed for use in neo-adjuvant treatment of oesophageal cancer:

- Capecitabine (Cap): UK marketing authorisation of the first line treatment of advanced gastric cancer in combination with a platinum-based agent. This drug is not licensed for use in neo-adjuvant treatment of oesophageal cancer.
- Oxaliplatin (Ox): UK marketing authorisation and licence for use in treatment of adjuvant and metastatic colorectal cancer. This drug is not licensed for use in neo-adjuvant treatment of oesophageal cancer.
- Paclitaxel: UK marketing authorisation of the first line treatment of advanced ovarian cancer in combination with a platinum-based agent. This drug is not licensed for use in neo-adjuvant treatment of oesophageal cancer
- Carboplatin: UK marketing authorisation of the first line treatment of advanced ovarian cancer. This drug is not licensed for use in neo-adjuvant treatment of oesophageal cancer.

These drugs should be supplied from a sites' own stock and provider – see the SmPCs at http://www.medicines.org.uk/emc/ for more information on toxicities, stability and administration.

Local hospital policy may be followed with respect to the diluents in which the IMPs are prepared, stability pre and post anti-emetics. Refer to www.medicines.org.uk

8.1 Treatment dose and scheduling

Patient should start chemotherapy treatment within 3 weeks of randomisation. Patients on either arm will receive 2 x21 day cycles of OxCap induction neo-adjuvant chemotherapy. Patients will then proceed immediately to chemoradiotherapy. Surgery will be performed within 6-8 weeks following completion of neo-adjuvant treatment subject to satisfactory re-staging investigations. No chemotherapy is planned post-operatively. Additional information on volume and diluent available in section 8.2.

OxCap induction neo-adjuvant chemotherapy: 2 cycles for all patients:

Oxaliplatin 130mg/m^2 IV infusion Day 1
Capecitabine 625mg/m^2 bd Days 1- 21

Chemoradiotherapy:

Arm A:

Oxaliplatin 85mg/m^2 IV infusion Day 1, 15, 29
Capecitabine 625mg/m^2 bd PO on days receiving RT (Total: 25 days of treatment)

**Week**	**1**							**2**							**3**							**4**							**5**						
**Days**	1	2	3	4	5	6	7	8	9	10	11	12	13	14	15	16	17	18	19	20	21	22	23	24	25	26	27	28	29	30	31	32	33	34	35
**Radiotherapy:** 45Gy / 25#	•	•	•	•	•			•	•	•	•	•			•	•	•	•	•			•	•	•	•	•			•	•	•	•	•		
**Oxaliplatin** 85mg/m^2	o														o														o						
**Oral capecitabine**	c	c	c	c	c			c	c	c	c	c			c	c	c	c	c			c	c	c	c	c			c	c	c	c	c		
625mg/m^2 orally bd Mon-Fri x 5 weeks	c	c	c	c	c			c	c	c	c	c			c	c	c	c	c			c	c	c	c	c			c	c	c	c	c		

Arm B:

Paclitaxel 50mg/m^2 IV infusion Day 1, 8, 15, 22, 29
Carboplatin AUC 2 IV infusion Day 1, 8, 15, 22, 29

Week	1							2							3							4							5						
**Days**	1	2	3	4	5	6	7	8	9	10	11	12	13	14	15	16	17	18	19	20	21	22	23	24	25	26	27	28	29	30	31	32	33	34	35
**Radiotherapy:** 45Gy / 25#	•	•	•	•	•			•	•	•	•	•			•	•	•	•	•			•	•	•	•	•			•	•	•	•	•		
**Paclitaxel 50mg/m^2**	P							P							p							P							p						
**Carboplatin AUC 2**	C							C							C							C							C						

Local centres may choose to give weekly chemotherapy treatment starting Day 1 or 2 of radiotherapy e.g. to avoid Bank Holidays but should be given on the same day each week (+/- one day).

8.2 Recommended drug administration schedule

Induction neo-adjuvant chemotherapy

Oxaliplatin:

Time			
T=minus 30mins	Pre-meds	Anti-emetics: Either IV or Oral<br>Ondansetron 8mg<br>Dexamethasone 8mg<br>Metoclopramide 20mg	Flush line with glucose 5% (not required if minibag used to administer IV anti-emetics)
T=0-2	Oxaliplatin 130mg/m^2	In 250ml to 500ml glucose 5%<br>(The final concentration of oxaliplatin must be between 0.2mg/ml and 0.7 mg/ml)	IV infusion over 2 hours
	Flush	Flush line through with glucose 5% using hospital approved pump	

Management of drug hypersensitivity and extravasation will be as per local hospital policy. Use of routine anti-emetics pre and post IV chemotherapy will also be as per local hospital policy. The above table is a recommendation only.

CONFIDENTIAL
This material is the property of the Wales Cancer Trials Unit. Do not disclose or use except as authorised.

Capecitabine:

Patients will be instructed to take the drug twice a day [to the nearest achievable dose using hospital stock capecitabine tablets]. Patients should take the tablets at approximately the same time each day, 12 hours apart [example 8:00am or 9:00am and 8:00pm or 9:00 pm respectively], within 30 minutes after eating. For patients with swallowing difficulties, capecitabine tablets can be dissolved by stirring for approximately 15 minutes in 200mls of lukewarm water. The solution should be swallowed immediately. The solution could be flavoured with a fruit juice or squash, but grapefruit juice should not be used. The solution may be administered through a feeding tube, which should be subsequently flushed to ensure that the full dose has been delivered. Please note the dissolving of capecitabine tablets for administration is outside of its licensed indication.

Missed doses will not be made up. The next doses must be taken as scheduled. The 'missed' tablets should be brought at next clinic visit to be handed over to the research nurse.

If a patient vomits after taking the tablets, they should not take another dose. The next doses should be taken as scheduled.

Any unused tablets should be returned to pharmacy (via research nurse). All patients are asked to keep a record of their capecitabine use in their diary cards. Please refer to section 8.8 of the protocol for capecitabine compliance.

Arm A chemoradiation schedule

Oxaliplatin:

Time			
T=minus 30mins	Pre-meds	Anti-emetics: Either IV or Oral<br>Ondansetron 8mg<br>Dexamethasone 8mg<br>Metoclopramide 20mg	Flush line with glucose 5% (not required if minibag used to administer IV anti-emetics)
T=0-2 hours	Oxaliplatin 85mg/m^2	In 250ml to 500ml glucose 5%<br>(The final concentration of oxaliplatin must be between 0.2mg/ml and 0.7 mg/ml)	IV infusion over 2 hours
	Flush	Flush line through with glucose 5% using hospital approved pump	

Management of drug hypersensitivity and extravasation will be as per local hospital policy.
Use of routine anti-emetics pre and post IV chemotherapy will be as per local hospital policy. The above table is a recommendation only.

Capecitabine:
Patients will be instructed to take the drug twice a day [to the nearest achievable dose using hospital stock capecitabine tablets]. Patients should take the tablets at approximately the same time each day, approximately 12 hours apart [example 8:00am or 9:00am and 8:00pm or 9:00 pm respectively], within 30 minutes after eating. For patients with swallowing difficulties, capecitabine tablets can be dissolved by stirring for approximately 15 mins in 200mls of lukewarm water. The solution should be swallowed immediately. The solution could be flavoured with a fruit juice or squash, but grapefruit juice should not be used. The solution may be administered through a feeding tube, which should be subsequently flushed to ensure that the full dose has been delivered. Please note the dissolving of capecitabine tablets for administration is outside of its licensed indication.

Missed doses will not be made up. The next doses must be taken as scheduled. The 'missed' tablets should be brought at next clinic visit to be handed over to the research nurse.
If a patient vomits after taking the tablets, they should not take another dose. The next doses should be taken as scheduled.

CONFIDENTIAL
This material is the property of the Wales Cancer Trials Unit. Do not disclose or use except as authorised.

Any unused tablets should be returned to pharmacy (via research nurse). All patients are asked to keep a record of their capecitabine use in their diary cards. Please refer to section 8.8 of the protocol for capecitabine compliance.

Arm B chemoradiation schedule

Paclitaxel and Carboplatin:

Time			
T=minus 30mins	Pre-meds	Dexamethasone 8mg IV and Ondansetron 8mg IV in 50ml 0.9% sodium chloride over 15 minutes<br>Chlorphenamine 10mg IV Bolus over 1 minute<br>Ranitidine 50mg IV Bolus over 2 minutes	
T=0 – 60 mins	Paclitaxel $50mg/m^2$	In 250ml sodium chloride 0.9%	IV infusion over 60 mins Administer paclitaxel through an in-line filter with a microporous membrane of < or = 0.22 microns
T = 60 -90 mins	Carboplatin AUC2	In 500mls glucose5%	IV infusion over 30mins
	Flush	Flush line through with glucose 5% using hospital approved pump	

- Calculate carboplatin dose using the Calvert equation: Dose (mg) = AUC x (GFR + 25)
- GFR used in the Calvert formula for carboplatin dosing should not exceed 125ml/min
- Use AUC of 2 for both calculated and measured GFR [maximum dose of carboplatin not to exceed 300mg for patients randomised onto the carboplatin and paclitaxel arm of the study]
- Re-calculate GFR if creatinine increases by ≥ 25%
- Use Cockroft Gault to calculate GFR ml/min:

GFR for males = $\frac{1.23 \times [140\text{-age}] \times \text{weight (kg)}}{\text{Serum creatinine (mol/l)}}$

GFR for females = $\frac{1.05 \times [140\text{-age}] \times \text{weight (kg)}}{\text{Serum creatinine (mol/l)}}$

- Management of drug hypersensitivity and extravasation will be as per local hospital policy.
  Use of routine anti-emetics pre and post IV chemotherapy will be as per local hospital policy. The above table is a recommendation only.

Actual versus ideal body weight

The dose of all chemotherapy drugs will be calculated for each patient based on actual weight. The BSA should be capped at $2m^2$.

8.3 Neo-adjuvant chemotherapy toxicity and dose modification

Common toxicities of oxaliplatin include: peripheral predominantly sensory neuropathy [often cold-related], nausea/vomiting, diarrhoea, bone-marrow suppression, immunogenic/ allergic reactions, stomatitis/mucositis. Detailed information can be found in the SPC available from www.medicines.org.uk.

Common toxicities of capecitabine include: plantar/palmar erythema, nausea and vomiting, diarrhoea, stomatitis, myelosuppression. Detailed information can be found in the SPC available from www.medicines.org.uk.

Haematological toxicity:

**Neutrophil count on day 22**		**Platelet count on day 22**	**Action**
≥1.5	AND	≥75	Full dose oxaliplatin and capecitabine
<1.5	OR	≥25 - <75	Stop capecitabine and delay oxaliplatin until recovery. Restart capecitabine at full dose. Oxaliplatin dose reduced to 75% for cycle 2
ANY	AND	<25	Stop capecitabine and delay oxaliplatin till recovery. Restart capecitabine at full dose. Oxaliplatin dose reduced to 50% for cycle 2

Neurotoxicity (this is commonly related to oxaliplatin):

Toxicity	***Cold-induced dysaesthesia only***	***Grade 1 or 2 (if Grade 2, persists <7 days)***	***Grade 2 persisting >7 days ****	***Grade 3 or 4***
	No reduction	Full dose oxaliplatin	Reduce oxaliplatin dose by 25%	Discontinue Oxaliplatin. Replace with Carboplatin AUC 5

** In case Grade 2 neuropathy persists for the entire 21-day cycle following cycle 1, delay cycle 2 by 1 week and re-start oxaliplatin at 75% if neuropathy resolves or reduced to Grade 1. If despite 1- week delay, neuropathy does not resolve, discontinue oxaliplatin and replace with carboplatin AUC5. Where oxaliplatin has been replaced with carboplatin, continue to use carboplatin during CRT (for dose/schedule during CRT, see section 8.4)*

Laryngopharyngeal dysaesthesia:

For patients developing acute laryngopharyngeal dysaesthesia during or shortly after the oxaliplatin infusion, subsequent oxaliplatin infusions should be given over 6 hours with caution.

Planter/palmar erythema (this is commonly related to capecitabine):

**Toxicity Grade**	**Action**
CTCAE Grade 1	Continue capecitabine.
CTCAE Grade 2	Withhold capecitabine until resolves to Grade 1. Restart with 15% dose reduction
CTCAE Grade 3	Withhold capecitabine until resolves to Grade 1. Restart with 25% dose reduction

CONFIDENTIAL
This material is the property of the Wales Cancer Trials Unit. Do not disclose or use except as authorised.

Diarrhoea, stomatitis, nausea/vomiting:

Incidence	CTCAE Grade 2	Grade 3	Grade 4
1st occurrence	Interrupt until resolved to G0-1, then resume capecitabine at original dose	Interrupt until resolved to G0-1, then resume capecitabine at 75% of original dose	Discontinue treatment altogether unless investigator considers this in the best interest of patient, in which case discuss with Chief investigator*
2nd occurrence of same toxicity	Interrupt until resolved to G0-1, then resume capecitabine at 75% of original dose	Interrupt until resolved to G0-1, then resume capecitabine at 50% of original dose	N/A
3rd occurrence of same toxicity	Interrupt until resolved to G0-1, then resume capecitabine at 50% of original dose and reduced oxaliplatin dose to 75% of previous dose level	Discontinue treatment*	N/A
4th occurrence of same toxicity	Discontinue treatment*	N/A	N/A

*Under these circumstances, patients should be withdrawn from trial treatment. The proposed treatment for these patients should be noted on the withdrawal CRF. Should the care for these patients still include surgery, please complete the surgery, pathology and follow up CRFs.

Renal Impairment:

- Oxaliplatin: Omit oxaliplatin if creatinine clearance < 30ml/min
- Capecitabine:

Creatinine clearance	Capecitabine dose level
≥ 50 ml/min	100%
30-49 ml/min	75%
< 30ml/min	omit

Hepatotoxicity:
Capecitabine
Isolated elevation in serum transaminases may be related to capecitabine and will not require dose interruption unless AST/ALT levels are ≥ 5 times ULN. If AST/ALT is above this level, capecitabine will be interrupted till it returns to ≤ 2.5 times ULN.

Oxaliplatin
If bilirubin > 3 x ULN, reduce dose by 50%

Respiratory toxicity:
Oxaliplatin is a rare cause of interstitial lung disease. In the case of unexplained respiratory symptoms such as non-productive cough, dyspnoea, crackles or radiological pulmonary infiltrates, oxaliplatin should be discontinued until further pulmonary investigations exclude an interstitial lung disease.

Coronary Artery Spasm:
For patients with a history of angina please ensure they have GTN spray at home and remain on their cardiac medication. Capecitabine induced coronary artery spasm will require permanent cessation of the drug. Further treatment/ trial involvement should be discussed with the Chief Investigator.

CONFIDENTIAL
This material is the property of the Wales Cancer Trials Unit. Do not disclose or use except as authorised.

DPD Deficiency:
Occasionally (approximately 1-3%) a patient may have a markedly exaggerated toxicity due to reduced 5FU catabolism. If this occurs, await full recovery of toxicities. Further treatment should be discussed with the Chief Investigator or one of the clinical co-investigators. If treatment is to continue it should be at a reduced capecitabine dose (e.g. 50%).

Where capecitabine is stopped for toxicity, the dose is omitted, not delayed. Tablets which have not been taken will be handed over to the trials nurse at subsequent visit.

8.4 Chemoradiotherapy toxicity and dose modification

Common toxicities of paclitaxel include: bone-marrow suppression, peripheral neuropathy, arthralgia, myalgia, alopecia, and allergic/hypersensitivity reactions. Detailed information can be found in the SPC available from www.medicines.org.uk.

Common toxicities of carboplatin include: bone-marrow suppression, nausea/vomiting, high tone hearing loss, asthenia, peripheral neuropathy, allergic reactions. Detailed information can be found in the SPC available from www.medicines.org.uk.

Arm A: Oxaliplatin and capecitabine haematological toxicity

**Neutrophil count once weekly during CRT**		**Platelet count once weekly during CRT**	**Action**
≥1.0	AND	≥75	Full dose oxaliplatin and capecitabine
<1.0	OR	≥25 - <75	Stop capecitabine and delay oxaliplatin till recovery. Restart capecitabine at 75% dose. Oxaliplatin dose reduced to 75% for subsequent cycles
ANY	AND	<25	Stop capecitabine and delay oxaliplatin till recovery. Restart capecitabine at 50% dose. Oxaliplatin dose reduced to 50% for subsequent cycles

Neurotoxicity (this is commonly related to oxaliplatin):

Toxicity	***Cold-induced dysaesthesia only***	***Grade 1 or 2 (if Grade 2, persists <7 days)***	***Grade 2 persisting >7 days but resolves before next cycle***	***Grade 2 neuropathy persisting for entire cycle length or Grade 3 or 4 neuropathy***
**Action**	No reduction	Full dose oxaliplatin	Reduce oxaliplatin dose by 25%	Discontinue Oxaliplatin. Use Carboplatin AUC 5 (weeks 1 and 4 if neuropathy develops prior to start of CRT; week 4 only if develops following the first dose of oxaliplatin during CRT). If Grade 3/4 neuropathy develops during week 4 or 5 of CRT, omit Oxaliplatin during week 5.

For non haeamatological toxicities associated with oxaliplatin or capecitabine, please refer to section 8.3 above

CONFIDENTIAL
This material is the property of the Wales Cancer Trials Unit. Do not disclose or use except as authorised.

Arm B: Paclitaxel and carboplatin haematological toxicity

Neutrophil count once weekly during CRT		Platelet count once weekly during CRT	Action
≥1.0	AND	≥75	Full dose paclitaxel and carboplatin
<1.0	OR	≥25 - <75	Omit both paclitaxel and carboplatin that week and omit chemotherapy weekly until recovery (N≥1.0 and pletelets≥75). Dose reduce by 25% for subsequent cycles
ANY	AND	<25	Omit that week's chemotherapy and delay weekly chemotherapy until recovery (N≥1.0 and pletelets≥75). Dose reduce both paclitaxel and carboplatin by 50% for subsequent weeks

Renal Impairment

Carboplatin contraindicated (so should be stopped) if GFR < 20ml/min

Paclitaxel - no dose modification required

Hepatic impairment

Carboplatin - no dose modification required

For paclitaxel there is limited information available. If bilirubin < 1.25x ULN, and ALT < 10 ULN, continue full intended dose. However if bilirubin > 1.25 x ULN further treatment should be discussed with the CI or one of the clinical Co-investigators.

Non-haematological toxicity

Due to overlapping toxicities for carboplatin and paclitaxel the instructions detailed below (interruption and potential dose reductions) should be applied to **both** drugs at each occurrence.

Incidence	CTCAE Grade 2	Grade 3	Grade 4
1st occurrence	Interrupt until resolved to G0-1, then resume at original dose	Interrupt until resolved to G0-1, then resume at 75% of original dose	Discontinue treatment altogether unless investigator considers this in the best interest of the patient, in which case Interrupt until resolved to G0-1, then resume at 50% of original doses*
2nd occurrence of same toxicity	Interrupt until resolved to G0-1, then resume at 75% of original dose	Interrupt until resolved to G0-1, then resume at 50% of original dose	N/A
3rd occurrence of same toxicity	Interrupt until resolved to G0-1, then resume at 50% of original dose	Discontinue treatment*	N/A
4th occurrence of same toxicity	Discontinue treatment*	N/A	N/A

*Under these circumstances, patients should be withdrawn from trial treatment. The proposed treatment for these patients should be noted on the withdrawal CRF. Should the care for these patients still include surgery, please complete the surgery, pathology and follow up CRFs.

This material is the property of the Wales Cancer Trials Unit. Do not disclose or use except as authorised.

8.5 Calculating and recalculating BSA/doses

BSA will be calculated according to DuBois and DuBois formula.
The patient's weight at baseline will be used to determine the dose of all chemotherapy drugs for the duration of the study. The patient's weight should then be recorded prior to every chemotherapy/chemoradiotherapy cycle. If a patient's weight changes by ≥10% from baseline then drug doses should be recalculated. If a patient's weight changes by <10% the dose may be adjusted according to local policy/clinician's discretion, but this is not an absolute requirement.

8.6 Dose capping

Doses for oxaliplatin, capecitabine and paclitaxel will be capped at BSA 2 m^2. The dose of carboplatin should be capped at 300mg unless the patient is receiving AUC 5 carboplatin in place of oxaliplatin for neurotoxicity dose modifications. In this situation, the GFR used in the Calvert formula for carboplatin dosing should not exceed 125ml/min.

8.7 Dose-banding

This will be permitted as per local hospital policy (as long as dose banding is within 5% of actual calculated dose).

8.8 Compliance

Patients will be instructed to keep a record of compliance in terms of their capecitabine treatment, by means of using a "patient diary" provided by WCTU. Patients should be asked to bring completed diary cards or other records and all their unused/remaining capecitabine tablets (empty, open or unopened) with them to each clinic visit. The patient diary card should not be sent to WCTU but kept by the centre to monitor patient drug compliance.

8.9 Concurrent Radiotherapy

In the case of chemotherapy toxicity or dose modification the decision as to whether to continue radiotherapy is at the discretion of the treating clinician. Patients with oesophageal carcinoma undergoing treatment with potentially curative radiotherapy should be treated as Category 1 patients ie prolongation of overall treatment time should be avoided, and certainly wherever possible not prolonged by more than 2 days over the original planned treatment duration (https://www.rcr.ac.uk/docs/oncology/pdf/BFCO(08)6_Interruptions.pdf).

Where the start of radiotherapy is delayed for scheduling reasons Day 1 of the third cycle of chemotherapy, ie Day 1 of the start of concurrent chemotherapy, should also be delayed such that the 2 treatments start together.

The decision as to the scheduling of chemotherapy as a result of delays to radiotherapy due to machine service days or breakdowns ie unscheduled interruptions to radiotherapy should be made at the clinical discretion of the local PI, although the first treatment of radiotherapy must given on the same first day of cycle 3 of the chemotherapy schedule.

8.10 Other medicinal treatments (i.e Non-Investigational Medicinal Products (NIMPS))

Support medication

Local anti-emetic policy may be followed.
The use of granulocyte-colony stimulating factor (G-CSF) is at the local investigator's discretion.

Concomitant medication

Concomitant medication may be given as medically indicated provided there is no interaction with chemotherapy (details below). All patients will be asked to provide a complete list of prescription and over-the-counter medications that have been taken within the previous 4 weeks prior to the first treatment visit. They must also inform the Investigator about any new medication started while in the trial.

CONFIDENTIAL
This material is the property of the Wales Cancer Trials Unit. Do not disclose or use except as authorised.

8.11 Drug interactions/potential drug interactions

Capecitabine interacts with several medications and the following precautions should be followed:

Absolute contra-indication:
- Sorivudine and analogues may produce dangerous interaction with capecitabine and must not be combined.

Drugs to be avoided:
- Methotrexate
- Allopurinol – may reduce effectiveness of capecitabine
- Warfarin – INR (International Normalised Ratio) is effected by capecitabine and patients should have warfarin stopped or changed to low molecular heparin depending on need for anti-coagulation

Drugs not contra-indicated but should be used with caution:
- Phenytoin – this may increase capecitabine levels
- Folic acid/folinic acid – reduces maximum tolerated dose, may increase capecitabine toxicity
- Aluminium Hydroxide and Magnesium Hydroxide antacids – has been shown to increase plasma concentration of capecitabine and its metabolite 5DFCR (5'-deoxy-5-fluorocytidine)
- Interferon alpha – reduces maximum tolerated dose of capecitabine
- Cytochrome P450 down regulation by capecitabine may affect the following classes of drugs – angiotensin II blockers [losartan, valsartan]; Oral hypoglycaemic agents [glipizide, tolbutamide, rosiglitazone]; NSAIDS [indomethacin, celecoxib, diclofenac, ibuprofen]

Detailed information is available from www.medicines.org.uk

8.12 Drug supply, accountability, labelling and disposal

The drugs in this trial should be supplied from a site's own stock. Chemotherapy should only be administered under the direction of Oncologists in specialist units under conditions permitting adequate monitoring and surveillance. Supportive equipment should be available to control anaphylactic reactions as per local practice.

Drug accountability is the responsibility of the PI on each site but can be undertaken by the site pharmacist listed on the trial delegation log. The drugs in this study shall be available through routine hospital supplies. Refer to the Summary of Products Characteristics (SPC) for full prescribing information and details of drug reconstitution, administration and stability (http://www.medicines.org.uk/emc/).

In order to comply with EU legislation, the following labelling and accountability directions must be followed (in addition to routine labels):

- Oxaliplatin, paclitaxel and carboplatin

Labelling: There is no requirement to label packs of these drugs. However, IV bags should be labelled to indicate that the patient is on a trial to allow for the periods when a patient may leave the oncology ward. These labels are included in the Pharmacy Pack provided by WCTU upon site activation

- Capecitabine

Labelling: Packs that are given to patients should be labelled with full information regarding the trial. These labels are included in the Pharmacy Pack provided by WCTU upon site activation.

Accountability logs for the IMPs (oxaliplatin, paclitaxel, carboplatin and capecitabine) are provided in the trial Pharmacy Pack provided by WCTU upon site activation. Drugs should be destroyed as per local practice.

NB Local labels and accountability logs can be used **as long as they contain exactly the same information as in the labels and logs provided.**

9 Radiotherapy

9.1 Introduction to radiotherapy

The protocol for NeoSCOPE has been developed through a UK Upper GI Radiotherapy Consensus Working Group.

The key points of reference for this work are the existing SCOPE 1 RT protocol; an Upper GI Radiotherapy Planning Workshop held October 27th 2011, Bristol, the EORTC-ROG Guidelines for neo-adjuvant radiation of adenocarcinomas of the GE junction and stomach (Matzinger 2009) and UK Patterns of Failure references (Button et al 2009, Dresner et al 2001.)

The key radiotherapy principles that will be used are outlined in this section, but detailed radiotherapy guidelines are provided in a separate document: NeoSCOPE Radiotherapy Planning Guidance Document, which can be accessed via the website http://www.rttrialsqa.org.uk. Any centres wishing to participate in this study will be asked to complete two 3D outlining exercises by each investigator supervising patients in the trial (one mid and one lower third oesophageal case), and a separate planning case. These cases will need to be centrally reviewed before patients can be recruited to the study. In addition, centres who wish to recruit patients to the 4-D radiotherapy component of the trial must attend a radiotherapy workshop or complete a separate pre-trial 4D exercise to ensure protocol compliance and that the necessary quality assurance standards are met.

For staging purposes, it is recommended that all patients have a CT scan of the thorax, abdomen (+/- pelvis) and an endoscopic ultrasound scan (EUS), noting the full extent of the disease with reference to anatomical landmarks. CT/PET has an established role in the UK in terms of staging oesophageal cancer and can be useful in determining the extent of disease, but the volume as defined by CT and EUS should not be reduced based on PET findings alone.

The total dose of radiation will be 45Gy in 25 fractions treating once daily, 5 days per week, prescribed and recorded as per ICRU 50/62. A single phase 3D-conformal treatment plan should be produced and delivered with multiple (usually 4-5) fields.

9.2 Target volume definition (TVD)

Patients fall into one of two separate groups:

- Middle 1/3 tumours, defined as a primary tumour starting between 24cm and 32 cm ab oral.
- Lower 1/3 tumours and GO junction, defined as any primary tumour starting from 32cm ab oral to less than 3cm distal to the anatomical GOJ.

This distinction accounts for the need to manually outline the elective nodal regions below the GOJ for lower 1/3 and GOJ tumours. There is also significant movement in this region, particularly due to respiration, which requires a larger PTV margin. Where possible, centres are encouraged to participate in the 4-D CT planning sub-study, which is only for patients with lower 1/3 or GO junction tumours.

9.2.1 Middle 1/3 tumours

GTV consists of the gross tumour, any involved nodes and the circumference of the oesophagus at that level. Any intervening oesophagus between the primary and nodal areas is also included.

GTV

The GTV is copied and labelled 'CTVA' and is grown manually to include the circumference of the oesophagus 20mm superiorly and inferiorly if defined by primary tumour, and by 10mm where the proximal/distal margins are defined by nodal disease.

CTV

CTVA is then copied and labelled 'CTVB'. It is grown by adding 10mm in right-left and anterior-posterior directions using the Treatment Planning System (TPS). CTVB is then edited for barriers of spread to exclude lung, pericardium, large vessels, trachea and right/left main bronchi and the vertebrae.

PTV

CTVB is grown in the superior – inferior direction by 20mm, and 10mm circumferentially using the treatment planning system and labelled PTV. Posteriorly this margin may be reduced if the PTV extends across the vertebrae by more than 5mm. Further information is provided in the Radiotherapy Guidance Document.

9.2.2 Lower 1/3 tumours

GTV

Above the GO junction, GTV is outlined as described above for middle 1/3 tumours.
Below the GO junction the primary and any involved nodes are outlined separately. The primary tumour below the GOJ is incorporated within the GTV whereas the nodes below the GOJ are outlined and labelled separately as GTVN (GTVN1, GTVN2 etc).

CTV

GTV is copied and labelled 'CTVA'. Superiorly this is grown manually along the axis of the oesophagus and should be 20mm where the proximal margin is defined by primary tumour and 10mm superior where this is defined by nodal disease. The whole circumference of the oesophageal wall should be included.

GTVN is/are grown by a 5mm margin in all directions and labelled 'CTVN5mm'. This defines a minimum margin around positive nodes to assist in the delineation of CTVB.

CTVA is copied and labelled 'CTVB'. It is grown by adding 10mm in right-left and anterior-posterior directions using the Treatment Planning System (TPS). Above the diaphragm CTVB is edited to exclude lung, pericardium, large vessels and the vertebrae. Below the GOJ CTVB is grown manually to include the volume at risk to a total of 20mm below GTV. This volume includes CTVN 5mm, and the elective nodal regions at high risk of microscopic spread. Full details and examples are given in the Radiotherapy Planning Guidance Document.

PTV

CTVB is copied and grown using the TPS by 15mm superiorly, 20mm inferiorly and 15mm circumferentially and labelled PTV. The posterior margin may be reduced if the PTV extends across the vertebrae by more than 5mm.

9.2.3 Lower 1/3 and GOJ tumour 4D CT target definition protocol.

The principle involved in 4D planning is to account for intra-fraction motion and therefore generate patient specific volumes. Please refer to the Radiotherapy Guidance Document for full details of acquisition and outlining on the 4D dataset.

9.3 Organs at risk

The spinal cord should be outlined on slices which include or are within 20mm of the PTV in the superior and inferior directions.

A Planning Risk Volume (PRV) for the cord is created to account for positioning error; the size of the margin added to the cord being commensurate with the accuracy of treatment delivery expected and, as such, the tolerance level allowed in portal image verification on treatment.

The right and left lungs are outlined in such a way that the planning system will be able to calculate a combined lung Dose Volume Histogram (DVH).

The whole heart should be outlined to the extent of the pericardial sac (if visible). The major blood vessels (superior to the organ) and the inferior vena cava (towards the inferior extent of the heart) are excluded.

The whole liver is outlined if the level of its superior edge overlaps with the level of the inferior extent of the PTV.

Each kidney is outlined separately if the level of its superior edge overlaps with the level of the inferior extent of the PTV.

CONFIDENTIAL
This material is the property of the Wales Cancer Trials Unit. Do not disclose or use except as authorised.

The whole stomach should be outlined in such a way that a stomach DVH can be produced. This will be for evaluation only as a Region of Interest (RoI) and will not be an OAR with dose constraints.

Example of heart and stomach outlining are provided in the RPGD.

9.4 Treatment Plan Optimisation

A single phase 3D conformal treatment plan should be produced. The use of a 'type B' calculation algorithm is strongly recommended for dose calculation and optimisation.

9.5 Dose limitations of Volumes of Interest (VOI) and of Organs at Risk (OAR)

Region of Interest / Organ at Risk	Dose Objective	Comments/ Secondary Considerations
PTV if 'type B' algorithm used	V95% ≥ 99% – (0.4*%lung/PTV overlap)	V95% objective is individually determined based on the percentage of PTV which overlaps with lung tissue according to the formula adopted from Wills et. al. [4]
PTV if 'type A' algorithm used	V95% > 99%<br>and<br>D99% > 95%	
ICRU Maximum dose	D1.8cc < 107%	Defined as the maximum dose to 1.8cc of any structure within the external contour of the patient.
Spinal Cord PRV	D1cm3 < 40Gy	If the PTV lies close to or overlaps with the Spinal Cord PRV, the treating clinician may discretionally allow a point maximum dose up to 45Gy. Alternatively, they may report a PTV compromise (for type b algorithms this compromise must be noted regardless of the plan achieving the individualised PTV objective).
Combined Lungs	V20Gy < 25%	The aim should be to minimise dose to the lung wherever possible to V20Gy < 20%<br>Total lung volume and V5 lung, V5s lung and mean lung dose will be collected and reported on PAF but will not be a volume/dose constraint
Heart	V40Gy < 30%<br><br>V25Gy < 50%	 <br>Optimal objective – to be achieved where possible but at lower priority then other objectives.
Liver	V30Gy < 60%	
Individual Kidneys	V20Gy < 25%	
Stomach		This will not be defined as an organ at risk but data collected to explore correlation with toxicity.

9.6 Treatment Verification

It is recommended that the best available positional verification methods should be used to ensure correct delivery. The use of cone beam CT matched to planning CT scans is highly recommended. If this is not available the minimum treatment positional verification requires the collection of electronically acquired lateral and anterior portal isocentre images (EPIs) (or films where electronic means are not available) compared to treatment planning DRRs.

The minimum protocol for on-treatment verification is for imaging the initial three fractions so that a correction for systematic error can be applied and then continue with weekly imaging. The isocentre should be moved if disagreement is seen in excess of agreed tolerance levels based on local study – typically 5mm. This process also allows radiographers to evaluate the whole set-up and thus to assess and correct systematic errors. Using EPI the MLC configuration can also be verified for consistency and reproducibility.

CONFIDENTIAL
This material is the property of the Wales Cancer Trials Unit. Do not disclose or use except as authorised.

9.7 The management of unscheduled gaps in radiotherapy treatment

In the event of unscheduled gaps to radiotherapy treatment, these should be managed in line with the latest RCR Guidance (The Timely Delivery of Radical Radiotherapy: Standards and Guidelines for the Management of Unscheduled Treatment Interruptions, Third Edition, 2008 - full document available at www.rcr.ac.uk). In the case of cancers of the oesophagus, adenocarcinoma should be managed as Category 1 patients.

9.8 Radiotherapy quality assurance

9.8.1 Pre-trial quality assurance

Completion of an outlining exercise:
All centres who wish to participate in NeoSCOPE must satisfactorily complete a pre-trial outlining benchmark case of a mid and lower oesophageal cancer case. Data should be uploaded using the RTTQA website. Outlines will be compared against a consensus reference volume (gold standard) derived from the outlines of TMG members.

Criteria for satisfactory completion will be at the discretion of the RTQA members of the TMG. Attention will be paid to correct interpretation of imaging (GTV) and ability to follow the protocol to create CTV and PTV. Written feedback will be provided to all centres.

For 4D centres attendance at either a workshop or completion of a 4D pre-trial test case will be mandated as part of the pre-trial QA of the 4D outlining. A 4D test case will need be completed in addition to a 3D case.

Completion of a planning exercise:
One pre outlined patient per centre should be planned and the data uploaded following the instructions on the RTTQA website. A Plan Assessment form (PAF) should be completed and submitted at the same time.

Production of a Radiotherapy Process Document – in line with which all trial patients will be scanned, planned and treated.

Completion of questionnaires:
The following questionnaires should be completed:

National radiotherapy trials QA baseline questionnaire
National radiotherapy trials QA staff questionnaire

9.9 On-trial quality assurance

Full planning data for each patient (planning CT, structures, plan and dose) should be uploaded following the instructions on the website above. A PAF should be completed and submitted at the same time.

Real time review
There will be real-time review of the outline and plan for the first patient case from each centre and all cases submitted up until the first NeoSCOPE toxicity analysis assessment. A second case will also be reviewed should there has been an issue with the first case.

3D outlining assessment will be in 'real time' and undertaken by the QA NeoSCOPE subgroup. As such, real time review will require timely uploading of the data from the centres. We request that CT data and plan assessment forms (PAFs) be submitted via a secure NHS server. Copies of the PAF should be accessed from the RTTQA website. Please also submit the reports for the CT, EUS and PET scan. The QA NeoSCOPE subgroup will process the review within 3 working days of receipt. Early submission of the outlining data is strongly encouraged to allow adequate time for review prior to the start of radiotherapy planning. It is left to the centre's discretion as to whether they wish to start the planning process with the pre-approval outlines while awaiting this feedback.

CONFIDENTIAL
This material is the property of the Wales Cancer Trials Unit. Do not disclose or use except as authorised.

Non-real time review

As the timing for non-real time review is not a primary concern, data and a copy of the PAF can be sent via an NHS secure network (as above) or via CD-ROM. 10% of non-real time cases will be randomly chosen for review.

10 Surgery

Surgery should be conducted between 6 to 8 weeks after completion of chemoradiotherapy. A CT scan should be performed after completion of chemoradiotherapy in the weeks prior to surgery to rule out disease progression outside of the radiotherapy volume.

The operation will usually consist of a two-phase oesophagectomy (abdomen and right or left chest approach) (the use of an extended total gastrectomy for small junctional cancers will require discussion with the CI) with a two-field lymphadenectomy (abdomen and thorax). The abdominal phase should be carried out first. The stomach is the preferred organ for reconstruction. In order to standardise surgical procedures across the treatment arms, the surgical procedures described below should be performed for both treatment arms.

Lymph node numbers referred to are those used in the Japanese classifications for oesophageal and gastric carcinoma.

Centres who wish to enter patients into the NeoSCOPE trial and perform the operation as a total minimally invasive procedure should contact the Trial Manager. In order to maintain surgical quality assurance, each surgeon who wishes to perform a total minimally invasive procedure will be asked to provide a summary of evidence of their previous 20 total minimally invasive operations documenting anastomotic leak rates, CRM involvement rates, lymph node yields in both the abdomen and the mediastinum and post-operative Clavien-Dindo complication rates.

10.1 The abdominal phase

1. The incision is at the surgeon's preference.
2. The intra-abdominal contents should be carefully inspected, paying particular attention to the omentum and peritoneal surfaces in the supracolic compartment for peritoneal metastases and the para-aortic region in patients with otherwise resectable nodal disease. Suspicious peritoneal deposits, enlarged para-aortic nodes and any other lesion considered consistent with haematogenous metastasis, should be dealt with by frozen section biopsy. The operation should be terminated if there is unequivocal evidence on frozen section that there is disease outside the proposed surgical field.
3. Complete gastric mobilisation should be achieved, based on the right gastroepiploic and right gastric arteries. Where the stomach is unavailable as a suitable conduit, colonic transposition should be performed according to surgeon's preference. Pyloroplasty, pyloromyotomy or no drainage are options at the surgeons' preference.
4. The coronary vein should be divided as low as possible, and the left gastric artery should be divided at its origin. The lymph nodes on the left gastric artery and lesser curve should be included en bloc. Lymphadenectomies along the hepatic artery and splenic artery will be performed en bloc. This should only be resected separately in special circumstances, the reasons for which should be recorded.
5. The hepatic artery dissection should remove all nodal tissue overlying the hepatic artery proper and the common hepatic artery to ensure removal of all group 8 nodes.
6. Lymphadenectomy related to the splenic artery should extend from the origin of this vessel as far lateral as the point of ligation of the uppermost posterior short gastric vessel ensuring complete removal of station 11 nodes.
7. The dissection at the diaphragm is designed to minimise the risk of a positive circumferential resection margin. The exact extent of this dissection will be influenced by the results of pre-operative staging, as well as intra-operative assessment. The aim of the surgery should be to remove sufficient crural fibres and a cuff of diaphragm, to minimise the risk of local recurrence when the primary tumour is at this level. Mobilisation of the left lateral segments of the liver, division of the inferior phrenic vein well to the right and to the left of the oesophagus, facilitates excision of an inverted V-shaped segment of diaphragm in continuity with the crura.

8. Removal of the pericardial fat pad anteriorly and strips of parietal pleura should usually be achieved at this stage, to again minimize the risk of a positive circumferential margin, although these steps can be undertaken during the thoracic phase of the operation.
9. Preparation of the transaction site on the lesser curve, without compromise to the extent of lymphadenectomy, can be undertaken at this stage or during the thoracic phase at the surgeon's discretion.
10. The abdominal phase should result in a dissection which has removed lymph nodes from stations 1, 2, 3, 7, 8 and 11.
11. The placement of a feeding jejunostomy, intra-abdominal and intra-thoracic drains at the surgeon's discretion.

10.2 Thoracic Phase

1. The chest can be opened through either a right or left thoracotomy. The mediastinal pleura overlying the oesophagus should be excised in continuity with the oesophagus.
2. The posterior limit of the dissection should be the antero-lateral wall of the aorta, so that the thoracic duct is mobilized with the oesophagus and peri-oesophageal tissues.
3. The thoracic duct is ligated and divided at the level of the diaphragm and at the upper level of transaction. The duct may be removed according to local practice but will require discussion with the CI.
4. Having encircled the oesophagus at or close to the diaphragm (preferably during the abdominal phase), the mediastinal pleura overlying the left side of the oesophagus should also be excised to above the level of the tumour.
5. Para-oesophageal and diaphragmatic nodes (groups 108, 110, 111) are removed in continuity with the oesophagus.
6. Lymph nodes at the tracheal bifurcation and along the right and left main bronchi to the pulmonary hilus (nodal groups 107, 109), should be removed en bloc. They should only be resected separately in special circumstances, the reasons for which should be recorded.
7. The extent of lymphadenectomy for the upper thoracic para-oesophageal nodes (group 105), will be determined by the site of oesophageal transection. This must be above the aortic arch and preferably within 5 cm of the thoracic inlet.
8. Enlarged nodes in the para-tracheal group (group 106), should be removed for sampling purposes. NB – complete dissection of the left sided recurrent laryngeal nerve nodal chain is not mandatory.
9. The anastomotic technique and method of chest drainage is at the surgeon's discretion.
10. Orientation sutures have to be attached to the resection specimen preferably whilst the specimen is still in situ – a long suture onto the anterior surface of the stomach and a long suture onto the anterior surface of the oesophagus. In addition, a short suture has to be attached to the right side of the oesophagus assuming a right sided thoracic approach. For the left sided approach, the short suture can be attached on the left side. The presence of the sutures and their meaning should be documented on the histopathology request form that accompanies the resection specimen to the pathology lab. It is expected that the resection specimen will be submitted to the pathology laboratory fresh (e.g. not in formalin) and 'intact' e.g. unopened and with all peri-oesophageal and peri-gastric tissue attached. Should the surgeon wish to dissect the lymph nodes him/herself, it is mandatory that the peritumoural lymph nodes are not dissected off the specimen as this will compromise the assessment of the circumferential resection margin. All other non-peritumoural lymph nodes can be dissected off the specimen should the surgeon wish to do so.

11 Trial assessments

11.1 Screening assessments

See section 6.1.

CONFIDENTIAL
This material is the property of the Wales Cancer Trials Unit. Do not disclose or use except as authorised.

11.2 Baseline assessments

Screening assessments should be used to populate the baseline CRFs if the patient consents and enters into the trial.

11.3 Assessments on treatment

Prior to cycle 1

If the patient has given consent to donate samples for translational research (T-NeoSCOPE – see section 15) then:

- 2 x 10ml whole blood samples should be taken
- the pre-treatment diagnostic biopsy blocks should be requested from pathology.

Prior to each cycle of neoadjuvant chemotherapy

Neoadjuvant chemotherapy should be started within 3 weeks of randomisation.

The following assessments should be done within 3 working days prior to the start of each cycle. If the medical review happens to be outside 3 working days because of clinic days, the research nurse or a member of the medical team needs to review the patient and perform appropriate assessments as per protocol within the 3 working day window to confirm that there has been no deterioration in the patient's condition.

- Clinical examination
- Haematology: haemoglobin, leukocytes, platelets, differential white cell count
- Biochemistry: urea, creatinine, sodium, potassium, magnesium, corrected calcium, albumin, bilirubin, AST and/or ALT, ALP and phosphate. EDTA/24 hour clearance should be performed if calculated creatinine clearance (using the Cockcroft-Gault formula) has deteriorated by more than 25% (see Appendix 3)
- Assessment of NCI CTCAE according to version 4.03. This will be the toxicity assessment for the previous cycle and should incorporate any toxicity experienced by the patient after the start of the previous cycle.

NB During neoadjuvant chemotherapy a radiotherapy planning CT scan should be done. **The planning CT data, completed Plan Assessment Form, the EUS and the PET report should be uploaded to the NCRI Radiotherapy Clinical Trials Quality Assurance Group website as part of the NeoSCOPE RTQA process (see section 9.5).** Current versions of the Plan Assessment Form and the Radiotherapy guideline document can be uploaded from the Radiotherapy Clinical Trials Quality Assurance Group website.

During chemoradiotherapy

Chemoradiotherapy should commence immediately after neoadjuvant chemotherapy. If there is a >3 week delay in starting CRT due to toxicity from neo-adjuvant chemotherapy, CRT should be abandoned and patient should proceed directly to surgery.

Patients will be followed weekly during CRT. The following assessment should be performed:

- Clinical examination; Assessment of NCI CTCAE according to version 4.03. This will be the toxicity assessment for the previous cycle and should incorporate any toxicity experienced by the patient from the start of the previous cycle.
- Haematology: haemoglobin, leukocytes, platelets, differential white cell count
- Prior to chemotherapy only: Biochemistry: urea, creatinine, sodium, potassium, magnesium, corrected calcium, albumin, bilirubin, AST and/or ALT, ALP and phosphate. EDTA/24 hour clearance should be performed if calculated creatinine clearance (using the Cockcroft-Gault formula) has deteriorated by more than 25% (see Appendix 3)

End of chemoradiotherapy

Within 7 days of last fraction of radiotherapy:

- Clinical examination
- Assessment of NCI CTCAE according to version 4.03

Pre surgery

CONFIDENTIAL
This material is the property of the Wales Cancer Trials Unit. Do not disclose or use except as authorised.

- 4 weeks post CRT: Spiral/multi-slice CT with oral contrast or water **to rule out progression outside of the radiotherapy volume**. Maximum slice width 5mm. IV contrast/venous phase. CT must include abdomen and chest. Neck and pelvis fields are optional
- Clinical examination

30 days post surgery

- Clinical examination
- Postoperative complications
- Diagnostic and postoperative histology slides to be sent for central review along with photographs of the resected specimen– see Appendix 4.

11.4 Follow up assessments – 6 months and 12 months post surgery

- Clinical examination
- Assessment of NCI CTCAE according to version 4.03

A detailed assessment schedule is given in section 11.6 overleaf.

11.5 Completion of CRFs

The top copy of each completed CRF should be returned to the WCTU for data entry within four weeks of the visit. The remaining copy is to be retained at the local site. In accordance with the principles of GCP, the PI is responsible for ensuring accuracy, completeness, legibility and timeliness of the data reported to the WCTU in the CRFs.

CRF pages and data received by the WCTU from participating trial sites will be checked for missing, illegible or unusual values (range checks) and consistency over time. If missing or questionable data are identified, a data query will be raised on a data clarification form. The data clarification form will be sent to the relevant participating site. The site shall be requested to answer the data query or correct data on the data clarification form. The case report form pages should not be altered. All answered data queries and corrections should be signed off and dated by a delegated member of staff at the relevant participating site. The completed data clarification form should be returned to the WCTU and a copy retained at the site along with the participants' CRFs. The WCTU will send reminders for any overdue data. It is a site's responsibility to submit complete and accurate data in timely manner.

CONFIDENTIAL
This material is the property of the Wales Cancer Trials Unit. Do not disclose or use except as authorised.

11.6 Schedule of trial assessments

	Screening			Neoadjuvant chemotherapy (to start within 3 weeks of randomisation)			Chemo-radiotherapy	Surgery		Follow up	
	Normally within 4 weeks (see section 6.1) prior to randomisation	Within 7 days prior to randomisation	Within 14 days prior to start of treatment	Wk 1 Pre-cycle 1[G]	Wk 4 Post-cycle 1[G]	Wk 6 Post Cycle 2[G]	Wk 7-11 At end of each week[G]	Wk 17-19 Pre surgery assessments	30 day post op review	6 months post surgery	12 months post surgery
**Assessments**											
Consent	X										
Clinical examination[A]		X[B]		X	X	X	X	X	X	X	X
CT scan abdomen and chest[C]	X				RT planning scan			X			
EUS	X										
Laparoscopy/bone scan	optional										
PET/CT scan	recommended										
Spirometry	X										
ECHO or MUGA	X										
ECG	X										
FBC[D], GFR[E], Serum biochemistry[F], LFT		X		X	X	X	X				
Pregnancy test		X									
Toxicity assessment (CTCAE v4.03)				X	X	X	X			X	X
Post operative complications									X		
Histology	XH								X[H]		
**T-NeoSCOPE** Blood samples			X								
**T-NeoSCOPE** Biopsies samples	Diagnostic biopsy										

N.B. Serious Adverse Events (SAE) will be collected in real time via a designated SAE fax number.

[A]As per routine
[B]Including WHO performance status (see Appendix 2)
[C]Neck and pelvis fields are optional
[D]FBC to include haemoglobin, leukocytes, platelets and differential white cell count
[E]Measured or estimated (see Appendix 2)
[F]Biochemistry to include: urea, creatinine, sodium, potassium, magnesium, corrected calcium, albumin, bilirubin, AST and/or ALT, ALP and phosphate.
[G]All cycle assessments are done within 3 working days before the start of each treatment cycle.
[H] Diagnostic and postoperative histology slides and pathology report should be sent for histopathology review along with photographs of the resected specimen. See the NeoSCOPE Histopathology Guidelines (Appendix 4)

This material is the property of the Wales Cancer Trials Unit. Do not disclose or use except as authorised.

12 Safety reporting and pharmacovigilance

The Principal Investigator is responsible for ensuring that all site staff involved in this trial are familiar with the content of this section.

The following definitions are in accordance with the Medicines for Human Use (Clinical Trials) Regulations 2004 (SI2004/1031) (as amended) and EU Directive 2001/20/EC.

Term	Definition
**Adverse Event (AE)**	Any untoward medical occurrence in a patient or clinical trial participant administered a medicinal product and which does not necessarily have a causal relationship with this treatment. An adverse event can therefore be any unfavourable and unintended sign (including an abnormal laboratory finding for example), symptom or disease temporally associated with the use of a medicinal product, whether or not considered related to the medicinal product.
**Adverse Reaction (AR)**	Any untoward and unintended response in a clinical trial participant to an investigational medicinal product which is related to any dose administered to that participant
**Serious Adverse Event (SAE)**	Any adverse event that -<br>• Results in death<br>• Is life-threatening*<br>• Required hospitalisation or prolongation of existing hospitalisation**<br>• Results in persistent or significant disability or incapacity<br>• Consists of a congenital anomaly or birth defect<br>• Other medically important condition ***<br>In addition for the purposes of this trial the following events will also be considered SAEs and must be captured on the SAE form and reported to the WCTU with 24hours of knowledge of the event:<br>• All deaths within 30 days of surgery should be reported as SAEs<br>For the purposes of this trial the following events will not require immediate reporting<br>• Hospitalisation as a result of disease progression (for example, procedures involving the insertion of a PEG or stent as these are procedures for routine management)<br>• Hospitalisation as a result of expected toxicities (grade 1 or 2) of chemotherapy or RT (see table above)<br>These should be completed in the participants notes and on the relevant toxicities CRF page and forwarded to the WCTU in the normal timeframes for CRFs.
**Serious Adverse Reactions (SARs)**	Any SAE occurring in a clinical trial participant for which there is a reasonable possibility that it is related to the IMP at any dose administered.
Suspected Unexpected Serious Adverse Reactions (SUSARs)	A SAR, the nature and severity of which is not consistent with the Reference Safety Information (RSI) for the IMP.

*Note: The term 'life-threatening' in the definition of serious refers to an event in which the trial participant was at risk of death at the time of the event or it is suspected that used or continued used of the product would result in the subjects death; it does not refer to an event which hypothetically might have caused death if it were more severe.

** Note: Hospitalisation is defined as an inpatient admission, regardless of the length of stay, even if the hospitalisation is a precautionary measure for continued observation. Pre-planned hospitalisation e.g. for pre-existing conditions which have not worsened, or elective procedures, does not constitute an SAE.

*** Note: other events that may not result in death, are not life-threatening, or do not require hospitalisation, may be considered as an SAE when, based upon appropriate medical judgement, the event may jeopardise the participant and may require medical or surgical intervention to prevent one of the outcomes listed above.

12.1 Causality Assessments

The Principal Investigator (or another delegated medically qualified doctor from the trial team) and Chief Investigator (or another medically qualified doctor from the Trial Management Group) will assess each SAE to determine the causal relationship with the IMP, and will answer 'yes' or 'no' to the question "Do you consider that there is a reasonable possibility that the SAE may have been caused by the IMP?"

For SAEs causal relationship will also be assessed for other trial treatments (nIMPs) and procedures.

IMPs: Oxaliplatin, capecitabine, carboplatin and paclitaxel.

The causality assessment given by the Principal Investigator (or delegate) cannot be downgraded by the Chief Investigator (or delegate), and in the case of disagreement both opinions will be provided.

A guide to the interpretation of the causality question is found in Appendix 1 of this clinical trial protocol.

12.2 Expectedness Assessments

The Chief Investigator (or another delegated appropriately qualified individual) will assess each SAE to perform the assessment of expectedness.

The expectedness assessment should be made with reference to the current Reference Safety Information (RSI), and must be applied to all IMPs in the trial. Expectedness decisions must be based purely on the content of the RSI; other factors such as the participant population and participant history should not be taken into account. Expectedness is not related to what is an anticipated event within a particular disease.

SAEs which add significant information on specificity or severity of a known, already documented adverse event constitute unexpected events. For example, an event more specific or more severe than that described in the RSI is considered unexpected.

The table below lists the RSI's that should be referenced

IMP	RSI to be used for expectedness assessment	Relevant section of RSI to be used for expectedness assessment
Capecitabine	SPC to be used (19/02/2015).<br>Manufacturer: *Roche	Section 4.8
Oxaliplatin	SPC to be used (powder 08/02/2012).<br>Manufacturer: *Accord Healthcare	Section 4.8
Carboplatin	SPC to be used (29/02/2012).<br>Manufacturer: *Accord Healthcare	Section 4.8
Paclitaxel	SPC to be used (24/08/2012).<br>Manufacturer: *Hospira UK Ltd	Section 4.8

*Please note that hospital supply stock may be used even if the manufacturer differs from that listed above. However, please use the SPCs listed above when assessing expectedness.

The table below lists the expected events in relation to surgery and this should be used as the RSI when assessing the expectedness of SAEs causally related to surgery:

**Bleeding**	**Genitourinary**	**Neurological**
Anaemia requiring transfusion<br>Post-operative bleed other than gastrointestinal<br>Wound haematoma	Renal failure<br>Urinary retention	Delirium / agitation<br>Loss of consciousness<br>Vertigo
**Cardiac**	**Infectious**	**Pulmonary**
Angina<br>Arrhythmia<br>Congestive heart failure<br>Hypertension<br>Hypotension<br>Myocardial infarction	Abscess<br>Fever of unknown origin (FUO)<br>Systemic sepsis<br>Urinary tract infection (UTI)	Atelectasis<br>Pleural effusion<br>Pneumonia<br>Pneumothorax<br>Respiratory distress
**Gastrointestinal**	**Wound infection**	**Surgical**
Clostridium difficile colitis<br>Constipation (inability to have a bowel movement postoperative day 5 with no signs of ileus or SBO<br>Diarrhoea<br>Emesis	Deep or superficial wound dehiscence<br>Wound infection<br>Wound seroma	Bowel injury<br>Incisional hernia<br>Retained foreign body<br>Vascular injury<br>Thoracic duct injury<br>Cranial nerve and /or sympathetic chain injury<br>Brachial plexus injury
**Miscellaneous**	**Thromboembolic**	
Acidosis<br>Decubitis ulcer<br>Dehydration<br>Lymphocele<br>Peripheral arterial ischemia<br>Psychological illness<br>Thrombocytopenia	Deep vein thrombosis (DVT)<br>Superficial phlebitis<br>Pulmonary embolism	

Note: Many of the side effects to surgery can be exacerbated by CRT (ie for all surgical complications we say possibly related to CRT)

The following lists the expected events in relation to radiotherapy and this should be used as the RSI when assessing the expectedness of SAEs causally related to radiotherapy:

It is expected that patients receiving radiotherapy for oesophageal cancers may require admission for symptom control of the following:

CONFIDENTIAL
This material is the property of the Wales Cancer Trials Unit. Do not disclose or use except as authorised.

Mucositis, oesophagitis, dysphagia, lethargy, pain, anaemia, nausea and vomiting, weight loss, poor oral intake, tinnitus (chemotherapy related), infection, pneumonitis, pericarditis, angina, wound infection, anastamotic leak, chest infection, pleural effusion, thrombo-embolism

They may require intravenous hydration and/or nasogastric feeding tube (NG) feeding and pain control.
Note: For the purposes of this protocol anticipated treatment related SAEs of grade ≤3 are not subject to expedited reporting but are recorded in the CRFs.

12.3 SAE reporting

12.3.1 Participating Site Responsibilities

All SAEs must be reported immediately (and within 24 hours of knowledge of the event) by the PI at the participating site to the WCTU unless the SAE is specified as not requiring immediate reporting (see above). This includes SAEs related to IMPs and non-Investigational Medicinal Products (nIMPs).

The PI (or delegated medically qualified doctor from the trial team) should sign and date the SAE CRF to acknowledge that he/she has performed the seriousness and causality assessments.

A completed SAE form for all events requiring immediate reporting should be faxed to the WCTU within 24 hours of knowledge of the event. A separate form must be used to report each event, irrespective of whether or not the events had the same date of onset.

The participant will be identified only by trial number, date of birth and initials. The participant's name should not be used on any correspondence.

It is also required that sites respond to and clarify any queries raised on any reported SAEs and report any additional information as and when it becomes available through to the resolution of the event.

Serious Adverse Event (SAE) Fax Number:
029 2064 4488

Serious adverse events should be reported from time of signature of informed consent, throughout the treatment period up to, and including 30 days after the participant receives their last dose of the IMP. Serious adverse reactions (such as long term side effects of trial treatment under investigation) should continue to be reported until the end of follow up.

Adverse events (AE) should be graded using the NCI Common Terminology Criteria for Adverse Events (CTCAE) Version 4.03. The toxicity grades should be recorded on the toxicity part of the CRF.

An SAE form is not considered as complete unless the following details are provided:

- Full participant trial number
- An Adverse Event / Adverse Reaction
- A completed assessment of the seriousness, and causality as performed by the PI (or another appropriately medically qualified doctor registered on the delegation log).

If any of these details are missing, the site will be contacted and the information must be provided by the site to the WCTU within 24 hours.

All other AEs should be reported on the CRF following the CRF procedure described in Section 11.5.

12.3.2 The WCTU responsibilities

Following the initial report, all SAEs should be followed up to resolution wherever possible, and further information may be requested by the WCTU.

The WCTU should continue reporting SAEs until 30 days after the participant receives their last dose of the investigational medicinal product. Serious adverse reactions should continue to be reported until the end of follow up. Once an SAE is received at the WCTU, it will be evaluated by staff at the WCTU and sent to the Chief Investigator (or their delegate) for an assessment of causality and expectedness.

Investigator reports of suspected SARs will be reviewed immediately and those that are identified as SUSARs are reported to the MHRA and the main Ethics Committee.

12.4 SUSAR reporting

Velindre NHS Trust is undertaking the duties of trial Sponsor and has delegated to the WCTU the responsibility for reporting SUSARs and other SARs to the regulatory authorities (MHRA and relevant ethics committees) as follows:

SUSARs which are fatal or life-threatening must be reported to the MHRA and MREC within 7 calendar days of receipt at the WCTU. Any additional, relevant information must be reported within a further 8 calendar days of submitting the initial report.

SUSARs that are not fatal or life-threatening must be reported to the MHRA and MREC within 15 days of receipt at the WCTU. Any additional, relevant information must be reported within a further 15 days.
N.B. There is no requirement for WCTU to report SUSARs to nIMPs to the MHRA except in the following instances:

- If the adverse reaction is suspected to be linked to an interaction between a nIMP and IMP, and is serious and unexpected, WCTU should report as a SUSAR due to the interaction with the IMP.
- If a SUSAR is suspected and might be linked to either a nIMP or an IMP and cannot be attributed to only one of these.
- If the adverse reaction due to the nIMP is likely to affect the safety of trial subjects then WCTU should report it to the MHRA and Main Ethics Committee in accordance with the relevant Standard Operating Procedure for reporting Urgent Safety Measures.

12.5 Safety Reports

A list of all SARs (expected and unexpected) will be reported annually to the MHRA, Main Ethics Committee and the trial sponsor in a Development Safety Update Report (DSUR). This report must be submitted within 60 days of the anniversary of the MHRA CTA approval date.

The WCTU will report a list of all SARs (expected and unexpected) and any other safety recommendations to all PIs every 6 months throughout the course of the trial. This frequency may be reviewed and amended as necessary. This reporting will be done via the Investigator safety report (ISR).

12.6 Pregnancy reporting whilst participating in the NeoSCOPE trial

Pregnancy, or the pregnancy of a partner occurring whilst participating in the NeoSCOPE trial, although not considered an SAE, must be notified to the WCTU within the same timelines as an SAE (i.e. during the trial treatment period and up to 30 days after the last date of treatment).

CONFIDENTIAL
This material is the property of the Wales Cancer Trials Unit. Do not disclose or use except as authorised.

In the event of a pregnancy, if the participant or the female partner of a male participant have read the Pregnancy Information Sheet, and signed the Pregnancy Consent Form, the WCTU must be contacted immediately to request a Pregnancy Report Form. The Pregnancy Report Form should be completed and returned to the WCTU to capture all the relevant information required for the expedited reporting of these events.

The outcome of a pregnancy should be followed up carefully and any abnormal outcome of the mother or the foetus should be reported. This also applies to pregnancies following the administration of the IMP to the father prior to sexual intercourse.

A congenital anomaly or birth defect is considered an SAE and should be reported to the WCTU within 24 hours of knowledge of the event.

CONFIDENTIAL
This material is the property of the Wales Cancer Trials Unit. Do not disclose or use except as authorised.

12.7 Flowchart for Serious Adverse Event reporting

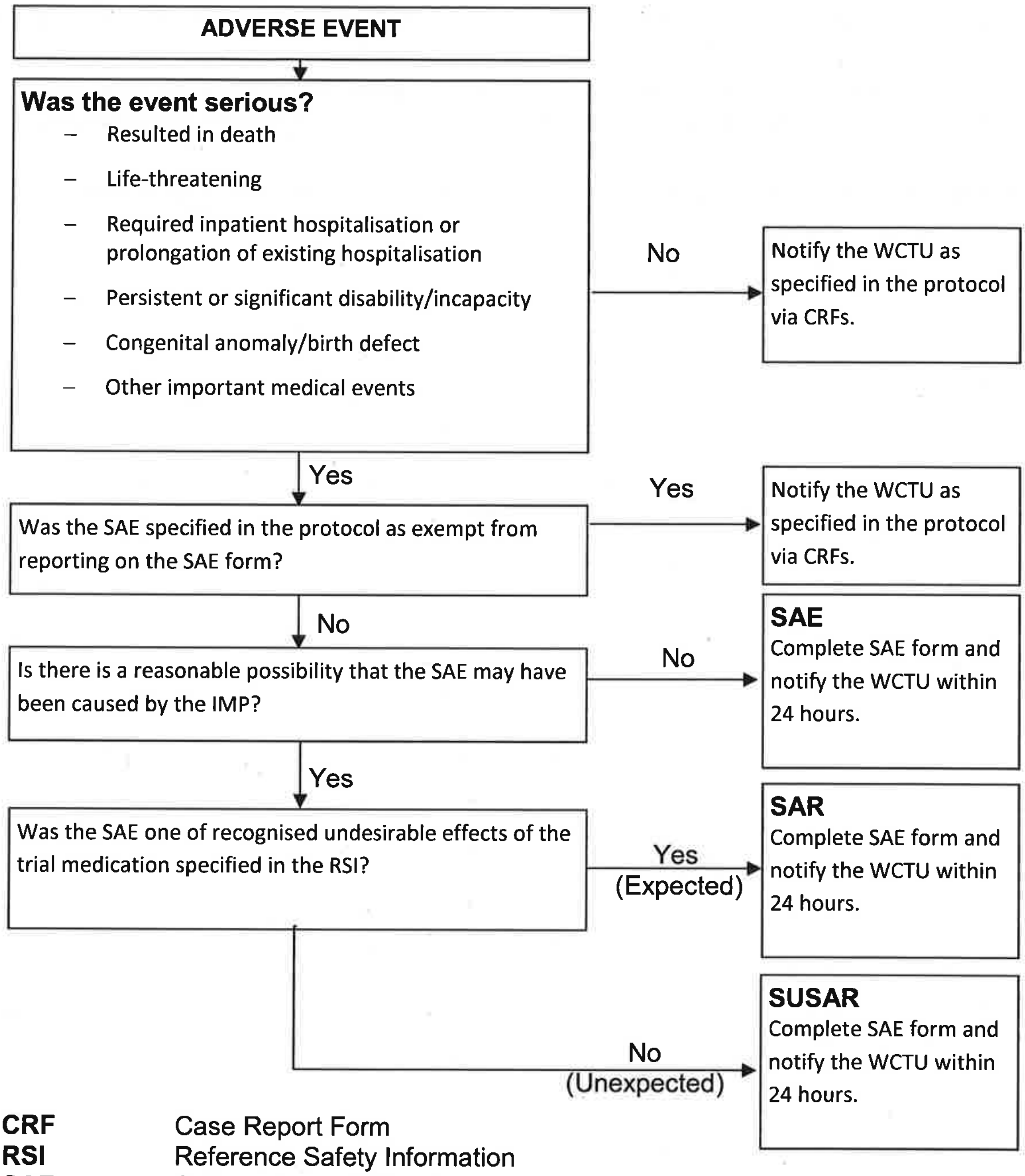

**CRF**	Case Report Form
**RSI**	Reference Safety Information
**SAE**	Serious Adverse Event
**SAR**	Serious Adverse Reaction
**SUSAR**	Suspected Unexpected Serious Adverse Reaction
**WCTU**	Wales Cancer Trials Unit

CONFIDENTIAL

This material is the property of the Wales Cancer Trials Unit. Do not disclose or use except as authorised.

13 Trial management

13.1 Trial committees and trial management

The conduct of the trial is being overseen by the following committees:

- The Trial Management Group (TMG) will be responsible for the day-to-day running of the trial and will meet approximately every six months. The TMG members will include the Co-Investigators, other active trial investigators, WCTU representatives, and specialist advisors (e.g. Pharmacist, Statistician, consumer representative).
- The data will be reviewed (approximately six monthly) by an **Independent Data Monitoring Committee** (IDMC), consisting of at least two Clinicians (not entering patients into the trial) and an independent Statistician. The IDMC will be asked to recommend whether the accumulated data from the trial, together with results from other relevant trials, justifies continuing recruitment of further patients. A decision to discontinue recruitment, in all patients or in selected subgroups, will be made only if the result is likely to convince a broad range of Clinicians including PIs in the trial and the general clinical community. If a decision is made to continue, the IDMC will advise on the frequency of future reviews of the data on the basis of accrual and event rates. The IDMC will make confidential recommendations to the Trial Steering Committee (TSC).
- The Trial Steering Committee (TSC) will be a committee of independent members that provides overall supervision of the trial. The role of the TSC is to act on behalf of the Sponsor, to provide overall supervision for the trial, to ensure that it is conducted in accordance with GCP, and to provide advice through its independent chairman. The TSC will review the recommendations from the IDMC and will decide on continuing or stopping the trial, or modifying the protocol. It will meet at least annually when it will consider each report of the IDMC, as well as results of other trials and new information which has arisen, and recommend appropriate action.

13.2 Monitoring

The clinical trial risk assessment has been used to determine the intensity and focus of monitoring activity in the NeoSCOPE trial. Moderate+ monitoring levels will be employed and are fully documented in the trial monitoring plan.

13.2.1 Site monitoring

If during central data monitoring, any concerns over participant safety and/or the quality and timeliness of the data from a particular site are raised, a triggered monitoring visit may be conducted. Slow CRF return, risk to participant safety, failure to complete data queries and protocol non-compliance may trigger a monitoring visit.

Investigators should agree to allow trial related monitoring, including audits and regulatory inspections, by providing direct access to source data/documents as required. Patient consent for this will be obtained.

13.2.2 Central monitoring and data queries

The top copy of each completed CRF should be returned to the WCTU for data entry within four weeks of the visit. The remaining copy is to be retained at the local centre. CRF pages and data received by the WCTU from participating trial centres will be checked for missing, illegible or unusual values (range checks) and consistency over time. If missing or questionable data are identified, a data query will be raised on a data clarification form. The data clarification form will be sent to the relevant participating site. The site shall be requested to answer the data query or correct data on the data clarification form. The CRF pages should not be altered. All answered data queries and corrections should be signed off and dated by member of staff listed on the delegation log at the relevant participating site. The completed data clarification form should be returned to the WCTU and a copy retained at the site along with the participants' CRFs. The WCTU shall send reminders for any overdue data. It is a centre's responsibility to submit complete and accurate data in a timely manner.

CONFIDENTIAL
This material is the property of the Wales Cancer Trials Unit. Do not disclose or use except as authorised.

13.3 Participant Withdrawal

In consenting to the trial, participants are consenting to trial treatment, trial follow-up and data collection. Participants may withdraw from the trial at any time. Patients may:

Level 1: Withdraw from trial treatment – participants stop trial treatment but remain in follow-up and continue to provide translational samples if applicable.

Level 2: Withdraw from the translational study – participants continue trial treatment and follow-up but do not provide translational samples

Level 3: Withdrawal from the translational study and trial treatment - participants stop trial treatment and do not continue providing translational samples but remain in follow-up.

Level 4: Complete withdraw from the trial – participants stop trial treatment, follow-up and any translational sample collection.

If a participant wishes to withdraw from trial treatment, participating sites should nevertheless explain the importance of remaining on trial follow up for the purposes of data capture only. Withdrawal for any reason requires a completed withdrawal CRF to be faxed to the WCTU with the hard copy to follow soon after. Participants do not have to give a reason for their withdrawal but sites should make a reasonable attempt to find out why.

A participant may withdraw, or be withdrawn, from trial treatment for the following reasons:

- Intolerance to treatment (including SAEs and toxicities)
- Participant choice
- Clinician's decision
- Non-concordance with protocol treatment
- Disease progression within or out of radiotherapy volume on post chemoradiotherapy CT scan that precludes surgery, at the discretion of the surgeon.

All patients with clinical disease progression should have a Progression CRF completed and then are withdrawn from the trial. In the case whereby a patient would like to completely withdraw from the trial (level 4), please arrange for the participant to sign the Participant Withdrawal Form.

Data and samples collected prior to participant withdrawal at any of the four levels indicated above will be collected and used for trial analysis by the WCTU. Participants who initially consented to be registered with the National Health Service Information Centre (NHSIC) or equivalent will remain on the system so that important research information on date and cause of death can be requested from NHSIC by the WCTU.

13.4 Lost to follow-up

If a participant is lost to follow up the WCTU will request that the PI contact the participant's GP to obtain information on the participant's status. Participants have the option to consent to NHS IC Flagging. This will entail completion of a separate consent form which will contain the participant name and will therefore be kept separate from the other data, and securely locked away. This will enable the WCTU to trace the participant cause and date of death.

CONFIDENTIAL
This material is the property of the Wales Cancer Trials Unit. Do not disclose or use except as authorised.

13.5 Protocol/GCP non-compliance

The Principal Investigator should report any non-compliance to the trial protocol or the conditions and principles of Good Clinical Practice to the WCTU as soon as they become aware of it.

The PI should report any non-compliance to the trial protocol or the conditions and principles of GCP to the WCTU using the WCTU Protocol/GCP non-compliance proforma as soon as they become aware of it.

13.6 The End of the Trial

The treatment phase will be followed by a follow-up period, which will continue for one year after the last participant completes protocol treatment.

The end of the trial is defined as the date of final data capture to meet the trial endpoints. In this case end of trial is defined as the last patient follow-up assessment 12 months post surgery.

13.7 Archiving

The TMF and ISF containing essential documents will be archived at an approved external storage facility for a minimum of 15 years. The WCTU will archive the TMF and TSFs on behalf of the Sponsor. The PI is responsible for archival of the ISF at site. Essential documents pertaining to the trial shall not be destroyed without permission from the Sponsor.

14 Statistical considerations

14.1 Randomisation

Randomisation will take place centrally after confirmation of eligibility by a telephone call to the WCTU. Participants will be randomised using minimisation with a random element. This will ensure balanced treatment allocation by a number of clinically important stratification factors. Randomisation will have an allocation ratio of 1:1. Also see section 7.0.

14.2 Outcome measures

Primary outcome measure

Efficacy: Pathological complete response rate (pCR) to be assessed in patients undergoing resection following neo-adjuvant treatment, as measured using standardised histological interpretation.

Secondary outcome measures

Feasibility: of recruiting to a pre-operative CRT trial in the UK as determined by recruitment within 18 months.
Toxicity: SAEs collected in real time, 30 day surgical morbidity/mortality, toxicities (CTCAE version 4.03) during treatment and late treatment toxicity at 6 and 12 months.
Efficacy: CRM positivity (as defined by the Royal College of Pathologist's guidelines) rate at resection; median, 3 and 5 year overall survival.

14.3 Sample size calculation

The sample size calculations are based on the maximum of two binomial random variables and follow the ideas of Dunnett (1984). The primary outcome of the study is rate of pathological complete response at resection. A response

rate of 15% would not be sufficiently large to warrant further investigation, while a response rate of 35% is considered worthwhile. Patients will be randomised 1:1 to each of the two treatment arms and the total sample size of the study is 76 (38 patients/arm). This design is based on a one-sided type I error of 10% and a power of 90% of achieving significance if patients on one novel treatment have a response rate of 35% while the second treatment has a response rate of 15%. This specification ensures that a power greater 90% is achieved if both treatments have a worthwhile effect of 35%. The study will seek to recruit a maximum total of 85 patients to account for a 10% drop-out rate before resection.

14.4 Statistical analyses

A full statistical analysis plan will be defined before the first interim analysis of the trial.

When 10 patients have completed treatment, i.e. 5 patients in either arm, a safety review will be performed. Full SAE and toxicity by arm will be presented to the IDMC for a recommendation as to whether or not to continue recruitment. Additionally, upon any event of 30 day postoperative mortality, the IDMC and TMG will be notified to discuss whether or not the trial should continue. The TMG will consider making a change to the radiotherapy protocol if necessary.

Upon completion of recruitment and follow up, the pCR rates and post operative mortality rates will be calculated by arm. The following rules will be used to decide how to proceed towards a Phase III trial:

- If fewer than 10 patients achieve a pCR to either treatment, no treatment is taken forward to a Phase III trial.
- If 10 or more patients achieve a pCR to treatment A but fewer than 10 patients achieve a pCR to treatment B, treatment A is taken forward to a Phase III trial.
- If 10 or more patients achieve a pCR to treatment B, but fewer than 10 patients achieve a pCR to treatment A, treatment B is taken forward to a Phase III trial.
- If both treatments have 10 or more patients achieve a pCR, the treatment with higher response rate is taken forward to a Phase III study provided the post operative mortality is less than 5% in both arms. If post operative mortality is > 5% for one of the treatments while the mortality is below 5% for the other, the treatment with the lower post-operative mortality is taken forward.
- If both arms show high pCR and similar mortality then toxicities will be used to help decide which arm to take forward to a future Phase III.

14.5 Sub-group analyses

No formal subgroup analyses are planned. However, if any treatment effect is found we will investigate whether it is consistent across participant subgroups (defined by all pre-treatment factors collected) although this analysis will be exploratory in nature. Exploratory analyses may be conducted to aid hypothesis generation if a phase III is subsequently developed.

15 T-NeoSCOPE: Sample collection for future translational research

Participants in the trial will be asked for additional optional consent to participate in translational research. Participants who decline can still take part in the rest of the study. All patients will be invited to provide blood and tissue samples as follows:

- **Pre-treatment diagnostic biopsy tissue including one or more formalin-fixed, paraffin-embedded blocks of tumour and one block of normal mucosa. In practice, most endoscopic biopsy samples will have been embedded into a single block.**
- **2x10ml EDTA blood samples to be taken within 14 days before the first day of cycle 1 chemotherapy.**

This material is the property of the Wales Cancer Trials Unit. Do not disclose or use except as authorised.

All samples will be stored at the Wales Cancer Bank (WCB) for future translational research. Postage and materials for sample collection will be provided by WCTU. Samples may be transported to research institutions nationally and possibly internationally upon request and after appropriate peer review of scientific hypothesis.

Whilst the samples from this small trial are unlikely to give any statistically significant answers with respect to prognostic or predictive biomarkers of response to therapy, they will form a unique collection which can further inform the research to be performed, and help generate hypotheses to be tested in a subsequent Phase III trial.

NeoSCOPE blood samples collected pre-treatment will be used for assessment of germline DNA markers related to toxicity and response to therapy.

NeoSCOPE tumour tissue will be used to assess potential biomarkers that may predict response to therapy, including immunohistochemical (IHC) and quantitative real-time reverse-transcription PCR (qRT-PCR) assessment of biomarkers of DNA repair, cell proliferation and cell cycling. Tissue will also be used for assessment of tumour cell density, gene amplification studies and gene expression profiling. The sequential tumour samples pre and post-treatment will allow assessment of early response (e.g. tumour regression in post-treatment resection specimens) and potential linkage of this to pre-treatment tumour characteristics.

15.1 Processing tumour samples

Once a patient has given consent, the samples in paraffin blocks from the diagnostic tissue samples described above should be requested from the respective pathology departments by the Research Nurse. They should be anonymised by blanking out the full name of the patient on the paraffin block and labelled with the patients' trial number, initials, date of birth, and time point of collection (baseline or post treatment) and posted in the jiffy bags provided by WCTU to WCB (address below). It is important that the pathology specimen number remains visible on the paraffin block. *See the NeoSCOPE Laboratory Guidelines for more detail.*

15.2 Processing blood samples

These should be labelled with the patient's trial number, initials, and date of birth using the freezer resistant labels provided. It is recommended that these are taken at the same time as the routine bloods if possible so that participants do not have to undergo any additional venepunctures.

For centres with an HTA licence and ability to store frozen samples, samples should be frozen and stored on the day of collection. Frozen samples should be stored until the end of the trial when WCTU will arrange their collection. They will be couriered on dry ice to the Wales Cancer Bank for storage

For centres without an HTA licence or without ability to store frozen samples, the samples should be posted to the Wales Cancer Bank on the same day that they are collected using the pre-paid safeboxes provided. Staff at centres should include a copy of the appropriate sample collection CRF (provided by WCTU) with each participant's sample sent to the Wales Cancer Bank. A copy of the sample collection CRF should also be sent by post to the WCTU within 4 weeks of sample collection along with the other CRFs for the patient (section 11.5). *See the NeoSCOPE Laboratory Guidelines for more detail.*

All samples should be sent to:
NeoSCOPE
c/o Wales Cancer Bank
Cardiff University, Dept of Cellular Pathology,
UHW, Heath Park, Cardiff CF14 4XW

CONFIDENTIAL
This material is the property of the Wales Cancer Trials Unit. Do not disclose or use except as authorised.

16 Publication policy

Data from all sites will be analysed together and published as soon as possible. Individual participating PIs may not publish data concerning their participants that are directly relevant to questions posed by the trial until the TMG has published its report. The TMG will form the basis of the writing committee and will advise on the nature of publications, subject to the Sponsor's requirements.

All publications should include a list of participating PIs, and if there are named authors, these should include the CI, Co-Investigators, Trial Manager and Statistician(s) involved in the trial. If there are no named authors then a writing committee will be identified.

CONFIDENTIAL
This material is the property of the Wales Cancer Trials Unit. Do not disclose or use except as authorised.

17 Ethical and regulatory considerations, and Informed Consent

17.1 Ethical approval

This protocol will be submitted to a Multi-centre Research Ethics Committee (MREC) that is legally "recognised" by the United Kingdom Ethics Committee Authority for review and approval. The approval of the MREC must be obtained before the start of a clinical trial or any trial procedures are conducted.

17.2 Clinical Trial Authorisation (CTA)

The trial is being performed under a Clinical Trial Authorisation (CTA) from the MHRA. The Clinical Trials Authorisation (CTA), the approval of the MHRA, must be obtained before the start of the trial in accordance with Part 3, Regulation 12 of The Medicines for Human Use (Clinical Trials) Regulations 2004 (SI2004/1031).

17.3 Regulatory Considerations

All substantial amendments to this Protocol must be approved by the MREC responsible for the study and MHRA, before the implementation of the amendments. Minor amendments will not require prior approval by the MREC and MHRA.

If the trial is temporarily halted it will not be recommenced without reference to the MREC responsible for the study and the MHRA.

The MREC and MHRA will be notified within 90 days of trial completion. If the trial is terminated early, the MREC and MHRA will be notified of this within 15 days.

A summary of the clinical trial report will be submitted to the MREC responsible for the study and MHRA within one year of the end of trial.

17.4 Research Governance approval

This trial protocol will be submitted through the Research Governance process of the host care organisation for review and approval. The Research Governance approval of the host care organisation must be obtained before recruitment of participants within that host care organisation.

17.5 Sponsorship

The NeoSCOPE trial is being sponsored by Velindre NHS Trust. Velindre NHS Trust shall be responsible for ensuring that the trial is performed in accordance with the following:

- The Medicines for Human Use (Clinical Trials) Regulations 2004 (SI2004/1031) and subsequent amendments
- Conditions and principles of Good Clinical Practice
- Declaration of Helsinki (1996)
- Research Governance Framework for Health and Social Care(Welsh Assembly Government 2009 and Department of Health 2nd July 2005)
- The Data Protection Act 1998
- The Human Tissue Act 2004
- The Ionising Radiation Medical Exposure Regulations (2000) (SI No. 1059) as amended
- Other regulatory requirements as appropriate

CONFIDENTIAL
This material is the property of the Wales Cancer Trials Unit. Do not disclose or use except as authorised.

The Sponsor has/will be delegating certain responsibilities to Cardiff University (WCTU), the Chief Investigators, Principal Investigators, host sites and other stakeholder organisations as appropriate in accordance with the relevant agreement that is informed by regulation and study type.

17.6 Indemnity

- Non-negligent harm: This trial is an academic, investigator-led and designed trial sponsored by Velindre NHS Trust and coordinated by the WCTU. The Chief Investigator, local Investigators and WCTU do not hold insurance against claims for compensation for injury caused by participation in a clinical trial and therefore cannot offer any non-negligent harm indemnity. The Association of the British Pharmaceutical Industry (ABPI) guidelines will not apply.
- Negligent harm: In accordance with Technical Note 12 Indemnity for Clinical Research for research Sponsored by a Welsh body, Welsh Risk Pool Services provides indemnity cover against successful negligence claims arising from the management and conduct of the study. Where NHS employees are responsible for the design of a study, indemnity cover will also be provided for negligent harm arising from the study design. Velindre NHS Trust does not accept liability for any breach in the other NHS Organisations duty of care, or any negligence on the part of employees of these NHS Organisations.

17.7 Data protection

The WCTU will act to preserve patient confidentiality and will not disclose or reproduce any information by which participants could be identified (except where participants are registered with the National Health Service Information Centre (NHSIC; formerly the Office for National Statistics) or traced via the NHS Central Register, which requires separate consent). Data will be stored in a secure manner and will be registered in accordance with the Data Protection Act 1998. The data custodian and the translational sample custodian for this trial is the Scientific Director of the WCTU.

17.8 Finance

NeoSCOPE is funded by Cancer Research UK (CRUK) (grant number CRUK/11/058). The WCTU is core funded by CRUK and these core resources will be used to support this trial. The trial is in the National Cancer Research Network (NCRN), the National Institute of Social Care and Health Research (NISCHR) portfolio and National Institute for Health (NIHR) portfolio. Local NCRN/WCTN/SCRN support should be available at each centre taking part to support entry of participants into this trial.

CONFIDENTIAL
This material is the property of the Wales Cancer Trials Unit. Do not disclose or use except as authorised.

18 References

Allum WH, Stenning SP, Bancewicz J, Clark PI, Langley RE (2009). Long term results of a randomized trial of surgery with or without preoperative chemotherapy in esophageal cancer. J Clin Oncol 27: 5062–5067.

Becker K, Mueller JD, Schulmacher C, Ott K, Fink U, Busch R, Böttcher K, Siewert JR, Höfler H (2003). Histomorphology and grading of regression in gastric carcinoma treated with neoadjuvant chemotherapy. Cancer. 2003 Oct 1;98(7):1521-30.

Button MR, Morgan CA, Croydon ES, Roberts SA, Crosby TD (2009). Study to determine adequate margins in radiotherapy planning for esophageal carcinoma by detailing patterns of recurrence after definitive chemoradiotherapy. Int J Radiat Oncol Biol Phys. 2009 Mar 1;73(3):818-23. Epub 2008 Aug 19.

CancerStats (2011): http://info.cancerresearchuk.org/cancerstats/types/oesophagus/
accessed on 7.7.11.

Chiarion-Sileni V, Innocente R, Cavina R, et al (2009).Multi-center phase II trial of chemoradiotherapy with 5-fluorouracil, leucovorin and oxaliplatin in locally advanced esophageal cancer. Cancer Chemother Pharmacol 2009;63:1111-1119.

Conroy T, Yataghène Y, Etienne PL, Michel P, Senellart H, Raoul JL, Mineur L, Rives M, Mirabel X, Lamezec B, Rio E, Le Prisé E, Peiffert D, Adenis A (2010). Phase II randomised trial of chemoradiotherapy with FOLFOX4 or cisplatin plus fluorouracil in oesophageal cancer. Br J Cancer. 2010 Oct 26;103(9):1349-55. Epub 2010 Oct 12.

Dexter SP, Sue-Ling H, McMahon MJ, Quirke P, Mapstone N, Martin IG (2001). Circumferential resection margin involvement: an independent predictor of survival following surgery for oesophageal cancer. Gut. 2001 May;48(5):667-70.

Dresner SM, Lamb PJ, Bennett MK et al. The pattern of metastatic lymph node dissemination from adenocarcinoma of the esophagogastric junction. Surgery 2001;129:103-9.).

Dunnett, CW. (1984). Selection of the best treatment in comparison to a control with an application to a medical trial. Pages 47–66 of: Santer, TJ, & Tamhane, AC (eds), Design of Experiments: Ranking and Selection. Marcel Dekker: New York.

Fiorica F, Di Bona D, Schepis F, et al (2004). Preoperative chemoradiotherapy for oesophageal cancer: a systematic review and meta-analysis. Gut 2004;53:925–30.

Gebski V, Burmeister B, Smithers BM, Foo K, Zalcberg J, Simes J, Australasian. Gastro-Intestinal Trials Group (2007). Survival benefits from neoadjuvant chemoradiotherapy or chemotherapy in oesophageal carcinoma: a metaanalysis. Lancet Oncol 2007;8:226–34.

Hawkins M, Brooks C, Hansen VN et al (2010). Cone-beam computed tomography derived adaptive radiotherapy for radical treatment of esophageal cancer. Int. J. radiation Oncolo Biol Phys. 2010. 77(2);378-383.

Hurt C , Nixon L , Griffiths G , Al-Mokhtar R, Gollins S , Staffurth J, Phillips C, Blazeby J and Crosby T (2011), SCOPE1: a randomised phase II/III multicentre clinical trial of definitive chemoradiation, with or without cetuximab, in carcinoma of the oesophagus. BMC Cancer 11:466.

Javle MM, Yang G, Nwogu CE, Wilding GE, O'Malley L, Vinjamaram S, Schiff MD, Nava HR, LeVea C, Clark KR, Prey JD, Smith PF, Pendyala L (2009). Capecitabine, oxaliplatin and radiotherapy: a phase IB neoadjuvant study for esophageal cancer with gene expression analysis. Cancer Invest. 2009 Feb;27(2):193-200.

Khan OA, Cruttenden-Wood D, Toh SK (2010). Is an involved circumferential resection margin following oesphagectomy for cancer an important prognostic indicator? Interact Cardiovasc Thorac Surg. 2010 Nov;11(5):645-8. Epub 2010 Aug 24. Review.

CONFIDENTIAL
This material is the property of the Wales Cancer Trials Unit. Do not disclose or use except as authorised.

Khushalani NI, Leichman CG, Proulx G, Nava H, Bodnar L, Klippenstein D, Litwin A, Smith J, Nava E, Pendyala L, Smith P, Greco W, Berdzik J, Douglass H, Leichman L (2002). Oxaliplatin in combination with protracted-infusion fluorouracil and radiation: report of a clinical trial for patients with esophageal cancer. J Clin Oncol. 2002 Jun 15;20(12):2844-50.

Kranzfelder, Schuster, Geinitz, Friess, Büchler (2011). Meta-analysis of neoadjuvant treatment modalities and definitive non-surgical therapy for oesophageal squamous cell cancer. British Journal of Surgery 98 (6): 768–783.

Macmanus, M., U. Nestle, et al. (2009). "Use of PET and PET/CT for Radiation Therapy Planning: IAEA expert report 2006–2007." Radiotherapy and Oncology 91: 85-94.

Malthaner RA, Wong RK, Rumble RB, Zuraw L et al (2004). Neo-adjuvant or adjuvant therapy for resectable oesophageal cancer: a systematic review and meta-analysis. BMC Med 2:35.

Mandard AM, Dalibard F, Mandard JC, Marnay J, Henry-Amar M, Petiot JF, Roussel A, Jacob JH, Segol P, Samama G, Ollivier J-M, Bonvalot SB, Gignoux M (1994). Pathologic assessment of tumor regression after preoperative chemoradiotherapy of esophageal carcinoma. Clinicopathologic correlations. Cancer, 73:2680-2686.

Martinger O, Gerber E, Bernstein Z, et al (2009). EORTC-ROG expert opinion: Raotherapy volume and treatment guidelines for neopadjuvant radiation of adenocarcinomas of gastroesophageal junction and the stomach. Radiother Oncol. 2009.

Medical Research Council Oesophageal Cancer Working Group (2002). Surgical resection with or without preoperative chemotherapy in oesophageal cancer: a randomised controlled trial. Lancet. 2002 May 18;359(9319):1727-33.

Muijs,C T, Beukema J C, Pruim J, Mul V E, Groen H, Plukker J Th, Langendijk J A. (2010). "A systematic review on the role of FDG-PET/CT in tumour delineation and radiotherapy planning in patients with esophageal cancer". Radiotherapy and Oncology 97:165-171.

O'Connor BM, Chadha MK, Pande A, Lombardo JC, Nwogu CE, Nava HR, Yang G, Javle MM (2007). Concurrent oxaliplatin, 5-fluorouracil, and radiotherapy in the treatment of locally advanced esophageal carcinoma. Cancer J. 2007 Mar-Apr;13(2):119-24.

Palser T, Cromwell D, Van der Meulen J et al (2010). The National Oesophago-Gastric Cancer Audit. An audit of the care received by people with Oesophago-gastric Cancer in England and Wales. Third Annual Report 2010. London, NHS Information Centre.

Stahl, Walz, Stuschke, Lehmann, Meyer, Riera-Knorrenschild, Langer, Engenhart-Cabillic, Bitzer, Königsrainer, Budach, Wilke (2009). Phase III Comparison of Preoperative Chemotherapy Compared With Chemoradiotherapy in Patients With Locally Advanced Adenocarcinoma of the Esophagogastric Junction. JCO Feb 20, 2009:851-856.

Thirion, Michiels, Le Maître, Tierney (2007). Individual patient data-based meta-analysis assessing pre-operative chemotherapy in resectable oesophageal carcinoma. Journal of Clinical Oncology, 2007 ASCO Annual Meeting Proceedings Part I. Vol 25, No. 18S (June 20 Supplement), 2007: 4512].

Thukral AD, Metz J, Hwang WT, O'Dwyer P, Plastaras J, Both S, Ad VB (2010). Toxicity data for preoperative concurrent chemoradiotherapy with oxaliplatin and continuous infusion 5-fluorouracil for locally advanced esophageal cancer. Dis Esophagus. 2010 Dec 10. doi: 10.1111/j.1442-2050.2010.01145.x. [Epub ahead of print].

Urschel JD, Vasan H (2003). A meta-analysis of randomized controlled trials that compared neoadjuvant chemoradiation and surgery to surgery alone for resectable esophageal cancer. Am J Surg 185(6):538-43.

Van Hagen P, Hulshof MC, van Lanschot JJ, Steyerberg EW, van Berge Henegouwen MI, Wijnhoven BP, Richel DJ, Nieuwenhuijzen GA, Hospers GA,Bonenkamp JJ, Cuesta MA, Blaisse RJ, Busch OR, ten Kate FJ, Creemers GJ, Punt CJ, Plukker JT, Verheul HM, Spillenaar Bilgen EJ, van Dekken H, van der Sangen MJ, Rozema T, Biermann K, Beukema JC, Piet AH, van Rij CM, Reinders JG, Tilanus HW, van der Gaast A; CROSS Group, (2012). Preoperative chemoradiotherapy for esophageal or junctional cancer. N Engl J Med. 2012 May 31;366(22):2074-84.

Wahba HA, El-Hadaad HA, Abd-Ellatif EA (2011). Neoadjuvant concurrent chemoradiotherapy with capecitabine and oxaliplatin in patients with locally advanced esophegeal cancer. Med Oncol. 2011 Jun 26. [Epub ahead of print].

Wills L, Millin A, Paterson J, Crosby T, Staffurth J (2009). The effect of planning algorithms in oesophageal radiotherapy in the context of the SCOPE 1 trial. Radiother Oncol. 2009 Dec;93(3):462-7. Epub 2009 Sep 24.

Yamashita H, Kida S, Sakumi A, et al (2011). Four-dimensional measurement of the displacement of internal fiducial markers during 320-multislice computed tomography scanning of thoracic esophageal cancer. Int. J Radiation Oncol Biol Phys. 2011. 79(2);588-595.

19 Appendices

APPENDIX 1: A guide to performing causality assessments

The following factors should be considered when deciding if there is a "reasonable possibility" that an SAE may have been caused by the drug.

- Time course. Exposure to suspect drug. Has the subject actually received the suspect drug? Did the SAE occur in a reasonable temporal relationship to the administration of the suspect drug?
- Consistency with known drug profile. Was the AE consistent with the previous knowledge of the suspect drug (pharmacology and toxicology) or drugs of the same pharmacological class? Or could the SAE be anticipated from its pharmacological properties?
- Dechallenge experience. Did the SAE resolve or improve on stopping or reducing the dose of the suspect drug?
- No alternative cause. The SAE cannot be reasonably explained by another aetiology such as the underlying disease, other drugs, other host or environmental factors.
- Laboratory tests. A specific laboratory investigation (if performed) has confirmed the relationship?
- A "reasonable possibility" could be considered to exist for an SAE where one or more of these factors exist.

In contrast there would not be a "reasonable possibility" of causality if none of the above criteria apply or where there is evidence of exposure and a reasonable time course but any dechallenge (if performed) is negative or ambiguous or there is another more likely cause of the SAE.

In difficult cases other factors should be considered such as:
- Is this a recognised feature of overdose of the drug?
- Is there a known mechanism?

Ambiguous cases should be considered as being a "reasonable possibility" of causal relationship unless further evidence becomes available to refute this. Causal relationship in cases where the disease under study has deteriorated due to lack of effect should be classified as no reasonable possibility.

APPENDIX 2: WHO Performance status

0 – Asymptomatic (Fully active, able to carry on all predisease activities without restriction)

1 – Symptomatic but completely ambulatory (Restricted in physically strenuous activity but ambulatory and able to carry out work of a light or sedentary nature. For example, light housework, office work)

2 – Symptomatic, < 50% in bed during the day (Ambulatory and capable of all self care but unable to carry out any work activities. Up and about more than 50% of waking hours)

3 – Symptomatic, > 50% in bed, but not bedbound (Capable of only limited self-care, confined to bed or chair 50% or more of waking hours)

4 – Bedbound (Completely disabled. Cannot carry on any self-care. Totally confined to bed or chair)

5 – Death

APPENDIX 3: Renal function - Cockroft & Gault formula

GFR for males = $\frac{1.23 \times [140\text{-age}] \times \text{weight (kg)}}{\text{Serum creatinine (mol/l)}}$

GFR for females = $\frac{1.05 \times [140\text{-age}] \times \text{weight (kg)}}{\text{Serum creatinine (mol/l)}}$

APPENDIX 4: Briefing for the NeoSCOPE trial pathologist

By Dr Heike Grabsch MD PhD FRCPath
Department of Cellular Pathology, St James's Institute of Oncology, Leeds Teaching Hospitals NHS Trust, Leeds LS9 7TF

A central review of the pathology report(s) and all H&E slides from the pre-treatment biopsy and the resection specimen of all patients recruited into the NeoSCOPE trial is part of the quality assurance of pathology and surgery within the NeoSCOPE trial. The central pathology review will be performed after the pathology review by local MDT and will be led by Dr H Grabsch, Consultant Histopathologist specialised in GI pathology at the Leeds Teaching Hospitals NHS Trust. All available images of the macroscopic specimen will be uploaded to a central server in Leeds. H&E slides of the biopsy and the resection specimen will be scanned and stored in the same server location. Pathologists and/or investigators who are interested in looking at the scanned slides and macroscopic specimen images should contact Dr H Grabsch in the first instance (email h.i.grabsch@leeds.ac.uk).

Results of the central pathology review will be published and the lead GI pathologist of all participating centres will be acknowledged. It is not expected that there will be any major discrepancies between the local pathologist and the central review. However, in the unlikely case there are major discrepancies between the local pathologist and the central pathology review, the central review pathologist will contact the local pathologist and discuss the findings. If both agree that changes need to be made to the original pathology report, the local pathologist will be asked to inform the recruiting clinician and/or the local Multi-Disciplinary Team Meeting as appropriate.

As the results from the histopathology assessment of the resection specimen has no impact on patient's treatment in the NeoSCOPE trial, the slides for central pathology review should be shipped to Leeds approximately 4 weeks after surgery and only after the cases has been discussed at the local MDT. Slides will be returned as soon as possible. Clinicians/pathologists do not need to wait for the result of the central pathology review in order to make any clinical decision.

Table of contents

1. Clinical information
2. Specimen preparation before dissection
3. Macroscopic examination
4. Tissue sampling from the resected specimen within the NeoSCOPE trial
5. Microscopic examination
6. Central pathology review

1. Clinical information

1.1 Participant initials, trial ID number, date of birth and pathology specimen number are data necessary to enable linking of the pathology data to the participant's clinical data, held at Wales Cancer Trials Unit. These data need to be recorded on the NeoSCOPE Trial Pathology Reporting Form which should have been forwarded to the local pathology department by the local clinical trials research nurse prior to cut-up of the specimen. If questions arise from the pathology report and for future reference, it would be helpful to have the name of the reporting pathologist recorded on the same form.

1.2 The histopathology request form which is submitted with the resection specimen to the pathology department should ideally indicate clearly that the patient is taking part in the NeoSCOPE trial to alert the pathology department. Specimen labels will be provided by the trial unit to the trial surgeon.

The resection specimen received as part of the NeoSCOPE trial will always be a post-chemoradiotherapy specimen and thus the macroscopic tumour may be very difficult to locate in the specimen. Ideally, the surgeon should indicate the tumour location (junctional cancer or approximate distance from the gastro-oesophageal junction) on the histopathology request form.

CONFIDENTIAL
This material is the property of the Wales Cancer Trials Unit. Do not disclose or use except as authorised.

1.3 It is necessary to record the pathology specimen number of the pre-treatment biopsy plus the name of the hospital if the biopsy was performed outside the trial centre and the pathology specimen number of the resection specimen on the NeoSCOPE Trial Pathology Reporting Form to facilitate the planned central pathology review of all cases.

2. Specimen preparation before dissection

2.1 Ideally, for the benefit of the pathologist, the surgeon should attach orientation sutures to the resection specimen whilst the specimen is still in situ. It is suggested to attach a long suture onto the anterior surface of the stomach and a long suture onto the anterior surface of the oesophagus and a short suture to the right side of the oesophagus.

It is expected that the resection specimen will be submitted to the pathology laboratory fresh (e.g. unfixed, not in formalin) and 'intact' e.g. with all peri-oesophageal and peri-gastric tissue attached. Tumour tissue from these specimens should not be taken for biobanking as the whole tumour/tumour bed will need to histologically assessed for treatment response.

As part of the NeoSCOPE trial protocol, surgeons are not allowed to dissect lymph nodes off the specimen surrounding the tumour (approximately 2cm above and below the presumed distal and proximal edge of the tumour). Dissecting off nodes would destroy the circumferential resection margin and the pleural surface and hence would significantly interfere with the pathology assessment planned within this trial. However, should the surgeon wish to do so, it would be permissable to dissect the nodes from the peri-gastric fat provided that there is no evidence of direct tumour extension into this fat and dissected nodes a identified and labelled appropriately.

CONFIDENTIAL

This material is the property of the Wales Cancer Trials Unit. Do not disclose or use except as authorised.

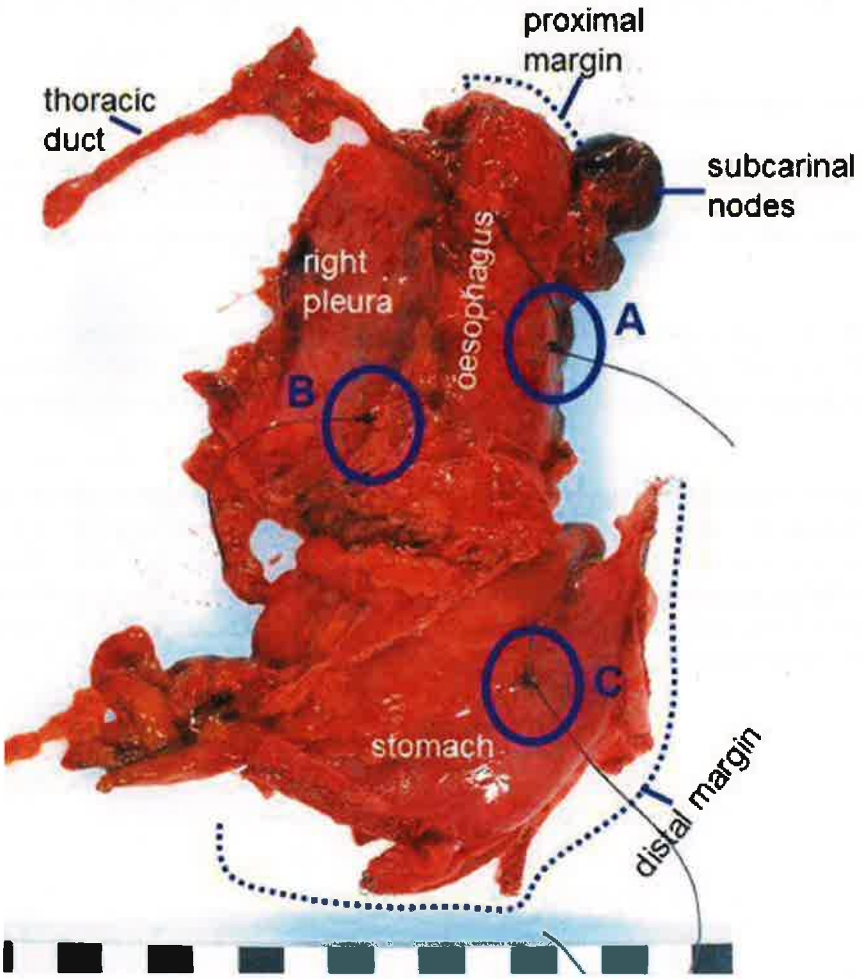

Figure 1. Anterior view of a fresh (unfixed) Ivor Lewis oesophagogastrectomy specimen with attached peri-oeosophageal, peri-junctional and peri-gastric tissue.
Orientation sutures were attached by the surgeon, see blue circles:
A = anterior oesophagus, B= right side oeosophagus, C= anterior stomach.

2.2 The specimen should be delivered to the pathology department fresh (unfixed) as soon as possible after resection. Should the pathology laboratory already been closed for the day, the specimen should be placed into a bucket **without formalin** and stored in a suitable fridge until delivery to the pathology laboratory in the next morning. Immersion of the specimen into formalin in theatre will lead to significant shrinkage and distortion of the specimen making measurements and assessment of the location of a positive CRM unreliable. If it is absolutely not possible to deliver the specimen fresh, the specimen should be pinned onto a paperboard by the surgeon before putting it into a sufficiently large amount of fixative in order to preserve the shape and length of specimen (Figure 2).

If possible, the resection specimen should be photographed before fixation and before opening documenting the anterior and posterior aspect of the specimen (Figure 1). Please always include a scale (ruler) in any photograph you take.

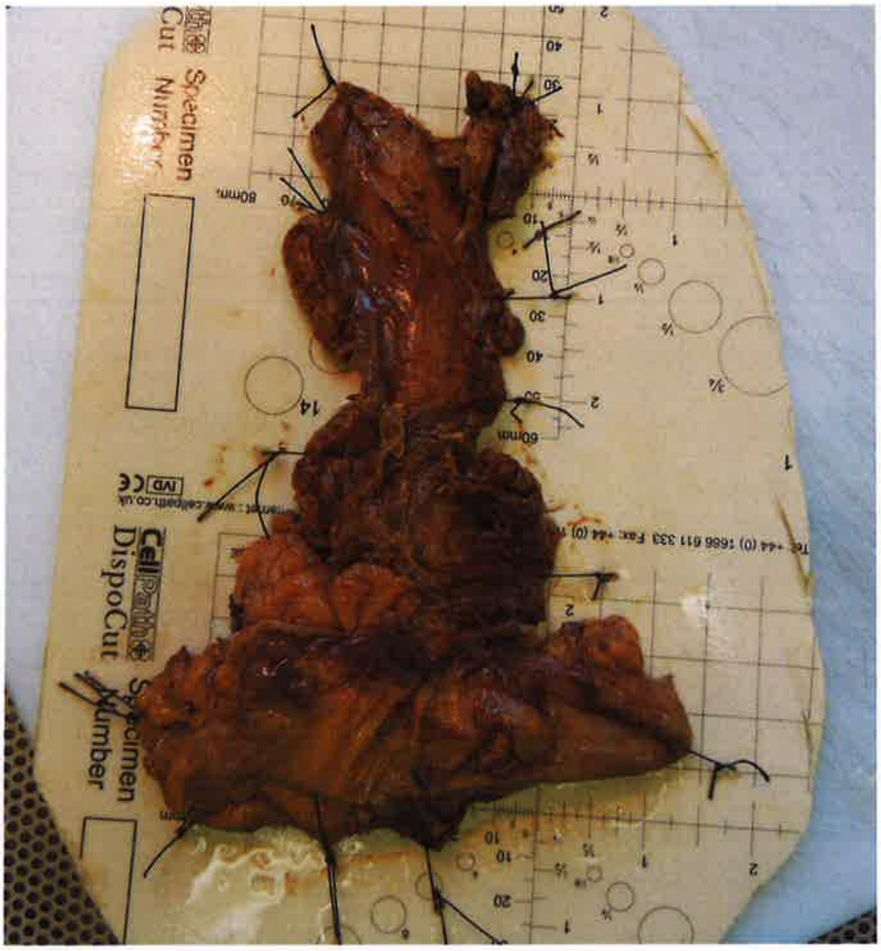

Figure 2: Anterior view of an oesophagogastrectomy specimen which has been attached with sutures to a paperboard by the surgeon and then immersed into a large bucket of formalin in order to preserve the shape and length of specimen.

2.3 The stomach is opened along the distal gastric resection margin which will most commonly be stapled. As part of the trial protocol, the oesophagus itself is left intact, gently stretched to approximately 12 to 15cm length and the specimen is pinned out onto a board for fixation. Ideally, the oesophageal tube should not be opened longitudinally before or after fixation. If local practice cannot be adapted, it would be acceptable to open the oesophageal tube longitudinally AFTER inking of the circumferential margin. The specimen should be left on the board in plenty of formalin for at least 48 hours (Figure 3).

CONFIDENTIAL

This material is the property of the Wales Cancer Trials Unit. Do not disclose or use except as authorised.

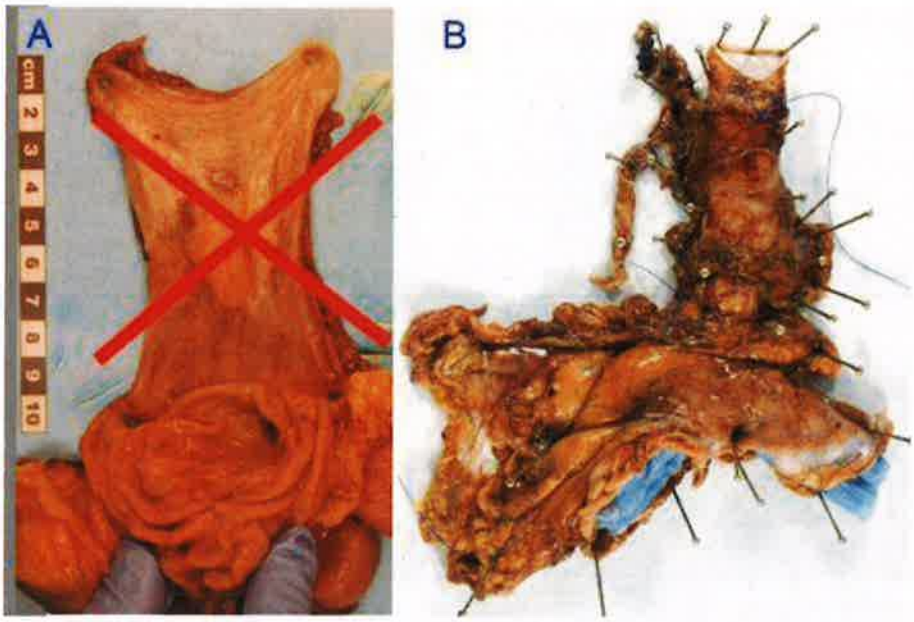

Figure 3. Specimen after being pinned onto a board and fixed for 48 hours.
A: **The pathologist should not open the oesophageal tube longitudinally** as shown here. However, if local practice cannot be adapted to this approach, it would be acceptable to open the oesophageal tube longitudinally provided that the circumferential margin was inked before opening.

B: Example of how the specimen looks when the oesophageal tube is left intact prior to fixation and the specimen was pinned onto a board prior to 48 h fixation. Blue paper has been placed into the stomach to help with fixation in a 'natural' shape.

2.4 After 48h fixation, the specimen is taken off the board and photographed from anterior and posterior including a scale. The specimen orientation provided by the surgeon is then 'transcribed' into a colour code applied to the circumferential margin of the specimen. For example, the right side could be painted in red, anterior in yellow, left in green and posterior in black (Figure 4). The colour code used should be included in the macroscopic specimen description.

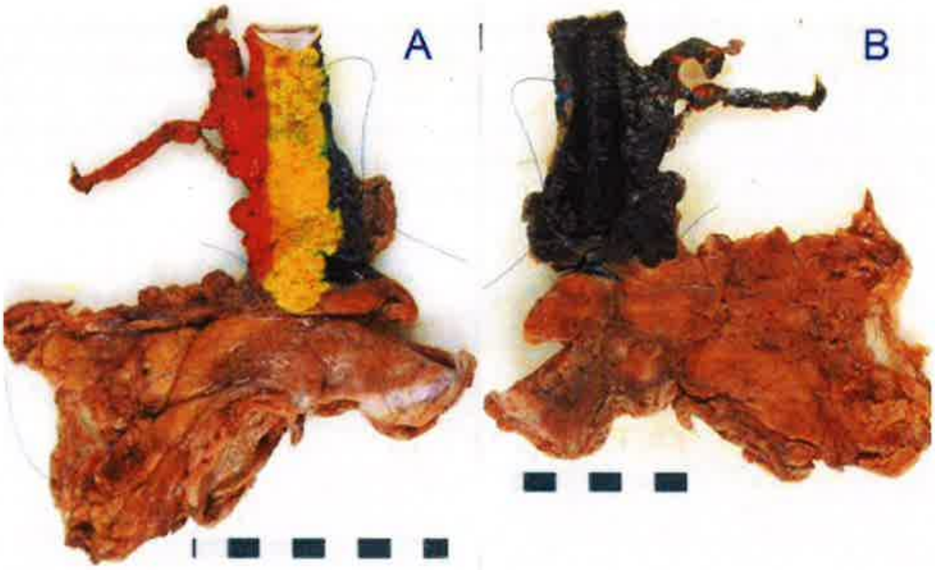

Figure 4. 'Colour coding' of the fixed resection specimen.
A: View from anterior. Right side in red, anterior in yellow, green on the left side.
B: View from posterior. Posterior surface inked black.
Stomach not inked.

This material is the property of the Wales Cancer Trials Unit. Do not disclose or use except as authorised.

2.5 The dimensions of the specimen are measured including the length of the oesophagus, length of the greater curve, lesser curve, distal resection margin, shortest distance from the gastro-oesophageal junction to the distal resection margin. Thereafter, the oesophageal tube and the gastro-oesophageal junction are horizontally sliced at approximately 4mm intervals. All macroscopic cross-sections should be photographed including a scale in the picture (Figure 5). If it is impossible to take colour photographs, then the specimen and the cross-sections can be placed onto a photocopier or flat bed scanner in order to generate macroscopic images. If this is also not possible, some diagrammatic drawings of the specimen and location of the tumour should be made.

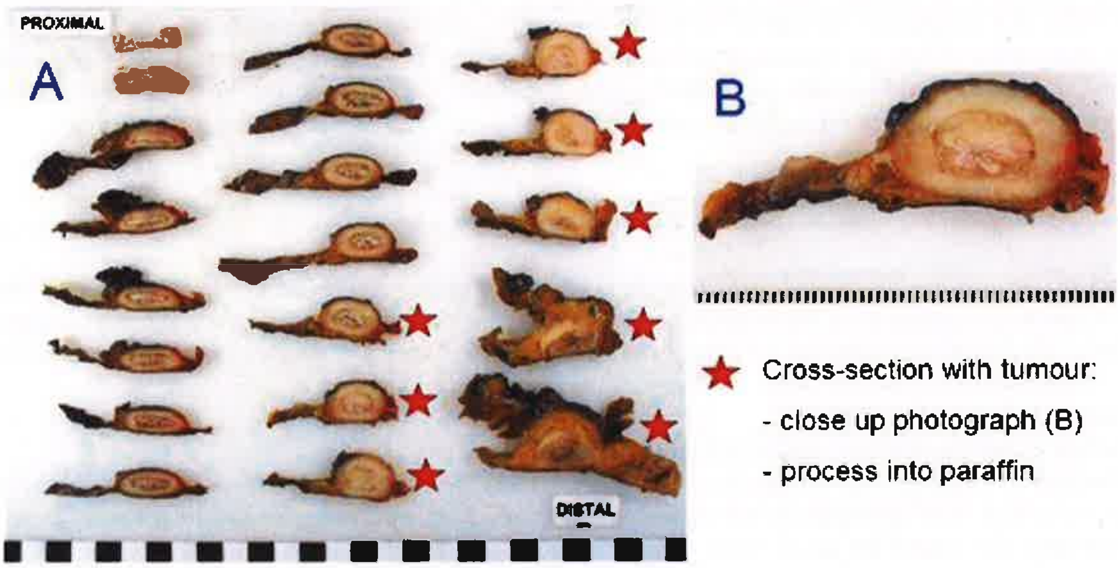

Figure 5: Specimen photographing after cross-sectioning
A: The specimen is cross-sectioned at 4mm intervals. Cross-sections are laid out onto a board and an overview photograph with all cross-sections including a ruler/scale is taken.
B: All cross-sections showing tumour/fibrosis macroscopically (in the example highlighted with a red star in A) are photographed as 'close ups', again including a ruler.

2.6 All photographs taken should include metric scales to allow calibration. Images should not contain any direct patient identifiers (e.g. name, NHS number) but should be identifiable by trial name (NeoSCOPE), pathology specimen number, patient's initials, date of birth, and importantly the patient's trial number. All images should be of highest possible resolution, copied to CD-ROM and sent to:

Dr Heike Grabsch
NeoSCOPE Trial
Department of
Cellular Pathology
Leeds Teaching Hospitals NHS Trust
St James's Institute of Oncology
Bexley Wing, Level 5
Beckett Street
Leeds LS9 7TF

3. Macroscopic examination

3.1 As the length of the oesophagus may shrink substantially after fixation, it is important to indicate whether measurements were done before or after fixation and whether the specimen was pinned out or not. Preferably, measurements are done after the specimen has been fixed while pinned out.

3.2 If a tumour is visible macroscopically, the tumour type should be described referring to the cross-sectional observation as polypoid/protruding, ulcerative, and diffusely infiltrative. If there is no obvious tumour mass macroscopically please choose the category 'no obvious tumour mass'.

Macroscopic measurements (see NeoSCOPE Pathology Case Report Form) of maximal longitudinal tumour dimension, maximal transverse tumour dimension and distance of tumour edge to the distal, proximal and circumferential margin should be performed AFTER fixation and recorded in mm. The same measurements should be performed when only fibrosis/necrosis is seen to establish the size and longitudinal location of the tumour before treatment.

3.3 If possible, the location of the tumour/area with evidence of tumour regression (fibrosis, necrosis) within the circumference (e.g. anterior left, anterior left, posterior right, posterior left, whole circumference) should be recorded. The distance of the tumour centre to the gastro-oesophageal junction defined as the proximal end of the rugal folds should be measured to confirm (or not) the pre-treatment Siewert type. According to the NeoSCOPE trial selection criteria for patients, there should be no patient in the NeoSCOPE trial with a tumour which is located entirely in the proximal stomach.

4. Tissue sampling from the resected specimen within the NeoSCOPE trial

The resected specimen should be well fixed prior to cutting. The oesophageal tube including the gastro-oesophageal junction is sliced perpendicular to the longitudinal axis of the specimen at 4mm intervals, laid out on a flat surface in good light for photographing (see Figure 5 above) and macroscopic inspection of depth of invasion and tumour extent.

The following blocks should be taken:

4.1 Proximal and distal resection margin (cut ends)

If the specimen arrives with a separate anastomotic donut specimen, the donut with the squamous epithelium represents the 'true' proximal resection margin and should be embedded completely. Please note, that the sometimes also submitted donut lined by gastric epithelium is not a 'true' margin and does not need to be processed.

If there is no donut, a section taken parallel to the proximal margin should be embedded. The distal (gastric) resection margin can be relatively long (>10cm). Unless the tumour is located within 10mm or less from this margin, two or three cassettes with 'random' tissue taken parallel to the stapled distal resection margin will suffice.

If the distance of the tumour edge/area of regression to the resection margin appears to be 10mm or less, then several blocks should be taken perpendicular to the margin to include margin and tumour edge/area of regression to allow accurate microscopically measurement of the distance.

4.2 Circumferential resection margin (CRM)

Any non-serosa (peritoneum, pleura, pericard) covered surface is considered a circumferential resection margin. As part of this trial, we aim to record the location of the tumour at the CRM (e.g. whether the CRM is involved anterior, right, left or posterior) and the length of CRM involvement by tumour/regression.

4.3 If tumour is macroscopically visible

In order to determine the percentage area of residual tumour as accurate as possible within the NeoSCOPE patients, the whole tumour bed including areas with macroscopically visible regression should be processed into paraffin. The cut up pathologist should record in the macroscopic description whether the macroscopically identifiable tumour bed has been completely processed into paraffin. If the tumour bed has not been processed completely, it should be recorded how many blocks have been taken from the tumour bed and how many cross-sections with tumour/tumour regression have not been processed. Ideally, if possible locally, the macroscopic slices should be processed as whole mounts into 'big blocks' (Figure 6) to allow assessment of deepest penetration, circumferential resection margin and serosa involvement (pleura for mid-oesophageal tumours and peritoneum for junctional cancer) as appropriate. Big blocks preserve the regional anatomy and make the assessment of the relevant pathology and comparison to clinical imaging much easier. If 'big blocks' are not possible locally, 'composite' blocks using regular sized cassettes would be acceptable.

CONFIDENTIAL
This material is the property of the Wales Cancer Trials Unit. Do not disclose or use except as authorised.

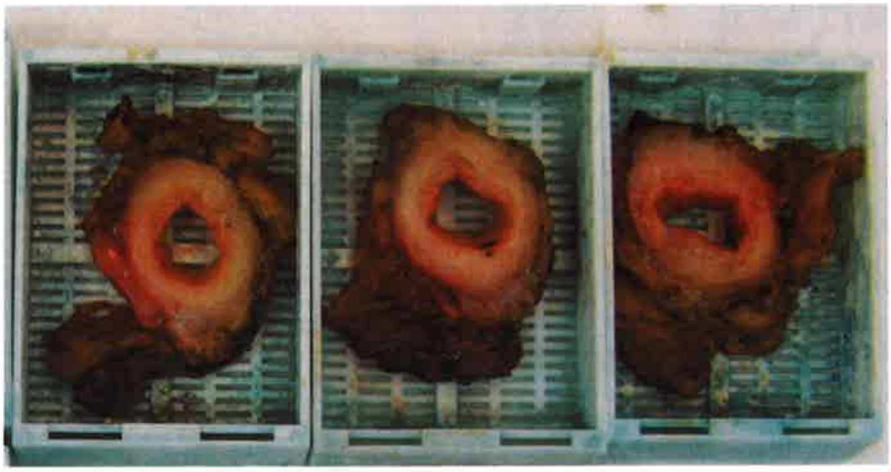

Figure 6: Processing of whole cross-sections into big blocks.

4.4 If there is no macroscopically visible tumour

The primary endpoint of this trial is the rate of complete pathological response. Ideally, the whole tumour bed should always be processed into paraffin whether there is visible tumour or not. If no tumour can be seen on macroscopy but an area of fibrosis can be identified, the pathologist should block the whole area of the presumed tumour bed plus one or two sections on each side and assess under the microscope. If there is no microscopic tumour and no area of regression, then the whole length of the oesophagus plus the gastro-oesophageal junction may need to be processed into paraffin unless the pathologist is provided with the pre-treatment tumour location by the clinicial team.

Ideally, cross-sections are left intact and processed into big blocks (figure 6) to preserve anatomical relationships. Macroscopic slices should be counted for example from proximal to distal and a cross-reference between macro slice number and block number should be recorded in the macroscopic description. This record will allow to determine the location of the tumour should some tumour cells be seen on microscopy. If there is no viable tumour on the first cut level, three further levels should be cut into all blocks with tumour regression before diagnosing a complete pathological response (ypT0). For tumour regression grading, see 5.8 below.

4.5 If there are multiple primary cancers

Describe and embed all cancers separately.

4.6 Non-cancerous mucosa

One or two blocks from random uninvolved gastric body mucosa should be embedded in addition to the distal resection margin to assess the background gastric mucosa. Two blocks from oesophageal mucosa next to the tumour should be embedded to assess whether there is Barrett's mucosa or dysplasia in the case of adenocarcinoma. One or two random blocks with normal squamous lined oesophagus should be taken in addition to the proximal margin. The blocks from the normal tissue will be useful to assess the effect of chemoradiation on non neoplastic tissue.

4.7 Lymph nodes

All lymph nodes which are dissected off the main specimen should be identified as peri-oesophageal nodes, peri-junctional nodes and peri-gastric nodes in the macroscopic description (block list) and processed into separate blocks to allow lymph node assessment by location. This will allow the assessment of the efficacy of chemoradiotherapy on lymph nodes in different locations. It should be recorded whether a cassette contains a single node or multiple nodes to allow counting. If possible, nodes should always be halved. Larger nodes may require more slices and more than one cassette to enable complete embedding.

If there is fibrosis/evidence of tumour regression but no viable tumour in the lymph node, three further levels should be cut before diagnosing ypN0.

CONFIDENTIAL
This material is the property of the Wales Cancer Trials Unit. Do not disclose or use except as authorised.

5. Microscopic examination (see also NeoSCOPE Trial Pathology Reporting Form in the CRFs)

Please note that within the NeoSCOPE Trial, all cases will be staged according to the TNM classification for oesophageal cancer 7th Edition.

Tumour related parameters

5.1 The ***histological type of tumour*** is recorded as adenocarcinoma, squamous cell carcinoma or 'other – please specify'. There should only be patients with adenocarcinoma in this trial. However, morphology may change due to chemoradiotherapy and tumours may become undifferentiated.

5.2 The resection specimens are all specimens after neoadjuvant chemoradiotherapy. Therefore, the ***grade of tumour differentiation*** is recorded by predominant area and not by worst area using a three tier system (well/moderate/poor).

5.3 ***Depth of local invasion (T category)*** is assessed according to the 7th edition of the TNM staging system. Serosa involvement (peritoneum or pleura) is defined according to Shepherd N et al as 'tumour cells have penetrated through the serosal membrane or are seen on the surface enmeshed in a fibrinous inflammatory reaction'. Levels may be necessary if serosal involvement was suspected macroscopically but cannot be seen on the first cut haematoxylin/eosin section. The deepest location of viable tumour cells in the wall determines the T category. Necrotic debris, fibrosis or mucin lakes without viable tumour cells are not considered in the T category. Please note that the location of viable tumour cells located within lymphatic or blood vessels is also not relevant for the T category.

5.4 All ***resection margins*** (longitudinal and circumferential) require histological exclusion of tumour involvement.
Any resection margin is considered to be microscopically involved (classified as ypR1), if viable tumour (main tumour mass OR soft tissue deposits OR lymph node metastasis OR tumour cells in vessels) lies within 1mm from the margin.

Accurate measurement of the minimum distance between tumour and resection margin should be attempted by microscopy on the haematoxylin/eosin stained slide using the Vernier scale on the microscope stage.

If there is circumferential resection margin involvement, the location (anterior, posterior, right, left) and it's extent (length and width) should be recorded if possible.

5.5 ***Lymphovascular invasion*** is recorded as present or absent in the NeoSCOPE pathology form. If possible, the pathologist should record invasion of lymphatic vessels and blood vessel separately.

5.6 The presence or absence of ***Barrett's metaplasia*** adjacent to the tumour should be noted. Similarly, the presence or absence of squamous or glandular dysplasia should be recorded.

5.7 The total number of lymph nodes and the total number of positive ***lymph nodes*** need to be recorded. As described above, the pathologist should distinguish between peri-oesophageal, peri-junctional and peri-gastric lymph nodes as indicated on the NeoSCOPE Trial Pathology Case Report Form.

5.8 ***Distant metastases*** (liver, lung, peritoneal seedlings) can only be included in the pathology report as ypM1 if confirmed histologically.

5.9 As all resection specimens within the NeoSCOPE trial are post-chemoradiotherapy specimens, the histological ***tumour regression grade*** needs to be assessed. During the NeoSCOPE trial, the local pathologist is asked to provide two regression grades for the primary cancer
a) an estimation of the percentage of viable tumour in relation to the macroscopically identifiable tumour bed as: complete (0%, no residual tumour), subtotal (<10% residual tumour), partial (10 to 50% residual tumour) and minimal or no regression (> 50% residual tumour) as published by Becker K et al, Cancer 98:1521-30, 2003.
b) use the Mandard regression grading system for the primary cancers where an assessment is made comparing the amount of fibrosis to the amount of tumour (Figure 6).

CONFIDENTIAL
This material is the property of the Wales Cancer Trials Unit. Do not disclose or use except as authorised.

In addition, a separate statement whether there is evidence of tumour regression in lymph nodes (yes/no) should be provided. If there is evidence of regression in lymph nodes, the pathologist should record whether this regression is seen in peri-oesophageal, peri-junctional or peri-gastric lymph nodes (see Trial Pathology Case Report Form).

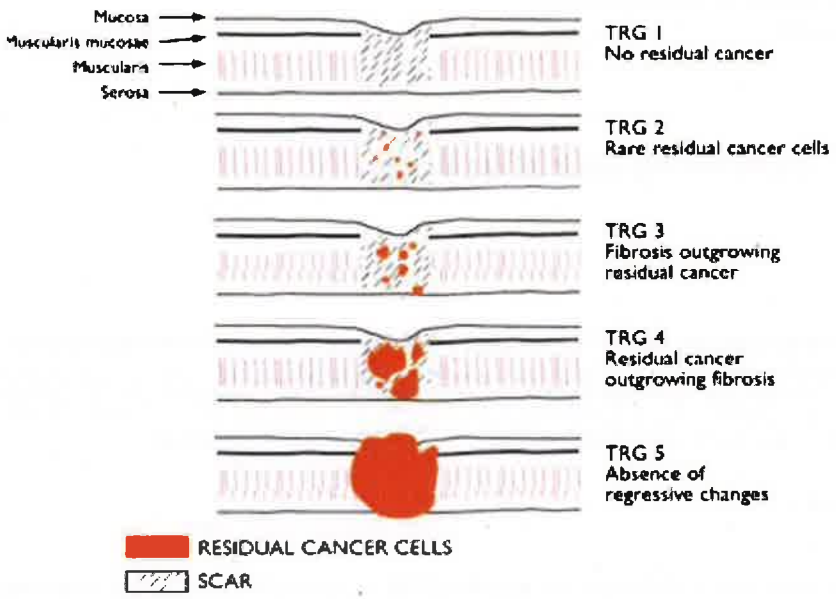

Figure 7. Tumour regression grading according to Mandard et al 1994.
Illustration from Gillham CM et al. Predicting the response of localised oesophageal cancer to neo-adjuvant chemoradiation World J Surg Oncol 5:97, 2007; doi:10.1186/1477-7819-5-97

5.10 Non-involved gastric mucosa

Although it is not necessary to report findings of the non-involved gastric mucosa as part of the NeoSCOPE Trial Pathology Form, it would be desirable to report the presence of glandular atrophy, intestinal metaplasia, dysplasia, background gastritis and Helicobacter pylori as part of the routine histopathology report.

6. **Central pathology review**

In order to ensure highest quality of pathology reporting of the whole trial, all H&E slides from the resection specimen, the diagnostic biopsy as well as the macroscopic photographs and the pathology report will be audited from all participants. There is no need for patient consent for this activity.

All H&E stained glass slides from both, the original diagnostic biopsy and the surgical resection specimens should be sent to Leeds together with the images and copies of the report. Patient identifiers (name and NHS number) should be blacked out before shipment. The pathology specimen number needs to remain visible on the slides and the copy of the reports. The pathologist or the local research nurse need to add 'NeoSCOPE trial' and the trial number onto the copy of the pathology report before shipment to Leeds.

The original H&E slides should be submitted for review, pathology departments are discouraged to produce recuts for the purpose of the central pathology review. In particular, the material in the pre-treatment block is considered as very precious for future translational studies and should not be cut unnecessarily. The slides will be returned as soon as possible after the review and after selected slides have been scanned.

As the rate of complete pathological response is primary endpoint of this trial, there will be particular emphasis on assessing the number of blocks taken from the tumour bed, number of nodes retrieved, location of retrieved nodes and histological review of the reported tumour regression in the primary cancer and the lymph nodes. During the central review process we aim to assess (a) agreement of the two regression grading systems (Mandard vs Becker), (b) agreement between local and central pathologist and (c) agreement of subjective assessment (regression grading) with quantitative morphometric measurements of tumour cell density performed in Leeds. Results of the central pathology review will be fed back to the local pathologist as appropriate.

CONFIDENTIAL
This material is the property of the Wales Cancer Trials Unit. Do not disclose or use except as authorised.

Please send slides, reports and pictures (CD-ROM) to:

Hayley SlaneyNeoSCOPE Trial, Department of Cellular Pathology
St James's Institute of Oncology, Leeds Teaching Hospitals NHS Trust
Bexley Wing, Level 5,Beckett Street
Leeds LS9 7TF

APPENDIX 5: Screening Investigations

Multi-slice CT scan

Patients should be staged with multi-slice CT to assess the local extent of the primary tumour, the presence of loco-regional lymphadenopathy and distant metastatic disease. A water load is used, dysphagia permitting, to maximise oesophageal and gastric distension. Intravenous contrast should be used and CT scan of the thorax and abdomen acquired at 1.25-2.5mm and reformatted at 5mm for viewing (RCR Guidelines 2006).

Endoscopic Ultrasound

Patients should be staged with endoscopic ultrasound (EUS) to assess the extent of visible and sub-mucosal tumour, infiltration by the primary tumour of surrounding mediastinal structures and the presence and site of site of significant lymhpadenopathy. The following will be recorded:

Location of disease and anatomical structures should be described according to the distance in cms from the incisor teeth.

OGD: Proximal and distal tumour extent (cms from the incisor teeth)
Amount of gastric involvement (measurement from oesophago–gastric junction identified as the proximal border of the gastric rugal folds)

EUS: Reference point e.g. top of arch of aorta, tracheal carina
Proximal and distal tumour extent (including submucosal disease)
T stage of tumour
If T4, record involvement of surrounding structures (e.g. right/left parietal pleura, aorta, trachea, diaphragmatic crus etc)
Pathological appearing lymphadenopathy, including distance and station (right/left tracheal, aortopulmonary window, subcarinal, paraoesophageal with relationship to tumour, paracardial, left gastric)

CONFIDENTIAL
This material is the property of the Wales Cancer Trials Unit. Do not disclose or use except as authorised.

APPENDIX 6: TNM classification for oesophageal cancer – comparison between the 6th and 7th edition

The current TNM classification criteria have been updated to the 7th edition of the UICC (Union for International Cancer Control) TNM staging system. We urge trial staff to use the 7th edition throughout the trial. A table summarizing the 6th and 7th edition is presented below for a quick and easy reference.

Staging	6th Edition	7th Edition
TX	Primary tumour cannot be assessed	Primary tumour cannot be assessed
T0	No evidence of primary tumour	No evidence of primary tumour
Tis	Carcinoma in situ	Carcinoma in situ /High-grade dysplasia
T1	Tumour invades lamina propria or submucosa	T1a: Tumour invades lamina propria or muscularis mucosae; T1b: invades submucosa
T2	Tumour invades muscularis propria	Tumour invades muscularis propria
T3	Tumour invades adventitia	Tumour invades adventitia
T4	Tumour invades adjacent structures	T4a: Tumour invades pleura, pericard, peritoneum or diaphragm; T4b: invades aorta, vertebral body or trachea
NX	Regional lymph nodes cannot be assessed	Regional lymph nodes cannot be assessed
N0	No regional lymph node metastasis	No regional lymph node metastasis
N1	Regional lymph node with viable tumour cells	1 to 2 regional lymph node with viable tumour cells
N2	N/A	3 to 6 regional lymph node with viable tumour cells
N3	N/A	> 6 regional lymph node with viable tumour cells
MX	Distant metastasis cannot be assessed	Distant metastasis cannot be assessed
MO	No distant metastasis	No distant metastasis
M1	Distant metastasis	Classified as 'distant metastsis' in addition to tumour in distant organs such as lung, liver, bone etc)

This material is the property of the Wales Cancer Trials Unit. Do not disclose or use except as authorised.

WALES CANCER TRIALS UNIT

Contact Details:

School of Medicine
Cardiff University
6th Floor
Neuadd Meirionnydd
Heath Park
Cardiff
CF14 4YS

General Telephone Number: 029 2068 7500
General Fax: 029 20687501

Email: neoscope@cardiff.ac.uk

Visit our website: www.wctu.org.uk

CONFIDENTIAL
This material is the property of the Wales Cancer Trials Unit. Do not disclose or use except as authorised.
