# Supplementary material for: NEOSCOPE: A randomised phase II study of induction chemotherapy followed by oxaliplatin/capecitabine or carboplatin/paclitaxel based pre-operative chemoradiation for resectable oesophageal adenocarcinoma
Source: Eur J Cancer. 2017 Mar;74:38–46. doi: 10.1016/j.ejca.2016.11.031 (PMC5341738; doi:10.1016/j.ejca.2016.11.031)
Supplement: Supplementary file 2 [file mmc2.pdf]

Radiotherapy Treatment Planning and Delivery

NeoSCOPE: Neo-adjuvant Study of Chemoradiotherapy in OesoPhagEal Cancer

A randomised Phase II study of two pre-operative chemoradiotherapy regimens (oxaliplatin and capecitabine followed by radiotherapy with either oxaliplatin and capecitabine or paclitaxel and carboplatin) for resectable oesophageal cancer.

Radiotherapy Guidance and Procedures Version 4.0
8th September 2014

Eudract No: 2012-000640-10
Funder: Cancer Research UK
Name of Sponsor: Velindre NHS Trust

Authorised by:

Name:	Tom Crosby	Role:	Chief Investigator
Signature:	______________________	Date:	08/09/2014
Name:	Chris Hurt	Role:	Scientific Lead, WCTU
Signature:	______________________	Date:	08/09/2014

Table of Contents

1 INTRODUCTION 4
1.1 Recommended Staging Investigation for Radiotherapy Planning 4
1.2 Dose prescription and fractionation 5
1.3 The management of unscheduled gaps in radiotherapy treatment 5

2 DEFINITION OF TREATMENT VOLUMES 6
2.1 Radiotherapy localisation 6
2.2 Treatment delivery 6
2.3 Target volume definition (TVD) 7

3 MIDDLE 1/3RD TUMOUR TARGET DEFINITION PROTOCOL 8

4 LOWER 1/3RD AND GOJ TUMOUR 3D CT TARGET DEFINITION PROTOCOL 10

5 LOWER 1/3RD AND GOJ TUMOUR 4D CT TARGET DEFINITION PROTOCOL 12

6 OUTLINING OF ORGANS AT RISK (OAR) 14

7 TREATMENT PLAN OPTIMISATION 15
7.1 Dose limitations of Volumes of Interest (VOI) and of Organs at Risk (OAR) 15

8 TREATMENT VERIFICATION 16

9 COMMUNICATION WITH THE SURGICAL TEAM. 17

10 RADIOTHERAPY QUALITY ASSURANCE 18
10.1 Pre-trial quality assurance 18
10.2 On-trial quality assurance 18

APPENDIX 1 *NODAL REGIONS AT RISK FOR LOWER OESOPHAGEAL, TYPE I AND TYPE II TUMOURS EXTENDING BELOW THE GASTROESOPHAGEAL JUNCTION* 20

APPENDIX 2 *LOWER OESOPHAGEAL CASE EXAMPLES* 22

APPENDIX 3 *MIDDLE 1/3 CASE EXAMPLES* 24

APPENDIX 4 *DELINEATION OF HEART VOLUME* 25

APPENDIX 5 *DELINEATION OF STOMACH VOLUME* 26

APPENDIX 6 GUIDANCE ON THE USE OF CONTRAST FOR 4D-CT 28

REFERENCES 29

1 Introduction

The protocol for NeoSCOPE has been developed through a UK Upper GI Radiotherapy Consensus Working Group.

The key points of reference for this work are the existing SCOPE 1 RT protocol; an Upper GI Radiotherapy Planning Workshop held October 27th 2011, Bristol, UK Patterns of Failure references (Button et al 2009 [1], Dresner et al 2001 [2] and the EORTC-ROG Guidelines for neo-adjuvant radiation of adenocarcinomas of the GE junction and stomach [3].

This document outlines the key radiotherapy principles that will be used within the NeoSCOPE trial.
Any centre wishing to deviate from this radiotherapy planning and delivery document should seek agreement from the Chief Investigator before any patient is recruited.

1.1 Recommended Staging Investigation for Radiotherapy Planning

All patients should have a CT scan thorax, abdomen (+/- pelvis). It is highly recommended that diagnostic scans are reviewed, ideally along with the planning scans, with specialist Upper GI radiologists.

Wherever possible, all patients should have an attempted EUS. In approximately 10% of cases, the EUS probe will not safely traverse the primary tumour. Whilst this varies according to user experience and the use of certain equipment (such as the use of the blind paediatric scope) valuable information can still be gained in respect to the proximal disease. However, it may be omitted in the rare situations where the tumour cannot be traversed safely, and the local MDT believe that it is not necessary on balance between benefit and risks. In this situation a CT/PET is mandatory.

EUS staging should be performed within the context of a specialised Upper GI MDT. References to anatomical landmarks visible on the CT Planning Scans are vital (e.g. the superior extent of the aortic arch or the carina) together with clear recording of the extent of disease, including submucosal and nodal disease.

CT/PET has an established role throughout the UK in terms of staging oesophageal cancer. It is very likely that it will also have a valuable role in radiotherapy planning but there are a number of issues that require further evaluation such as:

- the incorporation of CT/PET for RT planning or the use of fusion software
- the appropriate method of segmentation for use for RT outlining.

The availability of PET images may be of assistance in determining the extent of disease, particularly radially, but the volume as defined by CT and EUS should not be reduced based on PET findings alone.

In the example below PET-CT was helpful in defining the lateral extent of disease because the soft tissue resolution is limited in the posterior mediastinum on CT.

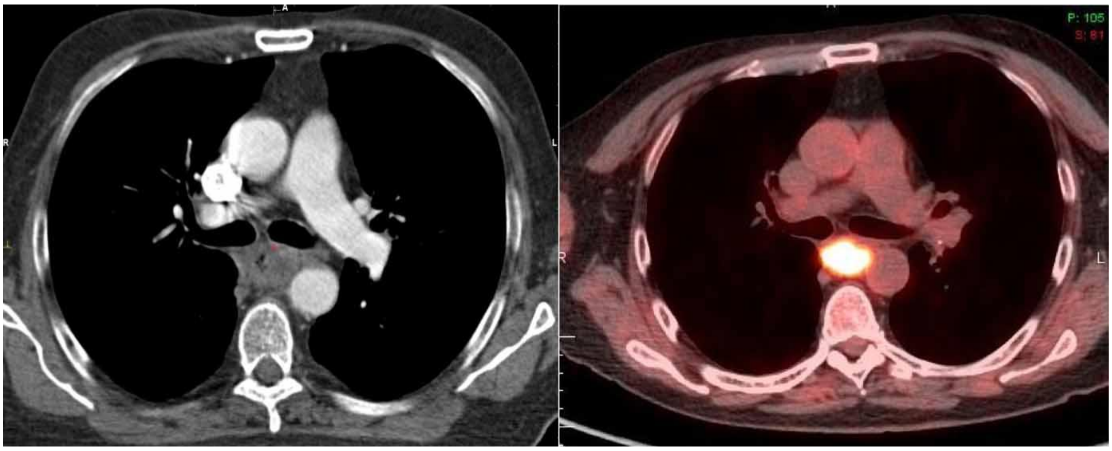

1.2 Dose prescription and fractionation

For NeoSCOPE the total dose of radiation will be 45Gy in 25 fractions treating once daily, 5 days per week.

The dose will be prescribed and recorded as per ICRU 50/62.

1.3 The management of unscheduled gaps in radiotherapy treatment

In the event of unscheduled gaps to radiotherapy treatment, these should be managed in line with the latest RCR Guidance (The Timely Delivery of Radical Radiotherapy: Standards and Guidelines for the Management of Unscheduled Treatment Interruptions, Third Edition, 2008 - full document available at www.rcr.ac.uk). In the case of cancers of the oesophagus, adenocarcinoma should be managed as Category 1 patients.

2 Definition of treatment volumes

2.1 Radiotherapy localisation

In summary:

A planning CT scan should be performed within 2 weeks of starting induction chemotherapy.

Slice thickness should be no greater than 3mm.

Intravenous contrast should be used (providing adequate renal function), but oral contrast should not.

Patients should be asked to fast for 2 hours and then drink 200mls of liquid 30 minutes prior to CT planning *and* treatment in an attempt to reproduce the same anatomical position of the stomach due to filling throughout treatment. Patients who have an NG tube inserted and are unable to drink due to dysphagia, should use their tube for this purpose.

Patients should be scanned in the treatment position, namely supine with arms above head with knee support and immobilisation with thermoplastic device or vacuum cushion as per local protocols.

The extent of the scan would be from 1cm superior to the apices of the lungs to the bottom of the L4 vertebra in order to ensure inclusion of all OARs (lungs, liver, kidneys and stomach). It is particularly important that the full lung volume is scanned as radiotherapy dose received by lung tissue and the volume of lung spared will be reported in both absolute and relative terms as part of this study. Diagnostic information should be taken from the diagnostic CT scan, EUS (referenced to CT identifiable structure) and CT/PET.

Fused CT/PET images should not be used within the NeoSCOPE study until further evaluation is undertaken and without discussion with the Chief investigator.

For lower 1/3 and GOJ tumours 4D planning scans should be considered, **and where performed should follow the protocol below**. Other forms of motion management such as respiratory gating and tracking must not be used without prior discussion with the Chief Investigator.

Tumours of the middle 1/3 do not require 4D CT.

IMRT should not be used for NeoSCOPE until further evaluation in this pre-operative setting is undertaken.

2.2 Treatment delivery

A single phase 3D-conformal treatment plan should be produced and delivered from 4-5 gantry angles. There should be no cone down or boost volumes but more than one beam may be delivered from the same gantry angle, e.g., filler fields to cool hotspots or opposed superior-inferior wedges to improve coverage at the extents of the PTV.

If in doubt please contact the RTQA team via Gareth Jones (Gareth.Jones9@wales.nhs.uk) to discuss any queries.

2.3 Target volume definition (TVD)

Eligibility for the NeoSCOPE trial includes patients with oesophageal and gastro-oesophageal junctional (GOJ) carcinomas, staged at least T3 and/or N1, who are to undergo an oesophagectomy.

As such, and for the benefits of this protocol, based on EUS patients can be divided into:
middle 1/3rd - defined here as primary tumour centre between 24cm and 32cm ab oral.
lower 1/3rd and GO junction - defined here as primary tumour centre from 32cm ab oral to less than 3cm distal to the anatomical GOJ*.

*Note eligibility criteria excludes patients with primary tumours with distal extension of more than 3cm below the GOJ.

Where the passage of the EUS scope across the tumour has not been possible, findings from diagnostic endoscopy and PET imaging must be used to define the disease centre.

Target volumes are defined following the principles of ICRU 50 and 62. Where there is overlap between the PTV and spinal cord PRV, or the two structures are in close proximity, it is up to the local Principal Investigator, where necessary on discussion with the Chief Investigator, to weigh up the risk-benefits of compromising the target volume and risk of toxicity to the spinal cord.

Wherever possible please conform to the suggested nomenclature used in this protocol (including upper/lowercase characters & spaces or additional characters).

Dialogue with a specialist Upper GI radiologist regarding target volume definition is encouraged. For the purpose of this protocol TVD is defined according to anatomical subsite (middle or lower 1/3rd/GOJ) and, for lower 1/3rd/GOJ tumours only, according to the availability of 4D CT.

3 Middle 1/3rd tumour Target Definition Protocol

Gross tumour volume (GTV)

The GTV consists of the gross tumour, any involved nodes and the circumference of the oesophagus at the level of disease. In situations where nodal involvement is found superior or inferior to the primary tumour, the radial margin of the oesophagus between these structures forms the radial margin of the GTV. At the level of the involved LNs, the circumference of the oesophagus is also included.

The GTV should encompass disease considered positive in conjunction with Upper GI radiologist on consideration of all the diagnostic modalities i.e. should not be reduced in case of negative CT/PET if considered positive on other modalities. It is not possible to state in this protocol the interpretation which might be made of CT, EUS and CT/PET in any particular situation, which as well as the sensitivity and specificity of these investigations will depend on the certainty of the specialist radiologist. For example a slightly enlarged lymph node on CT may appear benign on EUS and be non-avid with respect to a strongly avid primary tumour on CT/PET and may not be considered malignant. Notwithstanding the above however, a node considered malignant on EUS should always be included.

Clinical target volume (CTV)

Cranio-caudal
The GTV is copied and labelled 'CTVA'. This is grown manually along the axis of the oesophagus and should be both 20mm superior to the proximal GTV margin and 20mm inferior to the distal GTV margin if defined by primary tumour. However, if the superior / inferior extent of disease is defined by nodal disease that is at least 10mm away from the primary tumour, CTVA only needs to be extended by 10mm beyond nodal disease, again along the axis of the oesophagus. The whole circumference of the oesophageal wall should be included throughout the length of CTVA.

Circumferential
CTVA is then copied and labelled 'CTVB'. It is grown by adding 10mm in right-left and anterior-posterior directions using the Treatment Planning System (TPS). CTVB is then edited to exclude lung, pericardium, large vessels, trachea and right/left main bronchi and the vertebrae.

Editing the CTVB (yellow) avoids unnecessary inclusion of the lung and heart. The example below highlights the difference between editing CTVB rather than using a 10mm geometric axial margin (blue).

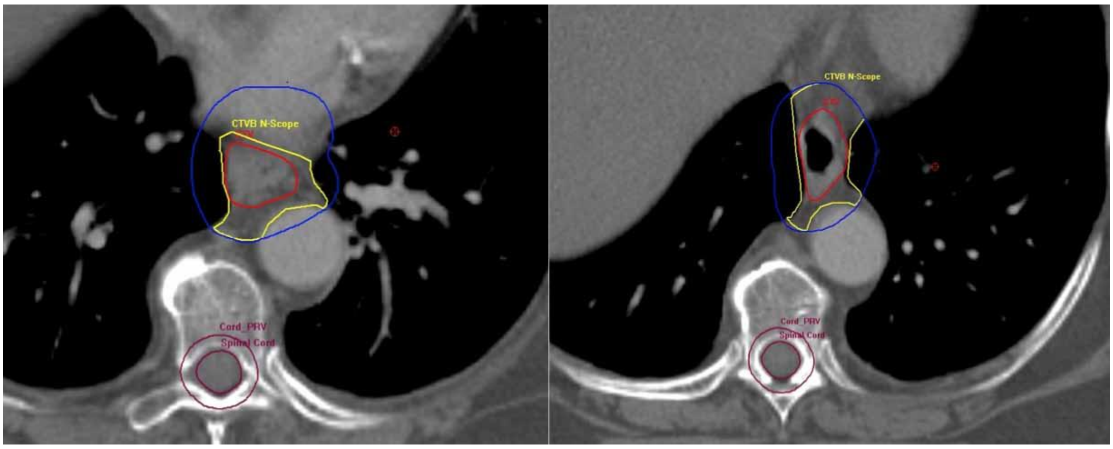

Planning target volume (PTV)

CTVB is copied and automatically grown using the TPS by 10mm superiorly (5mm IM and 5mm SM), 10 mm inferiorly (5mm IM + 5mm SM) and 10mm (5mm IM and 5mm SM) circumferentially. This new volume is labelled PTV.

* The posterior margin may be reduced where CTVB is directly abutting vertebrae (IM not required as tumour cannot move into vertebrae, only SM is required), to a minimum distance of 5mm. (This may be done using PTV 5mm and PTV 10mm and the optimum margin chosen on a slice by slice basis. This final PTV should be labelled PTV).

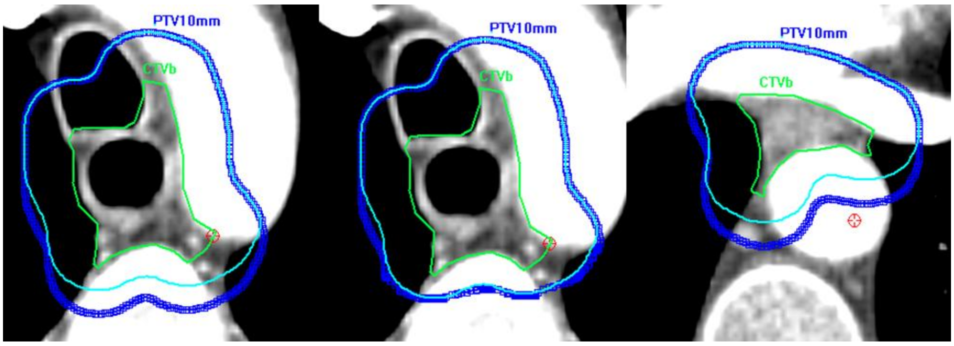

In these images, CTVB is shown in dark green, PTV10mm in dark blue, and PTV5mm in turquoise.
In the left image, the posterior border of PTV10mm is manually reduced to PTV5mm (middle image) because CTVB is directly against the vertebral body. The right hand image from the same patient more inferior shows that CTVB is more anterior and no longer against the vertebrae, and therefore the posterior border remains at PTV10mm.

In summary:

CTV = GTV + occult disease (20mm S-I + 10mm circumferential) [1]

[1] edited to exclude OARs

PTV = CTV + IM + SM
= CTV + 10mm circumferential[1], 10 mm S-I

[1] except posteriorly where PTV margin is reduced where CTV + IM crosses the vertebra.

4 Lower 1/3rd and GOJ tumour 3D CT Target Definition Protocol

It is clear that with respect to lower 1/3rd/GOJ tumours there is significant movement, particularly due to respiration. Where possible centres are encouraged to follow the 4D CT planning protocol which will be evaluated within the NeoSCOPE trial, but if not available centres should follow the 3D protocol, both of which are described in this document. ***Any centre wishing to undertake 4D scanning within the NeoSCOPE trial should first seek permission from the trial manager.***

Gross tumour volume (GTV)

Above the GOJ GTV consists of the gross tumour, any involved nodes and the circumference of the oesophagus at the level of disease. In situations where nodal involvement is found superior or inferior to the primary tumour the radial margin of the oesophagus between these structures forms the radial margin of the GTV. At the level of the involved LNs the circumference of the oesophagus is also included.

Below the GOJ involved nodes are no longer in continuity with the oesophagus, and so are outlined separately and labelled GTVN. Where more than one GTVN is required these may be helpfully named separately e.g. GTVN1, GTVN2 etc, depending on the TPS available.

The GTV should encompass disease considered positive (in conjunction with upper GI radiologist) on any of the diagnostic modalities; i.e. it should not be reduced in case of negative CT/PET if seen on other modalities. It is not possible to state in this protocol the interpretation made of CT, EUS and CT/PET which as well as the sensitivity and specificity of these investigations will depend on the certainty of the specialist radiologist. For example a slightly enlarged lymph node on CT may appear benign on EUS and be non-avid with respect to a strongly avid primary tumour on CT/PET and may not be considered malignant. Whereas a node considered malignant on EUS should always be included.

Clinical target volume (CTV)

The CTV is made up of the GTV including a margin for occult disease below the GOJ as described below:

Cranio-caudal
The GTV is copied and labelled ‘CTVA’. Superiorly this is grown manually along the axis of the oesophagus and should be 20mm where the proximal margin is defined by primary tumour. However, if the superior extent of disease is defined by nodal disease that is at least 10mm away from the primary tumour, CTVA only needs to be extended by 10mm beyond nodal disease, again along the axis of the oesophagus. The whole circumference of the oesophageal wall should be included.

Inferiorly CTVA is grown manually along the axis of the oesophagus for 20mm, or until the GOJ is reached (if less than 20mm).

Below the GOJ, any GTVN are grown by a 5mm margin in all directions and labelled ‘CTVN5mm’. This margin defines a minimum margin around positive nodes to assist in the delineation of CTVB.

Circumferential
CTVA is copied and labelled ‘CTVB’. It is grown by adding 10mm in right-left and anterior-posterior directions using the Treatment Planning System (TPS).

Above the diaphragm CTVB is edited to exclude lung, pericardium, large vessels and the vertebrae.

Below the GOJ CTVB is grown manually to 20mm below GTV. This volume includes CTVN5mm, and the elective nodal regions at high risk of microscopic spread along the lesser curve of stomach, the left gastric artery and coeliac region <u>where these lie within 20mm of the GTV.</u>

Superiorly, this volume includes the fat space below the diaphragm around the cardia and gastro-hepatic fat between the lesser curve and the medial liver edge. Inferiorly, it includes the fat space around the coeliac artery*, bounded by the pancreas anteriorly, small bowel and liver laterally, and aorta posteriorly (see appendix for examples), (*where this is within 20mm of the GTV. I.e. this is not full elective lymph node irradiation. Rather, this is growth of the CTV in the direction of known lymphatic spread (see lymph node stations below))

An assessment of the potential for mucosal spread into the stomach laterally and posteriorly must be made on a case by case basis. The extent of this volume is hard to define anatomically but is usually no greater than 2cm in lateral extent. In order to include LNs *along* the lesser curve, it is anticipated that some of the lesser curve will be included (see Appendix 2 for examples) especially in tumours of the GOJ and/or with gastric extension.

The elective nodal regions of the splenic hilum, greater curvature, and short gastric vessels are **not** included, even for patients with Type II GOJ tumours.

Planning target volume (PTV)

CTVB is copied and automatically grown using the TPS by 10mm superiorly * (5mm IM and 5mm SM), 15mm inferiorly (10mm IM + 5mm SM) and 10mm (5mm IM and 5mm SM) circumferentially. This new volume is labelled PTV.

* The posterior margin may be reduced as for Mid 1/3 cases, where CTVB is directly abutting vertebrae. Because there is no need to include a margin for IM into the Vertebrae, on the axial slices where CTVB is against the vertebrae, PTV can be reduced to a minimum margin of 5mm (see section on middle 1/3 tumours for further guidance on how this can be achieved).

In Summary:

CTV = GTV + occult disease (20mm S-I + 10mm circumferential) [2]

[2] edited to exclude OARs and includes 'volume at risk' below GOJ and never compromise CTVN5mm

PTV = CTV + IM + SM
= CTV + 10mm sup + 15mm inf, 10mm circumferential [3]
[3] reduced posteriorly where CTV + IM extends across vertebrae

5 Lower 1/3rd and GOJ tumour 4D CT Target Definition Protocol

4DCT Outlining Principles

4DCT allows the tumour motion to be imaged over a patient's respiratory cycle, thereby allowing patient specific volumes to be generated which account for physiological organ motion. This guidance document provides detail on the process of generating an Internal Target Volume (ITV) utilising 4DCT data sets generated from the corresponding phases of respiration. In order to encompass all the effects of motion on the target volume on, the ITV can only be made bigger following the review of all the datasets from the corresponding phases of respiration.

It is recommended that patients received both a 3DCT and 4DCT scan in a single simulation session. The inclusion of the 3DCT scan ensures that data is available for planning should the 4DCT data be deemed wholly or partially unusable (due to machine limitations e.g. scan length limitations, or patient suitability e.g. imaging artefacts due to the patient's breathing pattern). Ideally both scans should be acquired with IV contrast enhancement. If this is not possible, then the 3DCT scan should be acquired with contrast and used as the planning reference data set and the 4DCT data set used solely to derive the ITV. In this case the superior border of the 4DCT scan can be lowered to the level of the superior aortic arch or carina as the total lung volume will be available on the 3DCT reference scan. In addition, any patient movement that has occurred between the acquisition of 3D and 4D data sets should be accounted for.

A minimum of 10 4DCT reconstructed phases distributed throughout the patient's respiratory cycle are recommended.

Examples of IV contrast timings for 4DCT are presented in appendix 6.

In instances where the 4D data sets can be acquired with IV contrast enhancement and include all the organs at risk, then it is acceptable to use the time weighted average data set for planning and dose calculations. A 3D scan is not required in this instance.

It is acknowledged that different centres utilise different methods to generate an ITV volume, through either hardware capability, software functionality or a combination of the two. Two examples of methods used to derive the ITV are given below. Centres wishing to use other methods should contact the RTQA team to check suitability for inclusion in NeoSCOPE.

<u>**4-D Outlining Method 1** (based on the Leeds- Cardiff experience).</u>

Gross tumour volume (GTV) and Clinical target volume (CTV)

From the 4DCT data sets, identify the extreme phases of motion (MaxIn and MaxEx) and the phase that best represents the time-weighted average (Mid). GTV, CTVA and CTVB contours are generated (as per the lower 1/3rd 3D protocol) using the three phases as defined above, giving three GTV volumes (GTVMaxIn, GTVMid, GTVMaxEx), three CTVA volumes (CTVAMaxIn, CTVAMid, CTVAMaxEx) and three CTVB volumes (CTVBMaxIn, CTVBMid and CTVBMaxEx). The ITV is defined as the composite CTVB volumes, edited to account for any additional motion (i.e. the editing will result in the ITV only being made bigger, to account for the maximum extent of these motion effects) seen from all other 4DCT phases.

The above contours must be associated with the planning CT data set. Where a 4DCT scan contains all the required OARs, the phase that best represents the time weighted average) should be used as the planning (reference) data set. If the 4DCT scan does not contain all the required OARs then the 3DCT scan must be used as the planning (reference) data set.

Set up margin (SM)
This margin is applied for treatment inaccuracies, such as set up error, and for the purpose of this protocol ITV is grown by 5mm in all directions using the TPS.

In Summary:

PTV = ITV +SM
= ITV + 5mm

Following the generation of the PTV, the organs at risk structures and planning should be carried out on the ref data set only.

4-D Outlining Method 2

Contour the GTV on the 3D contrast enhanced CT scan and label it GTV3D.
The 4D-CT scan is reconstructed in 10 respiratory phases. The inhale/exhale phases are identified (usually 0% and 50% respectively). Review all reconstructions to make sure the phases represent the extremes as sometimes 40% or 60% can represent exhale.

Contour the GTV in the extreme phases. (GTVMaxIn, GTVMaxEx). Using Boolean operators combine GTV3D, GTVMaxIn, GTVMaxEx to obtain GTVmotion. Review this volume 3 dimensionally on all phases of respiration and manually edit areas that not covered. Please note any respiratory phases requiring manual edits.
On the 3D contrast enhanced CT create the CTVA3D and CTVB3D from the GTVmotion (as per the lower 1/3rd 3D protocol). In order to obtain the ITV; make a copy of CTVB3D and name it CTVBMaxIn then proceed to edit the maximum inhale scan. Copy structure CTVB3D and name it CTVBMaxEx then proceed to edit on the maximum exhale scan.

Combine (Boolean operator) CTVBMaxIn and CTVBMaxEx to obtain ITV. Check that this ITV volume covers any unusual motion patterns in the respiratory phases noted during the creation of GTVMotion.

Set up margin (SM)
This margin is applied for treatment inaccuracies, such as set up error, and for the purpose of this protocol ITV is grown by 5mm in all directions using the TPS.

In Summary:

PTV = ITV +SM
= ITV + 5mm

Following the generation of the PTV, the organs at risk structures and planning should be carried out on the CECT image set
All patients will have verification with Cone beam CT as described in section 8

6 Outlining of Organs at risk (OAR)

Spinal Cord
The spinal cord should be outlined on slices which include or are within 20mm of the PTV in the superior and inferior directions.

Spinal Cord PRV
A Planning Risk Volume (PRV) for the cord is created to account for positioning error; the size of the margin added to the cord being commensurate with the accuracy of treatment delivery expected and, as such, the tolerance level allowed in portal image verification on treatment. For example, at Velindre Hospital an isotropic margin of 5mm generated around the spinal cord to create the Cord PRV necessitates no more than 5mm movement of the isocentre on treatment before corrective action is taken.

Lungs
The full extent of the right and left lungs are to be outlined, this should be done in such a way that the planning system will be able to calculate a combined lung Dose Volume Histogram (DVH).

Heart
The whole heart should be outlined to the extent of the pericardial sac (if visible). The major blood vessels (superior to the organ) and the inferior vena cava (towards the inferior extent of the heart) are excluded. Appendix 1 contains an example.

Liver
The whole liver should be outlined if its superior level is coincident with, or overlaps with, the inferior level of the PTV.

Kidneys
Both kidneys should be outlined separately if the superior level of either kidney is coincident with, or overlaps with, the level the inferior level of the PTV.

Stomach
The whole stomach should be outlined in such a way that a stomach DVH can be produced. This will be for evaluation only as a Region of Interest (RoI) and will not be an OAR with dose constraints.

7 Treatment Plan Optimisation

A single phase 3D conformal treatment plan should be produced.
The use of a 'type B' calculation algorithm is strongly recommended for dose calculation and optimisation.
IMRT should not be used for NeoSCOPE until further evaluation in this clinical setting is undertaken. In individual circumstances, if recommended locally for dosimetric considerations, this should be discussed with the Chief Investigator and QA team prior to treatment.

Beams should be delivered from no more than 5 gantry angles without prior discussion with the Chief Investigator If in doubt please contact the RTQA team via Gareth Jones (Gareth.Jones9@wales.nhs.uk) to discuss any queries.

Dose limitations of Volumes of Interest (VOI) and of Organs at Risk (OAR)

**Region of Interest / Organ at Risk**	**Dose Objective**	**Comments/ Secondary Considerations**
PTV if 'type B' algorithm used	V95% ≥ 99% – (0.4*%lung/PTV overlap)	V95% objective is individually determined based on the percentage of PTV which overlaps with lung tissue according to the formula adopted from Wills et. al. [4]
PTV if 'type A' algorithm used	V95% > 99%<br>and<br>D99% > 95%	
ICRU Maximum dose	D1.8cc < 107%	Defined as the maximum dose to 1.8cc of any structure within the external contour of the patient.
Spinal Cord PRV	D1cm3 < 40Gy	If the PTV lies close to or overlaps with the Spinal Cord PRV, the treating clinician may discretionally allow a point maximum dose up to 45Gy. Alternatively, they may report a PTV compromise (for type b algorithms this compromise must be noted regardless of the plan achieving the individualised PTV objective).
Combined Lungs	V20Gy < 25%	The aim should be to minimise dose to the lung wherever possible to V20Gy < 20%<br>Total lung volume and V5 lung, V5s lung and mean lung dose will be collected and reported on PAF but will not be a volume/dose constraint
Heart	V40Gy < 30%<br><br>V25Gy < 50%	Optimal objective – to be achieved where possible but at lower priority than other objectives.
Liver	V30Gy < 60%	
Individual Kidneys	V20Gy < 25%	
Stomach		This will not be defined as an organ at risk but data collected to explore correlation with toxicity.

8 Treatment Verification

It is recommended that the best available positional verification methods should be used to ensure correct delivery. <u>The use of cone beam CT matched to planning CT scans is mandated within this study.</u>

A cone beam CT is acquired prior to treatment and the entire PTV should be encompassed in the cone beam CT image. A patient specific volume ('clipbox') should be defined around the PTV by creating a region of interest that encompasses the PTV contour as transferred from the planning CT with a 1-1.5 cm margin in all directions. This includes partial volumes from normal structures in the vicinity: lungs, heart, vertebral bodies.

An automatic match process using local correlation algorithms (such as 'grey value') should be applied. Visually verify the resultant match, to ensure that the target is adequately encompassed within the PTV structure, and then manually repositioned if necessary.

The minimum protocol for on-treatment verification is for imaging the initial three fractions with on line correction, so that a correction for systematic error can be applied. Further imaging should continue with weekly imaging, or more frequently as per local department protocol if further inconsistencies are found. The isocentre should be moved if disagreement is seen in excess of agreed tolerance levels based on local study – typically 5mm. This process also allows radiographers to evaluate the whole set-up and thus to assess and correct systematic errors.

9 Communication with the Surgical Team.

In order to plan the patients operation, the surgeon will require a description of the PTV. In particular, the superior and inferior extent of this volume will need to be taken into account when considering the site of the surgical anastomosis.
To facilitate communication, the surgeons must be provided with a digital image, which indicates the position of the PTV in reference to anatomical landmarks, such as the carina.
The simplest way to achieve this, is to take a print screen snapshot of the DRR, or the CT-reconstructed carinal image, from the TPS.
To take a print screen image of the PTV:

With the TPS as the active window – press alt + PrtScn.

Load Microsoft Paint (under 'accessories' on the Windows programs menu bar)

In Paint, press Ctrl + v. (This will copy the image into Microsoft paint)

The image can then be cropped / moved – and if necessary can be anonymised

Then save the image and forward to the Surgeon.

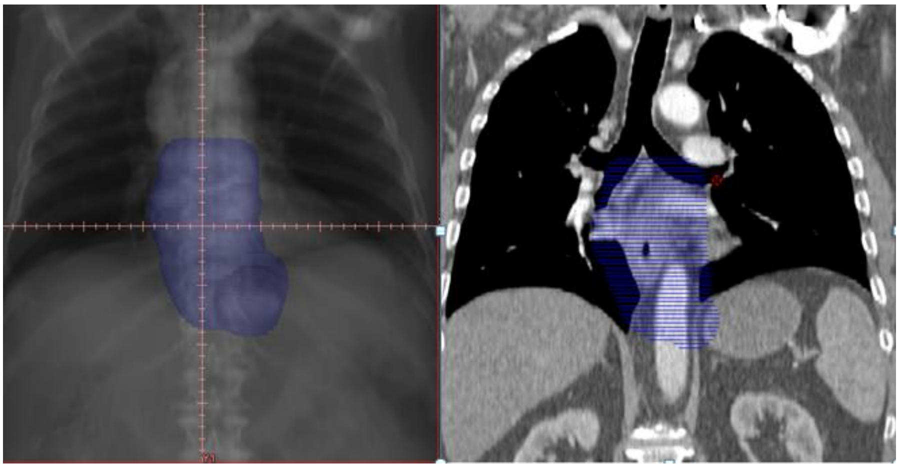

In these examples, the left image shows the PTV on the DRR, and right image shows the PTV on the CT coronal reconstruction from the TPS.

10 Radiotherapy quality assurance

We have taken the advice from the NCRI RTTQA Group that has recommended the procedure listed below. The necessary forms and details about how to upload plan data, questionnaires and planning documents can be obtained via the NeoSCOPE link at the NCRI Radiotherapy Clinical Trials Quality Assurance Group website: http://www.rttrialsqa.org.uk/.

10.1 Pre-trial quality assurance

Completion of an outlining exercise:
All centres who wish to participate in NeoSCOPE must satisfactorily complete a pre-accrual outlining benchmark case of a mid and lower oesophageal cancer case. Data should be uploaded using the RTTQA website. Outlines will be compared against a consensus reference volume (gold standard) derived from the outlines of TMG members.

Criteria for satisfactory completion will be at the discretion of the RTQA members of the TMG. Attention will be paid to correct interpretation of imaging (GTV) and ability to follow the protocol to create CTV and PTV. Written feedback will be provided to all centres.

For 4D centres attendance at either a workshop or completion of a 4D pre-trial test case will be mandated as part of the pre-trial QA of the 4D outlining. A 4D test case will need be completed in addition to a 3D case.

Completion of a planning exercise:
One pre outlined patient per centre should be planned and the data uploaded following the instructions on the RTTQA website. A Plan Assessment form (PAF) should be completed and submitted at the same time.

Production of a Radiotherapy Process Document – in line with which all trial patients will be scanned, planned and treated.

Completion of the following questionnaires:
- National radiotherapy trials QA baseline questionnaire
- National radiotherapy trials QA staff questionnaire

10.2 On-trial quality assurance

Full planning data for each patient (planning CT, structures, plan and dose) should be uploaded following the instructions on the website above. A PAF should be completed and submitted at the same time.

Real time review
There will be real-time review of the outline and plan for the first patient case from each centre and all cases submitted up until the first NeoSCOPE toxicity analysis assessment. A second case will also be reviewed should there have been an issue with the first case. This outlining and planning assessment will be in ‘real time’ and undertaken by the QA NeoSCOPE subgroup. As such, real time review will require timely uploading of the data from the centres. We request that CT data and plan data (structures, plan, dose) in addition to plan assessment forms (PAFs) be submitted via a secure NHS server. Copies of the PAF should be accessed from the RTTQA website. Please also submit the reports for the CT, EUS and PET scan. The QA NeoSCOPE subgroup will process the review within 3 working days of receipt. Early submission of the outlining data is strongly encouraged to allow adequate time for review prior to the start of radiotherapy planning. It is left to the centre’s

discretion as to whether they wish to start the planning process with the pre-approval outlines while awaiting this feedback.

Timely retrospective review

Cases that no longer require ‘real time review’ will be subject to ‘timely retrospective’ review. Outlines and plans should be submitted for review by the RTQA team as soon as possible and the RTQA team will give feedback to the centre within 2 weeks of the start of treatment. Only deviations from protocol considered a significant risk to the patient/trial outcome will require a change in treatment plan at this stage.

Appendix 1 *Nodal regions at risk for lower oesophageal, type I and type II tumours extending below the gastroesophageal junction*

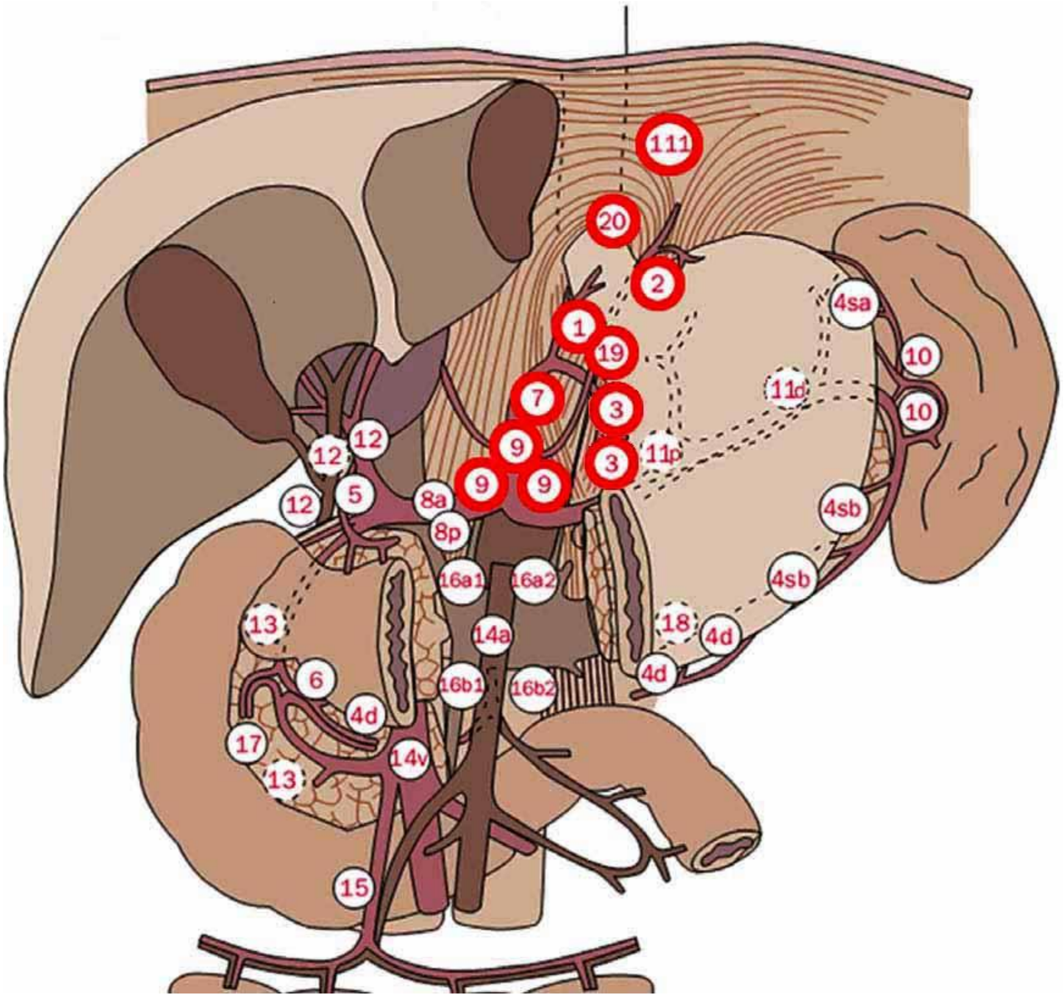

Elective nodal regions which are considered at risk for type I and type II tumours in NeoSCOPE are those that lie 2cm below the GTV in the regions identified in red above. Using the 2nd English edition of the Japanese classification of gastric carcinoma these are:

1+2	Left and right paracardial.
7	Lymph nodes along the left gastric artery.
3	Lymph nodes along the lesser curvature.
9	Lymph nodes around the celiac artery.

It does not include nodes along the short gastric vessels, distal splenic artery or around the splenic hilum.

Nodal regions at risk: Right and left paracardial nodes.

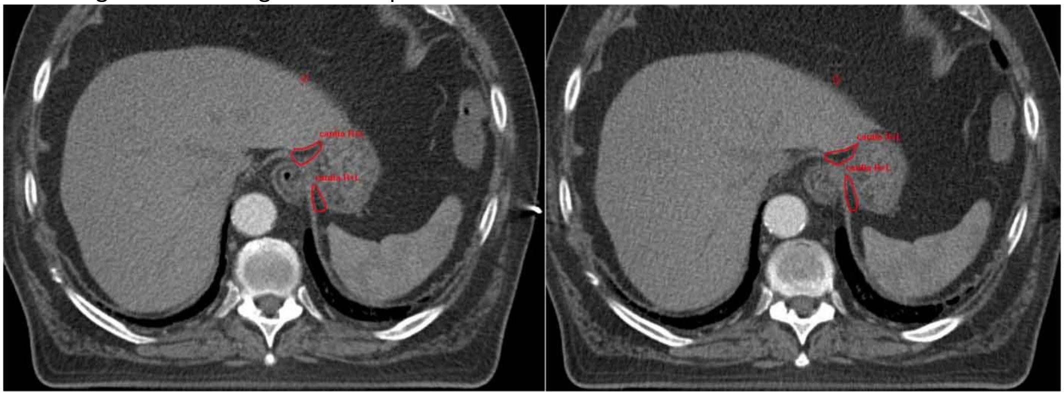

Nodal regions at risk: The left gastric artery and lesser curvature.

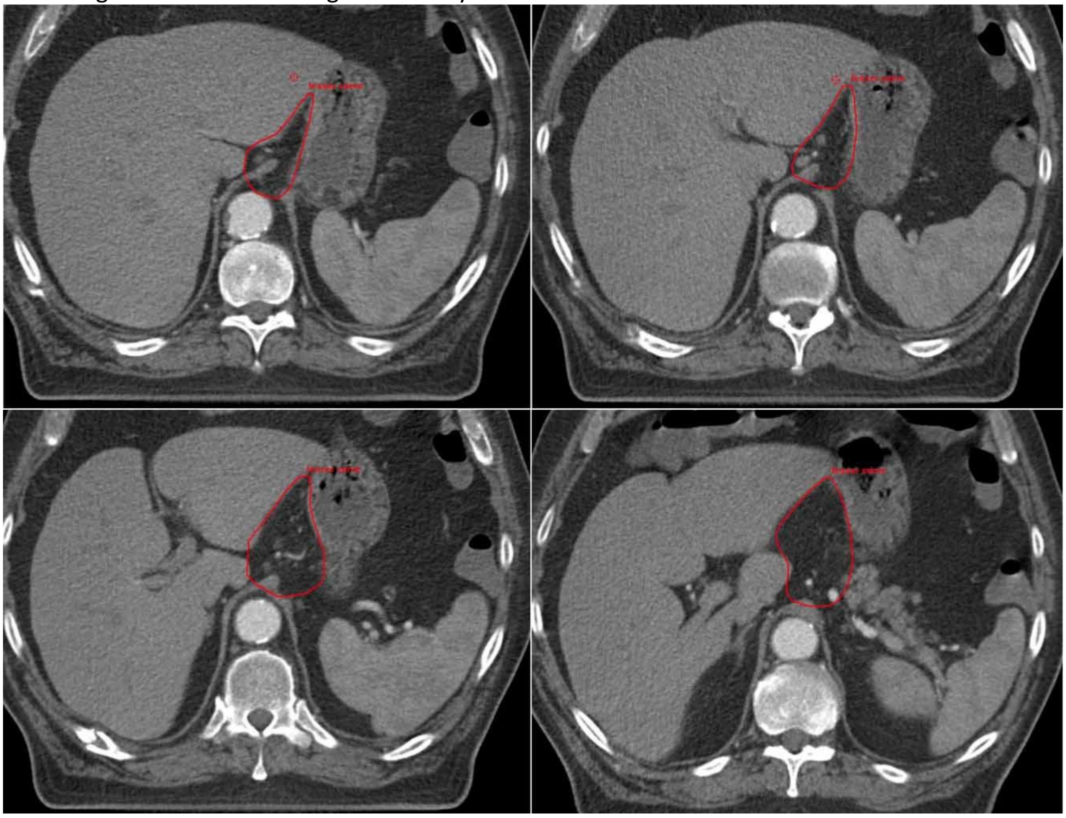

Nodal regions at risk: Coeliac Nodes.

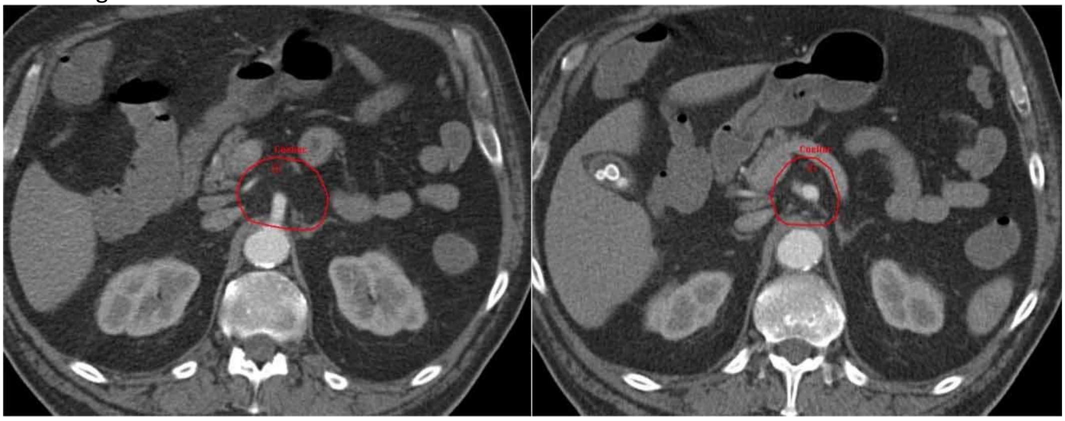

Appendix 2 *Lower oesophageal case examples*

Case 1: 55 year old female with type I, poorly differentiated adenocarcinoma of the GOJ which extends from 30-36cm on EUS, just above a small hiatus hernia from 36-38cm on EUS. The carina was at 24cm and left crus of the diaphragm at 38cm. No nodes were identified, or distant disease on PET CT scanning. Final clinical stage T3N0M0.

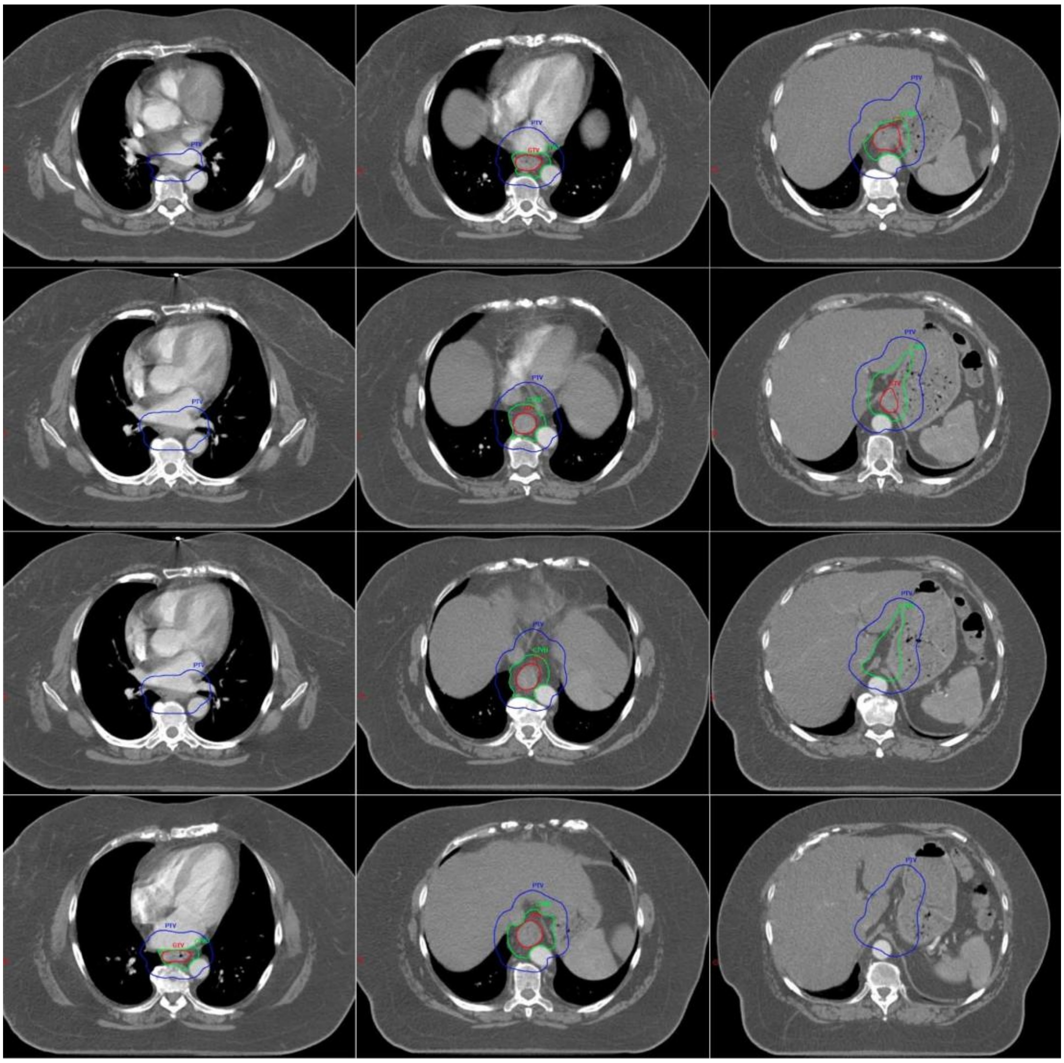

Case 2: 65 year old male with a 6cm moderately differentiated adenocarcinoma of the lower oesophagus and GOJ on PET and CT scanning, with no evidence of nodal disease. An EUS was attempted but they were unable to pass the scope beyond the stricture; the carina was at 26cm, and the superior aspect of the tumour at 36cm.

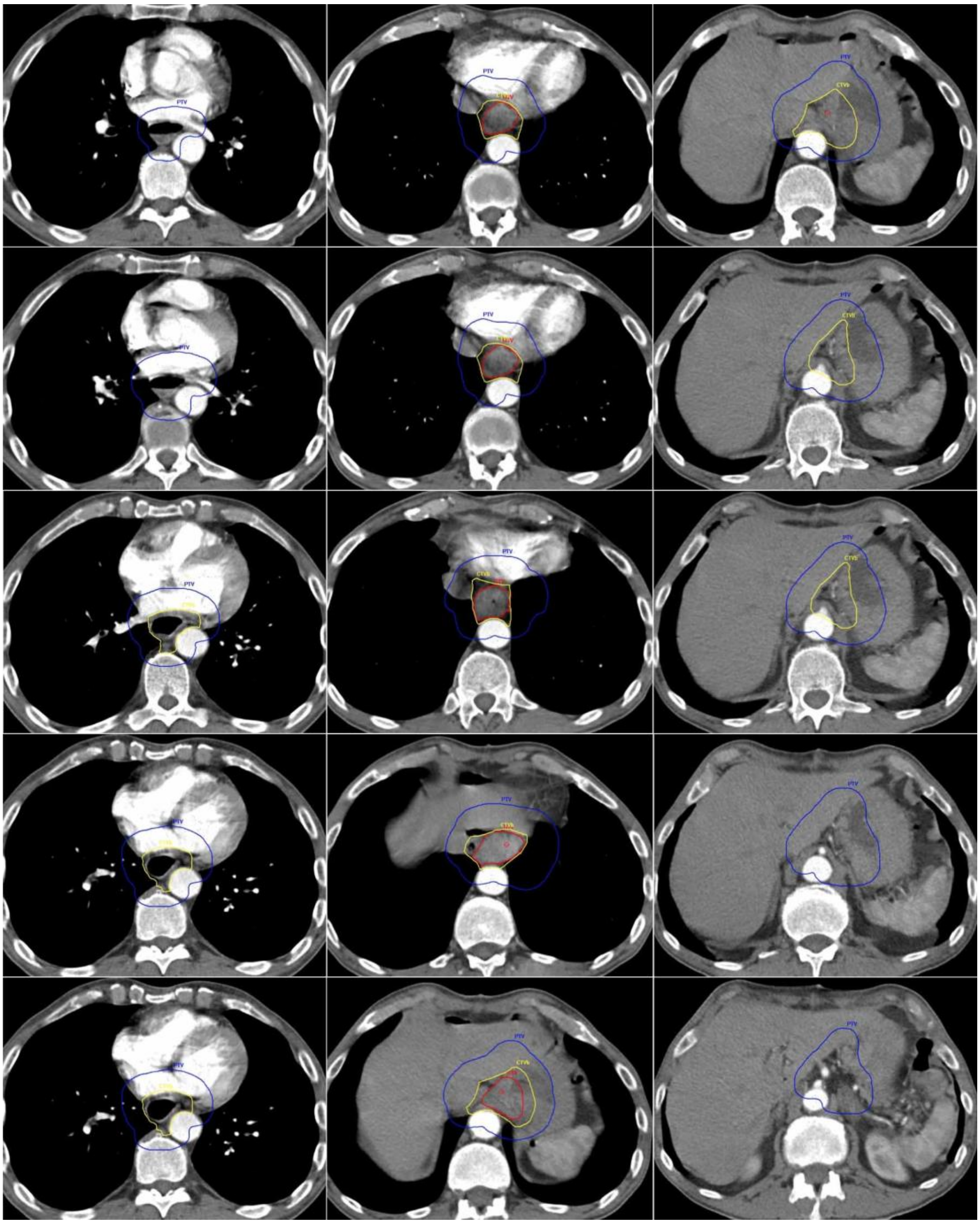

Appendix 3 *Middle 1/3 case examples*

68 year old female, with a 5 x 2cm polypoidal mass in the mid oesophagus on EUS from 30-35 cm, with submucosal spread from 28-30cm, starting at the level of the carina. Anteriorly in the peritumoural region there is a 6mm suspicious node; this is not seen on the diagnostic CT scan or PET scan. Clinical stage T3N1M0.

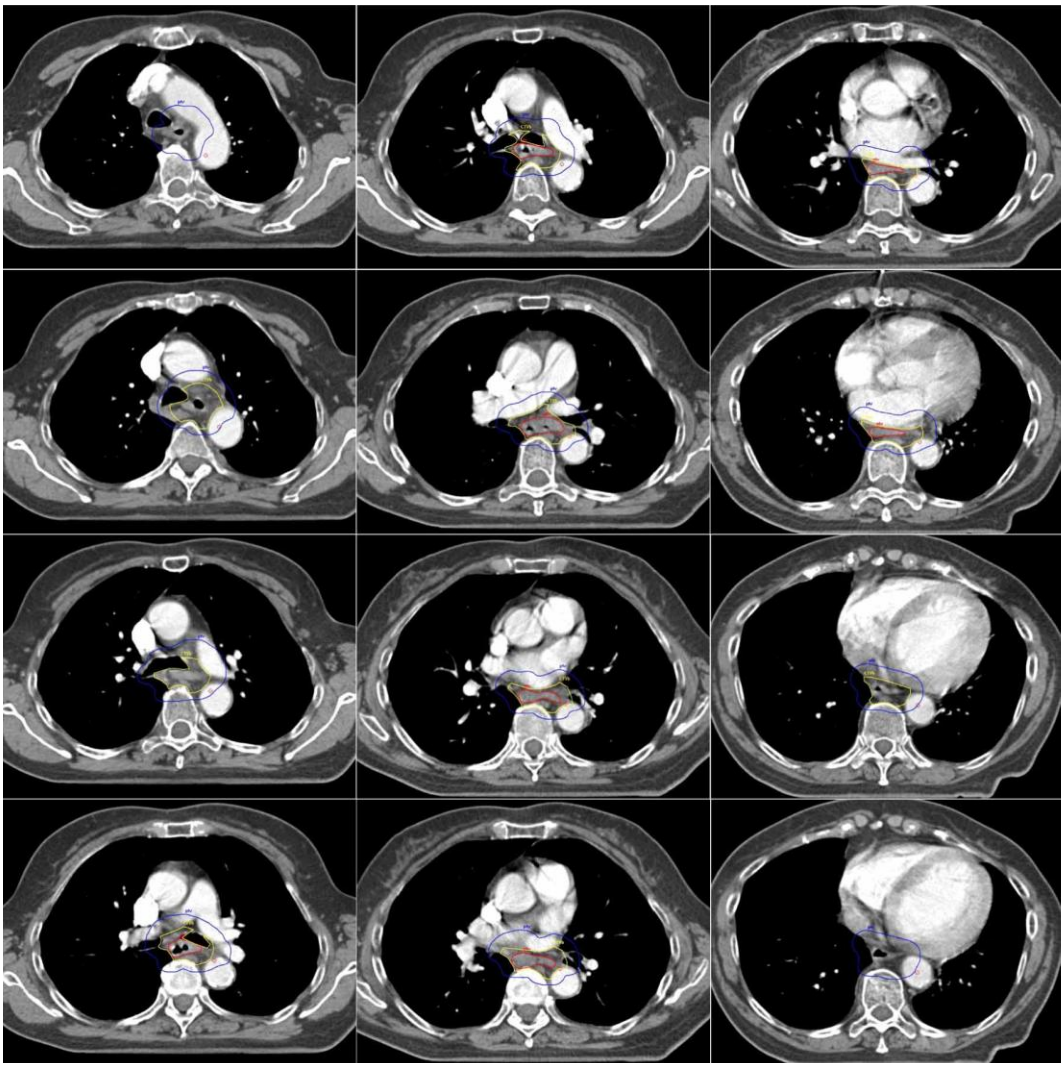

Appendix 4 *Delineation of Heart Volume*

The whole heart is outlined to the extent of the pericardial sac (if visible). The major blood vessels (superior to the organ) and the inferior vena cava (towards the inferior extent of the heart) are excluded. The superior extent is often difficult to define and may be simplified by identification of the vessels superior to the heart. We use the point where the pulmonary trunk and the right pulmonary artery are seen as separate structures as an indication of the superior extent of the heart.

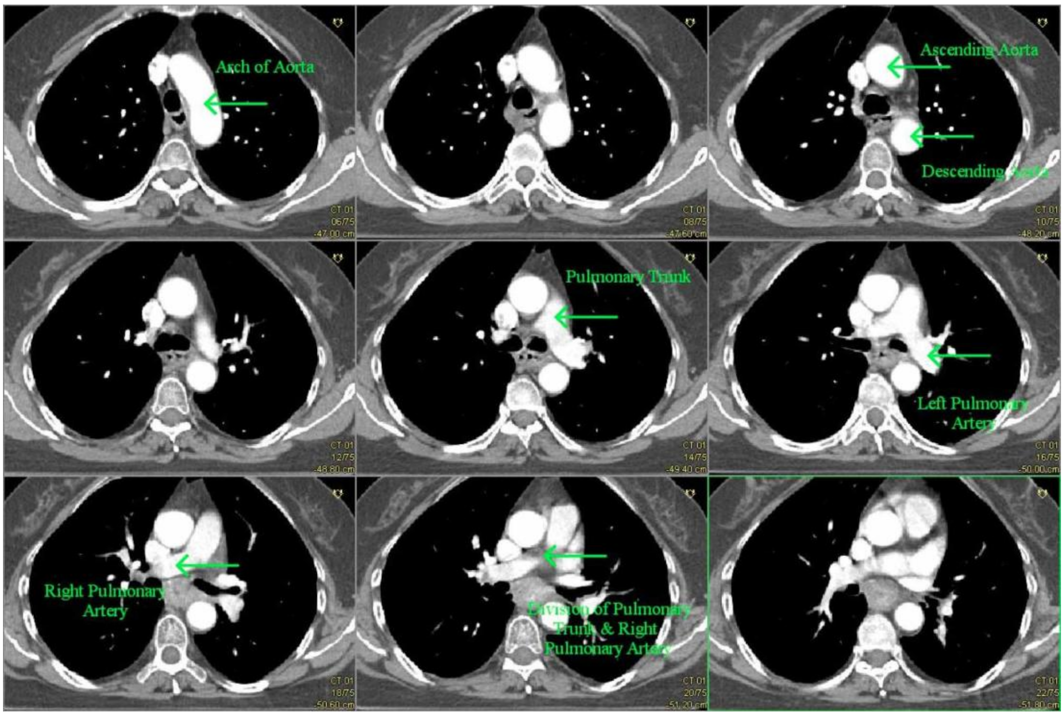

The definition of the heart is shown below on the same data set. Throughout, the heart is outlined to the extent of the pericardial sac. Inferiorly the organ is well defined compared to the surrounding tissues in the abdomen. However, if possible, the inferior vena cava should be excluded.

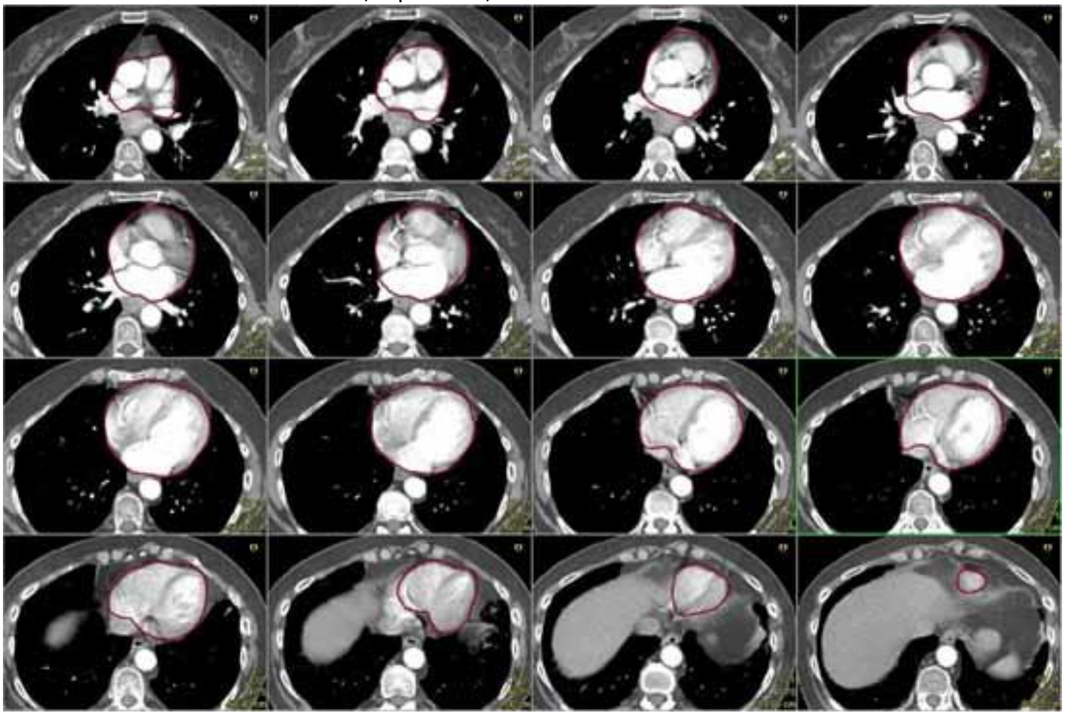

Appendix 5 *Delineation of stomach volume*

The stomach is outlined in its entirety from its proximal origin at the gastro-oesophageal junction to its distal extent at the pylorus–duodenal junction. Outlining is assisted by the use of digital image reconstruction, particularly in the coronal plane. At the gastro-oesophageal junction the stomach is identified on a contrast enhanced CT by a subtle increase in enhancement of the stomach mucosa. As the stomach descends it crosses the midline from left to right, and the wall thickness increases in the distal part (antrum and pylorus). The distal end is at the junction where the thick stomach wall changes to become the thinner duodenal wall.

Begin by contouring the stomach at the point where the GOJ passes the left crus of the diaphragm, and work up and then down.

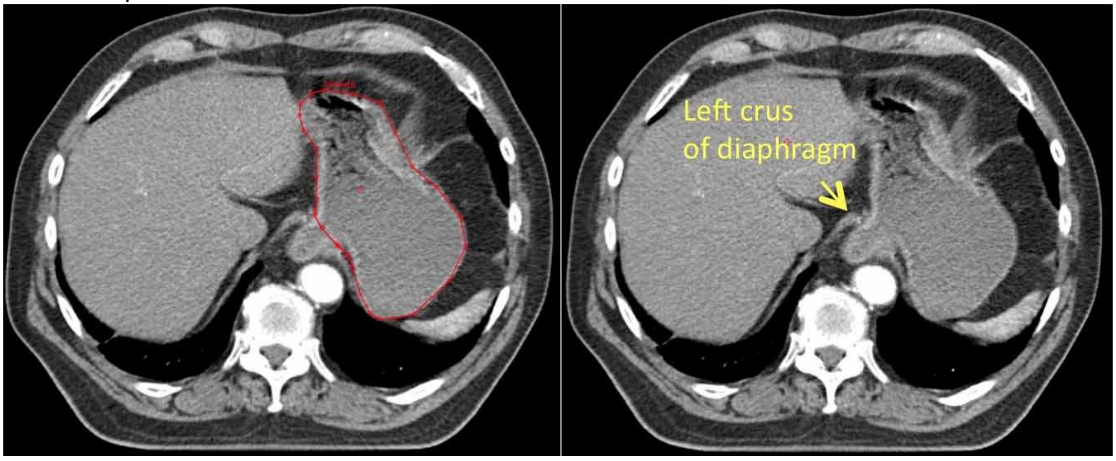

To identify the most distal extent, try to identify a change in wall thickness. The pylorus usually lies to the left of midline as shown:

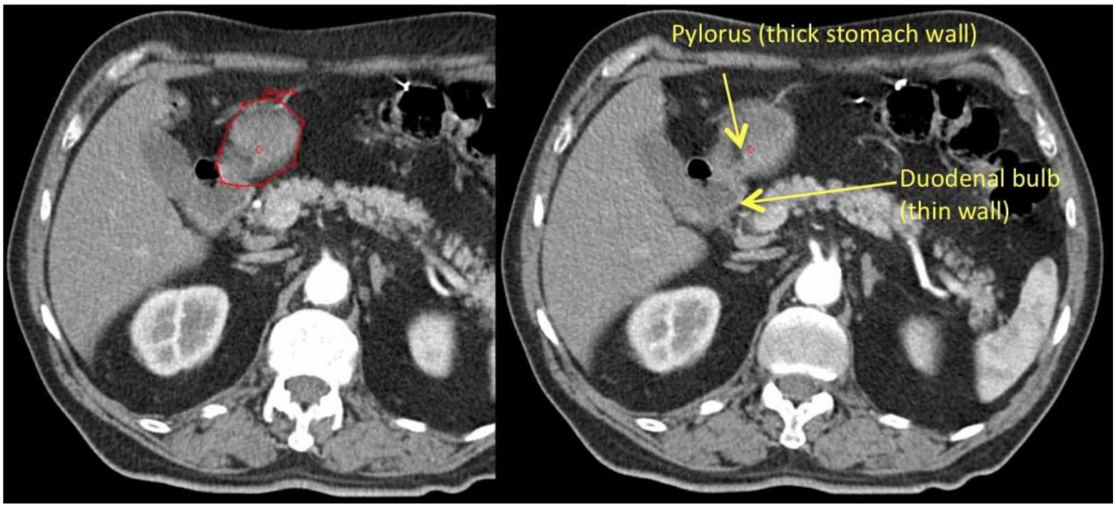

The contrast enhanced CT below shows the distinction between the stomach proximal to the pyloric sphincter muscles and duodenal bulb distally.

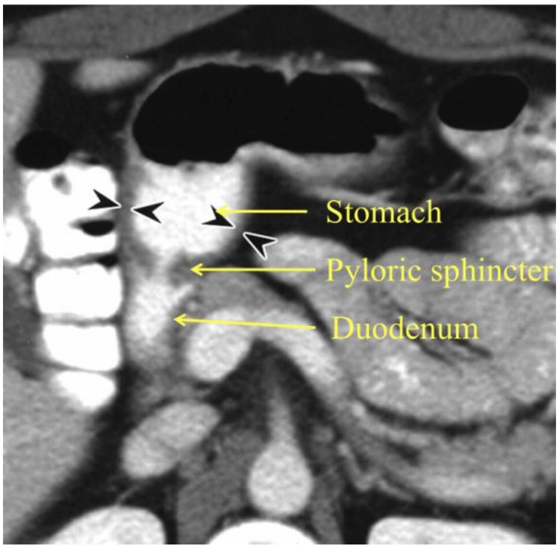

Example Stomach outlining

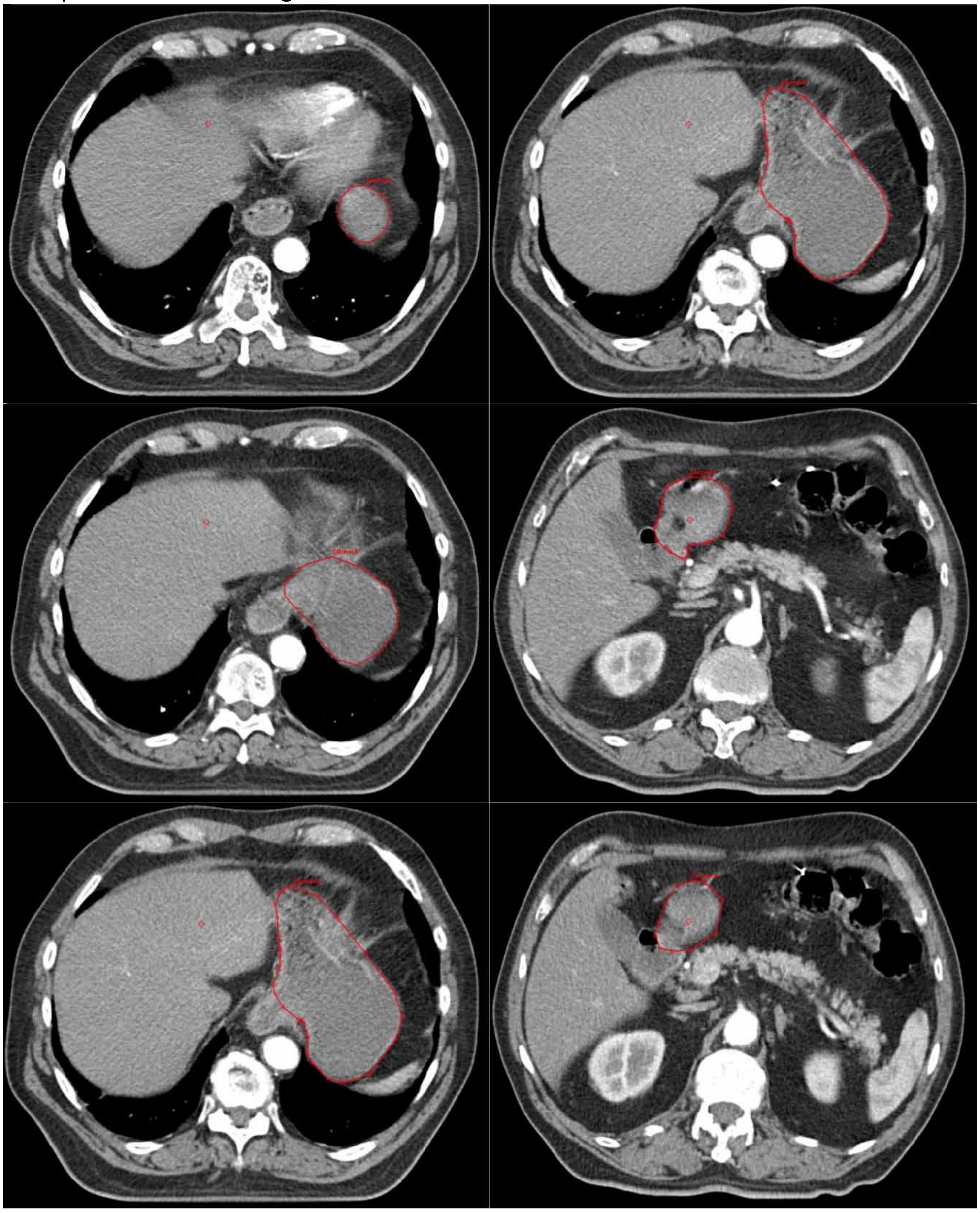

Appendix 6 Guidance on the use of contrast for 4D-CT

Depending on what scan functionality is available (in terms of 4DCT) it is recommended that a contrast enhanced 4D scan is acquired when the patient attends for their planning scan. Alternatively a contrast enhanced 3D helical scan (for planning) should be acquired. A 4D scan (to create the ITV) can be acquired before or after the 3d scan depending on the institutional protocol. In our experience the 4D scan should be acquired at the same scan session, ensuring minimal intra-scan patient movement for comparison of the 2 scan sets.. Guidelines for how to acquire a contrast enhanced 4D scan are given here, using a protocol which is established in Leeds.

Exact scan delays for 4DCT scans have not been accurately calculated in radiotherapy. Due to the extended scan times (slow couch travel) with these scans, the timings will significantly differ to those suggested for 3D scanning (as recommended by RCR_1). The timings suggested below have been calculated using an equation by Kyongtae.T, 2010_2.

Visualisation of the heart and azygousazygos vein are key so arterial and portal venous phase contrast enhancement is required.

Scan duration (SD) is **70s**, Injection duration (ID) is **34s** (3ml/s) or **50s** (2ml/s) for 100ml Niopam 300.

Peak enhancement of heart = $T_{(peak)} = T_{ID} + T_{CTT}$ (contrast transit time)

$T_{(peak)}$ = 34 + 15 so for 3ml/s: **49s**
$T_{(peak)}$ = 50 + 15 so for 2ml/s: **65s**

Peak enhancement of portal venous = 34 + 35 so for 3ml/s: **69s**
50 + 35 so for 2ml/s: **85s**

Scan delay = $T_{(delay)} = T_{(peak)} - ½\, T_{SD}$

Scan delay arterial = 49 – ½ 70 so for 3ml/s: **14s**
65 - ½ 70 so for 2ml/s: **30s**

Scan delay PV = 69 – ½ 70 so for 3ml/s: **34s**
85 – ½ 70 so for 2ml/s: **50s**

So to encompass both arterial phase into PV phase through a single bolus injection, suggested delay for a normal breathing scan protocol is:

3ml/s 25s
2ml/s 40s

For a slow breathing scan protocol (4D followed by 3D) the scan duration increases and so the delay should be reduced further to the order:

3ml/s 15-20s ***(safest minimum delay)***
2ml/s 25s

For further information and / or details for the scan acquisition processes described please contact Dave Smith (**David-W.Smith@leedsth.nhs.uk**), **Neil Roberts (Neil.Roberts@leedsth.nhs.uk) or Karen Askew (Karen.Askew@leedsth.nhs.uk)** at the St. James' Institute of Oncology

References

1. Button MR, Morgan CA, Croydon ES, Roberts SA, Crosby TD (2009). Study to determine adequate margins in radiotherapy planning for oesophageal carcinoma by detailing patterns of recurrence after definitive chemoradiotherapy. Int J Radiat Oncol Biol Phys. 2009 Mar 1;73(3):818-23. Epub 2008 Aug 19.

2. Dresner SM, Lamb PJ, Bennett MK et al. The pattern of metastatic lymph node dissemination from adenocarcinoma of the esophagogastric junction. Surgery 2001;129:103-9.).

3. Matzinger O, Gerber E, Bernstein Z, et al. EORTC-ROG expert opinion: Radiotherapy volume and treatment guidelines for neoadjuvant radiation of adenocarcinomas of gastroesophageal junction and the stomach. Radiother Oncol. 2009: 92; 164-175.

4. L. Wills, A. Millin, J. Paterson, T. Crosby and J. Staffurth. The effect of planning algorithms in oesophageal radiotherapy in the context of the SCOPE 1 trial. Radiotherapy and Oncology 2009:93(3);462-467.
