# Supplementary material for: NEOSCOPE: A randomised phase II study of induction chemotherapy followed by oxaliplatin/capecitabine or carboplatin/paclitaxel based pre-operative chemoradiation for resectable oesophageal adenocarcinoma
Source: Eur J Cancer. 2017 Mar;74:38–46. doi: 10.1016/j.ejca.2016.11.031 (PMC5341738; doi:10.1016/j.ejca.2016.11.031)
Supplement: Supplementary file 3 [file mmc3.docx]

**Supplementary Table 1. “Pick a winner” rule**

Upon completion of recruitment and follow up, the pCR rates and 30 day post- operative mortality rates will be calculated by arm. The following rules will be used to decide whether or not there is sufficient evidence to warrant a future phase III trial:

| 1 | If fewer than 10 patients achieve a pCR to either treatment, no treatment is taken forward to a phase III trial. |
| --- | --- |
| 2 | If 10 or more patients achieve a pCR to treatment A but fewer than 10 patients achieve a pCR to treatment B, treatment A is taken forward to a phase III trial. |
| 3 | If 10 or more patients achieve a pCR to treatment B, but fewer than 10 patients achieve a pCR to treatment A, treatment B is taken forward to a phase III trial. |
| 4 | If both treatments have 10 or more patients achieve a pCR, the treatment with higher response rate is taken forward to a phase III study provided the post-operative mortality is less than 5% in both arms. If post-operative mortality is > 5% for one of the treatments while the mortality is below 5% for the other, the treatment with the lower post-operative mortality is taken forward. |
| 5 | If both arms show high pCR and similar mortality then toxicities will be used to help decide which arm to take forward to a future phase III. |
