# Supplementary material for: NEOSCOPE: A randomised phase II study of induction chemotherapy followed by oxaliplatin/capecitabine or carboplatin/paclitaxel based pre-operative chemoradiation for resectable oesophageal adenocarcinoma
Source: Eur J Cancer. 2017 Mar;74:38–46. doi: 10.1016/j.ejca.2016.11.031 (PMC5341738; doi:10.1016/j.ejca.2016.11.031)
Supplement: Supplementary file 4 [file mmc4.pdf]

Appendix A

Sample size calculation under least favorable configuration

Thomas Jaki

Two novel pre-operative CRT regimens for resectable oesophageal cancer are to be compared and no control group is to be used. Here the sample size calculations based on the least favorable configuration are presented.

Denote the number of patients responding on CRT 1 and 2 by S_1 and S_2 respectively and write the probability of a response on each treatment as $p_i,\ i = 1, 2$. Then $S_i \sim Bin(n, p_i)$. The least favorable configuration is use to set the power requirement in trials comparing multiple treatments arms. It specifies the power such that one of the treatments (treatment 1, say) achieves the interesting effect size p, whilst the other treatments achieve an effect size of p_0. In other words the most promising treatment is just at the clinically relevant level while the others are as good as they can be without being of further interest. This specification implies that the power will be larger then the prespecified level if more than 1 treatment do have a clinically relevant effect.

Setting the clinically relevant effect to 0.35 and the not interesting effect at 0.15, we want

$$P(\text{take any treatment to Phase III}|p_1 = p_2 = 0.15) = P(max(S_1, S_2) > k|p_1 = p_2 = 0.15) \leq \alpha$$

and

$$P(\text{take any treatment to Phase III}|p_1 = p_2 = 0.35) = P(S_1 > k \text{ and } S_1 \geq S_2|p_1 = 0.35, p_2 = 0.15) \geq 1 - \beta$$

now

$$\begin{aligned} P\left(max(S_1, S_2) > k\right) &= P(S_1 > k \text{ or } S_2 > k) \\ &= P\left(S_1 > k\right)P(S_2 > k) \text{ as the subjects on each arm are different} \\ &= \sum_{i=k+1}^{n} \binom{n}{i} p_1^i (1 - p_1)^{n-i} \sum_{i=k+1}^{n} \binom{n}{i} p_2^i (1 - p_2)^{n-i} \end{aligned}$$

Using similar arguments we find

$$P(S_1 > k \text{ and } S_1 \geq S_2) = \sum_{i=k+1}^{n} \binom{n}{i} p_1^i (1 - p_1)^{n-i} \sum_{j=0}^{i} \binom{n}{j} p_2^j (1 - p_2)^{n-j}$$

Using the above distribution we can then solve the two equations above for n and k using R (code below). For $\alpha = 0.1$ and $1 - \beta = 0.8$ we find $n = 28$ and $k = 7$ while $n = 38$ and $k = 9$ is found for $1 - \beta = 0.9$. The probability of taking any of the two forward to phase III if both have a response rate of 0.35 is 0.967 and 0.991 respectively.

```
####
## Function for the maximum of two binomial random variables to be used
## for the type I error equation
####
maxdist <- function(k,n,p1,p2){
  pbinom(k,n,p1)*pbinom(k,n,p2)
}

####
## Function to find the power under the least favorable configuration
####
lfc <- function(k,n,p1,p2){

  sum <- 0
  for(i in (k+1):n){
    for(j in 0:i){
      sum <- sum + dbinom(i,n,p1)*dbinom(j,n,p2)
    }
  }
  sum
}

####
## Search for all solutions up to n=40 that satisfy both equations.
## Choose the one with smallest sample size.
####
for(n in 1:40){
  for(k in 0:n){

    alpha <- 1 - maxdist(k,n,0.15,0.15)
    power <- lfc(k,n,0.35,0.15)

    if(alpha <= 0.1 & power >= 0.9) print(c(n,k))

  }
}
```
